# Genomic Characterization and Multiplex PCR Authentication of Commercial Triploid Oysters: Evidence for Mosaic *Crassostrea gigas*–*C. angulata* Ancestry

**DOI:** 10.3390/foods15213921

**Published:** 2026-11-02

**Authors:** Kang-Rae Kim, Hye-Jin Kim, Yanping Qin, Yuehuan Zhang, Jong-Won Park, Jeong-Ho Lee, Hee-Jung Lee

**Affiliations:** 1 Southeast Sea Fisheries Research Institute, National Institute of Fisheries Science, Tongyeong 53085, Republic of Korea; kimkangrae9586@gmail.com (K.-R.K.); dapowind@korea.kr (J.-W.P.); jhlee7124@korea.kr (J.-H.L.); 2 Department of Life Science, Soonchunhyang University, Asan 31538, Republic of Korea; n_siho@naver.com; 3 Key Laboratory of Breeding Biotechnology and Sustainable Aquaculture (CAS), Key Laboratory of Tropical Marine Bio-Resources and Ecology, Guangdong Provincial Key Laboratory of Applied Marine Biology, South China Sea Institute of Oceanology, Chinese Academy of Sciences, Guangzhou 510301, China; qinyanping@scsio.ac.cn (Y.Q.); yhzhang@scsio.ac.cn (Y.Z.); 4 Agro-Tech Extension Center of Guangdong Province, Guangzhou 510145, China; 5 Daya Bay Marine Biology Research Station, Chinese Academy of Sciences, Shenzhen 518124, China

**Keywords:** triploid oyster, *Crassostrea angulata*, *Crassostrea gigas*, hybrid ancestry, local ancestry, whole-genome sequencing, multiplex PCR, seafood authentication

## Abstract

Triploid oysters are widely used in aquaculture, but their commercial species designation may not accurately represent their nuclear genomic composition, particularly when interspecific or polyploid broodstocks are involved. In this study, we combined whole-genome sequencing with a mitochondrial COI-based multiplex PCR assay to characterize commercial triploid *Crassostrea gigas*, *C. angulata*, and *C. hongkongensis*. Whole-genome allele-frequency analysis confirmed triploidy in all 15 individuals examined, comprising five individuals each from the A3N, G3N, and H3N groups. Cross-species ancestry dosage showed that G3N and H3N were predominantly assigned to their nominal species, whereas A3N exhibited mixed *C. angulata*–*C. gigas* nuclear ancestry, with approximately 2.2 and 0.7 haploid-equivalent copies assigned to these species, respectively. The minor *C. hongkongensis* assignment was interpreted as background cross-assignment rather than evidence of an additional ancestral contribution. Local-ancestry analysis further revealed chromosome-scale mosaics of zero, one, and two *C. gigas*-derived copies, with copy-weighted *C. gigas* contributions of approximately 0.69–0.88 haploid-equivalent copies among A3N individuals. This interspecific ancestry was supported by convergent evidence from complementary analyses of discrete dosage-state distributions, D-statistics with two alternative outgroups, and mapping-independent *C. gigas*-diagnostic 21-mers. All A3N individuals nevertheless carried *C. angulata* COI haplotypes, consistent with a *C. angulata* maternal lineage. The breeding history underlying the nuclear ancestry mosaics could not be resolved because the actual parental broodstock was unavailable for genomic analysis. A three-species multiplex PCR assay generated diagnostic products of 212, 359, and 169 bp for *C. gigas*, *C. angulata*, and *C. hongkongensis*, respectively, with no detectable cross-amplification at an optimized annealing temperature of 62 °C. Diagnostic amplification remained detectable with 1 ng of template DNA and was successfully applied to specimens from multiple Korean production sites and Dalian, China. These findings demonstrate complex nuclear ancestry in the five A3N individuals examined that was not captured by their nominal species designation or mitochondrial lineage alone. Although the observed ancestry proportions cannot be generalized to commercial A3N stocks as a whole, the results highlight the complementary roles of genome-wide ancestry analysis and rapid mitochondrial lineage authentication.

## 1. Introduction

Triploid oysters are widely used in hatchery-based aquaculture because triploidy generally reduces gametogenic activity and can provide advantages in growth, survival, and meat quality under appropriate culture conditions [1,2]. Triploids can be generated either by disrupting meiotic polar-body extrusion or by crossing tetraploid and diploid broodstock [2,3]. Experimental crosses in *Crassostrea gigas* demonstrated that mating tetraploids with diploids can produce essentially all-triploid surviving offspring and provide a more reliable route to triploid production than direct meiotic induction [3]. Consequently, the tetraploid-male × diploid-female breeding scheme has become an important strategy for producing triploid oyster seed [2,3].

The same polyploid breeding strategy can be extended to interspecific crosses, resulting in allotriploid oysters that inherit nuclear genomes from two parental species [2]. In *C. gigas* and *C. angulata*, reciprocal allotriploids have been experimentally produced by mating diploid females of one species with tetraploid males of the other, and their growth, survival, and reproductive characteristics differ according to cross direction and culture environment [2]. More recently, an allotriploid population produced specifically from tetraploid *C. gigas* males and diploid *C. angulata* females was examined for genetic diversity and population structure [4]. Reciprocal tetraploid hybrids between *C. gigas* and *C. angulata* have also been experimentally produced, demonstrating that tetraploid breeding material can itself contain interspecific genetic backgrounds [5]. In addition, genetically improved *C. gigas*–*C. angulata* allotriploids have been evaluated for growth, survival, reproductive traits, and aquaculture performance [6]. Together, these breeding developments indicate that the nuclear genomic composition of commercial triploid oysters may not always be adequately represented by a simple species designation or by ploidy status alone [4,5,6].

Accurate species identification is particularly challenging for *C. gigas* and *C. angulata* because the two taxa are closely related and exhibit limited morphological differentiation [7,8]. Mitochondrial analyses nevertheless distinguish the two lineages, and COI sequence divergence between *C. gigas* and *C. angulata* has been reported at approximately 2–3% [7]. The difficulty of morphology-based identification has had practical taxonomic consequences; for example, molecular analyses demonstrated that oysters historically regarded as *C. gigas* in Taiwan were actually *C. angulata* [8]. Oyster identification is further complicated by the high phenotypic plasticity of shell characters, which can vary with environmental and culture conditions and may therefore provide unreliable species assignments when closely related taxa are compared [8,9]. *Crassostrea hongkongensis*, in contrast, represents a more genetically differentiated lineage and was originally distinguished from related cupped oysters using mitochondrial COI and 16S sequence data together with morphological characters [10]. Molecular markers are therefore particularly useful for authentication of closely related *Crassostrea* taxa when morphology is ambiguous or unavailable [8,9,10].

Mitochondrial COI has previously been used to develop rapid molecular assays for *Crassostrea* identification [9]. Wang and Guo developed species-specific COI primers capable of distinguishing five Chinese *Crassostrea* species, including *C. gigas*, *C. angulata*, and *C. hongkongensis*, in a multiplex PCR format [9]. Such assays provide a practical alternative to sequence-based identification because species assignments can be made directly from diagnostic PCR product sizes [9]. However, mitochondrial and nuclear markers provide different information in interspecific breeding systems because mitochondrial DNA in *C. gigas* is maternally inherited [11]. Consequently, COI-based identification primarily reflects the maternal mitochondrial lineage and does not by itself resolve the genomic contribution of the paternal lineage to an interspecific triploid [11]. This distinction becomes particularly important in allotriploids, in which the nuclear genome contains unequal chromosome contributions from two parental species [2,3,4,5].

Previous studies of *C. gigas*–*C. angulata* allotriploids have primarily examined production traits, reproductive characteristics, or population-level genetic diversity [2,4,6]. For a triploid produced from a genetically homogeneous tetraploid *C. gigas* male and diploid *C. angulata* female, the simple breeding expectation is inheritance of two paternal *C. gigas*-derived chromosome copies and one maternal *C. angulata*-derived copy [3,4,5]. However, population-level marker analyses cannot determine whether such parental contributions are uniformly maintained along individual chromosomes or whether the triploid genome contains chromosome-scale variation in ancestry dosage [4]. This question is increasingly relevant because interspecific tetraploid and allotriploid breeding has created broodstock and offspring with potentially more complex genetic backgrounds than those predicted from nominal species labels alone [4,5,6]. Genome-wide analysis is therefore required to distinguish a simple fixed interspecific dosage from heterogeneous nuclear ancestry and to determine how such ancestry is distributed across the chromosomes of commercial triploid oysters.

In the present study, we combined whole-genome sequencing with mitochondrial COI-based multiplex PCR to characterize commercially produced triploid *C. gigas*, *C. angulata*, and *C. hongkongensis*. We first verified the ploidy of all individuals using genome-wide allele-frequency distributions and then characterized the nuclear genomic ancestry of A3N oysters using cross-species ancestry dosage, reciprocal reference mapping, genome-wide window analysis, hidden Markov model-based local ancestry inference, discrete dosage-state analysis, D-statistics with independent outgroups, and mapping-independent species-diagnostic k-mer analysis. In parallel, we developed and validated a three-species COI multiplex PCR assay for rapid authentication of the mitochondrial lineages of *C. gigas*, *C. angulata*, and *C. hongkongensis*. By integrating nuclear genomic ancestry with mitochondrial lineage authentication, this study aimed to determine whether the sampled commercial triploid individuals, particularly the five A3N oysters, conformed to the genomic composition expected under the proposed breeding model and to evaluate a practical molecular method for mitochondrial-lineage authentication. The genomic analysis focused on ancestry composition and chromosome-scale variation within the sampled individuals rather than estimating ancestry distributions across commercial stocks.

## 2. Materials and Methods

### 2.1. Sample Collection and Genomic DNA Extraction

Specimens of *C. gigas*, *C. angulata*, and *C. hongkongensis* were obtained from commercial markets and a hatchery in South Korea and China in August 2024. Sample sources, acquisition locations, and sampling dates are provided in Table S1. Initial species assignments were based on external shell morphology following published taxonomic descriptions and identification keys for *Crassostrea*. Because these characters overlap considerably among the three species, morphological assignments were treated as provisional, and COI sequencing was used to establish mitochondrial-lineage assignments prior to assay development.

Adductor muscle tissue was excised from each individual for molecular analysis. Specimens were initially held at −20 °C, which reflects the frozen state in which oyster products are typically distributed in commercial markets, and tissue subsamples were then transferred to 1.5 mL microtubes containing 99.9% ethanol. A freeze–ethanol preservation step was applied, in which frozen tissues were immersed in ethanol and held at 4 °C for three days before extraction. Immediately prior to isolation, tissues were rinsed with triple-distilled water (3DW) to remove residual ethanol. Genomic DNA was then extracted with the HiGene™ Genomic DNA Prep Kit (Biofact, Daejeon, Republic of Korea) following the manufacturer’s instructions, and DNA concentration and purity were assessed spectrophotometrically before use as PCR template.

### 2.2. Library Preparation and Whole-Genome Sequencing

Whole-genome sequencing was performed for 15 triploid oyster individuals, comprising five individuals each from the A3N (*C. angulata*), G3N (*C. gigas*), and H3N (*C. hongkongensis*) groups. Sequencing libraries were prepared using the xGen DNA Library Prep EZ Kit (Integrated DNA Technologies, Coralville, IA, USA) according to the manufacturer’s instructions. All 15 triploid libraries were sequenced together in a single sequencing run on the NovaSeq X platform (Illumina, Inc., San Diego, CA, USA) using a 150 bp paired-end (PE) strategy, generating approximately 15 Gbp of raw sequencing data per individual.

Raw sequencing reads were quality-checked and preprocessed using fastp v1.3.3 [12]. Adapter sequences and low-quality bases were removed prior to downstream analyses. Sequencing quality was evaluated based on Phred quality scores, with a Q30 proportion of at least 80% used as the minimum quality criterion. Only datasets meeting this quality threshold were retained for subsequent read alignment, variant calling, and genome-wide ancestry analyses.

### 2.3. Verification of Triploidy by k-mer-Free Allele-Frequency Analysis

Because the assay was intended for application to commercially produced triploid stocks, the ploidy of every individual used for primer design and validation was experimentally verified rather than inferred from hatchery designation. Whole-genome sequencing reads were generated from five triploid individuals of each target species, designated A3N (*C. angulata*), G3N (*C. gigas*), and H3N (*C. hongkongensis*). Three diploid reference panels were also included: ten *C. angulata* (2N), ten *C. gigas* (2N), and ten *C. hongkongensis* (2N) individuals (Table S2). Sequence data for these diploid specimens were retrieved from the NCBI Sequence Read Archive using the SRA Toolkit v3.2.1 [13].

To minimize reference bias caused by aligning reads from one species to the genome assembly of another, reads from each specimen were mapped to the reference assembly of the corresponding species. Paired-end reads were aligned using BWA-MEM v0.7.19-r1273 [14] against GCF_963853765.1 for *C. gigas*, GCA_025612915.1 for *C. angulata*, and GCA_015776775.1 for *C. hongkongensis*. Read-group information was assigned during alignment. The resulting alignment files were processed using SAMtools v1.23.1 [15], including mate-information correction with fixmate, coordinate sorting, duplicate marking, and BAM indexing.

Ploidy was inferred from the distribution of allele frequencies at biallelic heterozygous sites using nQuire [16] from allelic read-frequency distributions at biallelic heterozygous sites in the alignment files, rather than from k-mer multiplicity distributions. This alignment-based ploidy assessment was separate from the mapping-independent diagnostic 21-mer ancestry analysis described in Section 2.5.7. For each individual, nQuire [16] create was run with a minimum site coverage of six reads and a minimum minor-allele fraction of 0.15. The resulting binary site files were processed using nQuire [16] denoise to remove the uniform error component from the allele-frequency spectrum, and the denoised spectra were exported using nQuire histo [16].

The minimum coverage threshold of six reads was selected following a sensitivity analysis in which representative high-coverage individuals of each ploidy and reference genome were re-analysed using thresholds of six and ten reads. Increasing the threshold to ten reads removed a substantial proportion of informative sites from individuals with intermediate sequencing depth, reducing the number of callable positions by approximately half, while leaving the ploidy assignments of high-coverage individuals unchanged. A threshold of six reads was therefore applied consistently to all individuals to ensure that every specimen was evaluated using the same criteria.

Ploidy was evaluated from the shape of the denoised allele-frequency spectrum. In triploid genomes, heterozygous sites are expected to exhibit alternate-allele frequencies near 1/3 and 2/3, whereas heterozygous sites in diploid genomes are expected to cluster near 1/2. A summary statistic was calculated as the ratio of the spectral density within the 31–35% and 65–69% allele-frequency intervals to that within the 48–52% interval. Because the magnitude of this statistic differed among species, ploidy was evaluated by comparison with the corresponding species-matched diploid reference panel rather than by applying a universal decision threshold.

### 2.4. Reciprocal Mapping and Ancestry Dosage Analysis of Interspecific Triploids

Species-diagnostic nuclear variants between *C. angulata* and *C. gigas* were identified exclusively from the diploid reference panels to ensure that marker ascertainment was independent of the triploid individuals subsequently analysed. Candidate sites were required to be fixed for alternative alleles between the ten diploid *C. angulata* and ten diploid *C. gigas* individuals. Triploid A3N individuals were not used during diagnostic-site selection.

Whole-genome reads from the triploid individuals were aligned to the *C. angulata* reference genome (GCA_025612915.1) using BWA-MEM v0.7.19-r1273 [14], and alignment files were processed as described above. Variant calling was performed with GATK v4.6.2.0 [17] HaplotypeCaller in reference-confidence mode with sample ploidy set to three, followed by joint genotyping using GenomicsDBImport and GenotypeGVCFs. Reference and alternative allele depths were extracted at the diagnostic positions and reoriented such that allele identity was consistently defined by species of origin.

Because differential alignment efficiency between *C. angulata*- and *C. gigas*-derived reads could influence observed allele fractions, reference-dependent mapping bias was evaluated using reciprocal alignment to the *C. gigas* reference genome (GCF_963853765.1).

Species-diagnostic markers were retained only if they were biallelic single-nucleotide sites with QUAL ≥ 30 at which the two diploid reference panels were fixed for alternative alleles. A marker was accepted at a “moderate” tier when at least seven individuals per panel had valid genotypes (read depth ≥ 8 and genotype quality ≥ 20) and at least 80% of those individuals carried the expected panel-specific allele, and at a “strict” tier when at least eight individuals per panel met these criteria with panel alternative-allele frequencies ≤0.02 or ≥0.98 and ≥90% allele concordance. At each marker the focal-species (B) allele was defined by species of origin, so that a read either supported or opposed the B allele irrespective of whether that allele was the reference or the alternative base. The sequential filtering criteria and numbers of retained SNPs are provided in Table S3.

Reference-dependent mapping bias was corrected for each individual using the reciprocal orientation of the diagnostic markers. For each individual the B-allele read fraction was pooled separately over two marker classes—markers at which the B allele is the alternative base, yielding p_ALT, and markers at which the B allele is the reference base, yielding p_REF—with each fraction computed as (Σ B-allele reads + 0.5)/(Σ total reads + 1). Because reference-mapping bias inflates reference-allele reads in both orientations by the same multiplicative factor, it displaces p_ALT and p_REF in opposite directions on the logit scale; the unbiased B-allele fraction q was therefore recovered as the midpoint of the two logits, q = logit^−1^{[logit(p_ALT) + logit(p_REF)]/2}, and the individual-specific alternative-versus-reference read efficiency was estimated as b = exp{[logit(p_ALT) − logit(p_REF)]/2}. An individual-specific correction is necessary because alignment efficiency depends on each individual’s read length, coverage, and divergence from the reference, so that a single global factor would leave residual, sample-specific bias. The bias-corrected ancestry copy number was calculated as q multiplied by the sample ploidy (three for triploids and two for diploid reference individuals). Diploid reference panels and predominantly single-species triploid groups were included as positive and negative controls for evaluating the behaviour of the estimator. To evaluate low-level *C. hongkongensis* assignments, the estimator was applied to all diploid and triploid groups using both the *C. gigas*–*C. hongkongensis* and *C. angulata*–*C. hongkongensis* diagnostic-marker panels. Diploid species not used for marker ascertainment in each pairwise comparison were included to assess background cross-assignment beyond the ascertainment panels. Bias-corrected allele fractions were expressed on a common three-copy scale (3q) for comparison between diploid and triploid groups, and moderate- and strict-tier estimates were compared (Table S12).

### 2.5. Genome-Wide Ancestry Architecture of A3N Triploids

#### 2.5.1. Genome-Wide Window-Based Ancestry Analysis

To assess the genomic distribution of *C. gigas* ancestry, the 3623 moderate-tier *C. gigas*–*C. angulata* diagnostic SNPs defined on the *C. angulata* reference assembly were partitioned into non-overlapping 5 Mb windows across the ten reference chromosomes (Table S3). Chromosome-level diagnostic-marker counts and densities are provided in Table S4. Within each window, the *C. angulata*-derived allele fraction was calculated as the depth-weighted proportion of *C. angulata*-derived reads pooled across all diagnostic sites, and the corresponding *C. gigas* fraction was calculated as its complement. Windows containing fewer than ten diagnostic sites were excluded. Diagnostic-marker counts, marker availability in A3N read data, and inclusion in the window-based analysis are reported for each window in Table S5.

For each group, variation among windows was quantified as the standard deviation of the window-level ancestry estimates. To determine whether this variation exceeded that expected from sequencing depth alone, the observed standard deviation was compared with the standard deviation expected under binomial sampling at the observed read depths, and an overdispersion ratio was calculated as the observed standard deviation divided by the expected binomial standard deviation. The target triploid group (A3N) was analysed together with the predominantly single-species triploid groups (G3N and H3N) and the diploid reference panels to distinguish ancestry-related genomic heterogeneity from variation attributable to sequencing or ploidy.

Two complementary measures of reproducibility were also calculated. First, diagnostic sites within each chromosome were ordered by genomic position and divided into odd- and even-ranked subsets, from which window-level ancestry estimates were independently recalculated. Pearson correlation between the two resulting window profiles was used as a split-half reliability measure. Second, pairwise correlations of window-level ancestry profiles were calculated among individuals within each group and averaged to quantify inter-individual concordance. A high split-half correlation accompanied by low inter-individual correlation was taken to indicate reproducible, individual-specific genomic ancestry structure rather than random measurement error or a systematic shared artefact.

#### 2.5.2. Cross-Species Ancestry Dosage for Triploid Composition

The genomic composition of each triploid group was assessed using moderate-tier pairwise diagnostic-marker panels defined on the *C. gigas* reference assembly GCF_963853765.1: 3726 *C. gigas*–*C. angulata* SNPs, 13,282 *C. gigas*–*C. hongkongensis* SNPs, and 6026 *C. angulata*–*C. hongkongensis* SNPs. Strict-tier estimates were additionally evaluated for the *C. hongkongensis* background-control analysis (Table S12). In addition to the *C. gigas* versus *C. angulata* comparison used for the A3N analysis (Section 2.4), species-diagnostic sites were defined for the two remaining species pairs, *C. gigas* versus *C. hongkongensis* and *C. angulata* versus *C. hongkongensis*, using the corresponding diploid reference panels. For each pair, candidate diagnostic variants were required to be fixed for alternative alleles between the two diploid panels, and reads from every triploid and diploid individual were mapped to both parental reference assemblies using the alignment procedure described in Section 2.3 and the reciprocal-mapping approach described in Section 2.4. Reference and alternative allelic depths were extracted at the diagnostic sites, reoriented to a consistent focal species, evaluated for reference-dependent mapping bias using the reciprocal-mapping approach described in Section 2.4, and converted to a copy number by multiplying the bias-corrected ancestry fraction by three. For each triploid group, the three-species copy-number vector was then assembled from these comparisons. The *C. hongkongensis* copy number was taken from the *C. gigas* versus *C. hongkongensis* comparison, and the *C. gigas* copy number from the *C. gigas* versus *C. angulata* comparison; for the *C. hongkongensis* triploid group, whose reads are not informative at *C. gigas* versus *C. angulata* diagnostic sites, the *C. gigas* copy number was instead defined as the non–*C. hongkongensis* complement of the *C. gigas* versus *C. hongkongensis* comparison. The *C. angulata* copy number was obtained as the residual required for the three species to sum to three haploid-equivalent copies, that is, *C. angulata* = 3 − (*C. gigas* + *C. hongkongensis*), and the *C. angulata* versus *C. hongkongensis* comparison was used as an independent consistency check. A group derived from a single species is expected to carry approximately three copies of that species and approximately zero copies of the others, whereas an interspecific triploid is expected to carry non-zero copies of two species. The diploid reference panels (two copies of the focal species) served as positive and negative controls for each pairwise comparison.

The resulting three-species ancestry vector was constrained to sum to three haploid-equivalent copies. The independent *C. angulata*–*C. hongkongensis* comparison was used as a consistency check rather than as an additional component of the final ancestry vector.

#### 2.5.3. Local Ancestry Tract Inference

Local ancestry was inferred separately for each triploid individual using the same 3623 moderate-tier *C. gigas*–*C. angulata* diagnostic SNPs used for the window-based analysis, retaining sites with read depth ≥6 in the corresponding individual. The three-state hidden Markov model (HMM) represented zero, one, or two *C. gigas*-derived chromosome copies, with expected *C. gigas* read fractions mu_k of 0.02, 1/3, and 2/3, respectively (the zero-copy mean was set to 0.02 to accommodate a small background level of non-target reads). At each diagnostic site, the observed *C. gigas* read fraction was modelled using a Gaussian emission density. The expected read fractions were 0.02, 1/3, and 2/3 for states representing zero, one, and two *C. gigas*-derived copies, respectively. For each state, the emission standard deviation was calculated by taking the square root of the expected binomial variance divided by site read depth, with the binomial variance term floored at 0.01, and then adding 0.05. Specifically, the calculation was sqrt[max(mu × (1 − mu), 0.01)/max(depth, 1)] + 0.05, where mu denotes the expected read fraction for the state. The three states were assigned equal initial probabilities of 1/3. Emission densities therefore varied with sequencing depth rather than being specified by a fixed discrete emission matrix. Transition probabilities depended on the physical distance between consecutive diagnostic markers. A distance-dependent relaxation term was calculated as the smaller of 0.5 and the inter-marker distance in base pairs multiplied by 0.00000002. The probability of remaining in the same ancestry state was calculated as 0.995 minus this relaxation term, with a minimum value of 0.5. The remaining probability was divided equally between transitions to the other two states. The same transition rule was applied to all three ancestry states. The distance-dependent coefficient was a spatial regularization parameter rather than a biological recombination rate. The model did not assume a specified number of generations since hybridization, an effective population size, or an F1/F2 pedigree. It was used to segment local ancestry-dosage patterns rather than reconstruct breeding history.

The Gaussian emission was used as a depth-scaled approximation to binomial read sampling, with an additive overdispersion term to accommodate variation beyond that expected from read sampling alone. The zero-copy mean was set to 0.02 rather than exactly zero so that the small background of non-target reads at true zero-copy sites is not treated as an essentially impossible observation, and the additive term of 0.05 absorbs overdispersion beyond binomial sampling—for example from paralogous alignment or local coverage variation—preventing over-confident state calls at high depth.

The distance-dependent parameter λ controlled spatial persistence between informative markers and was not interpreted as a biological recombination rate. The transition model did not assume a specified number of generations since hybridization, an effective population size, or an F1/F2 pedigree. It was used to segment read-fraction patterns into local ancestry-dosage states rather than to reconstruct demographic history. Consequently, inferred transition intervals remain dependent on marker spacing and model parameterization and should not be interpreted as exact historical recombination breakpoints. A three-state model representing zero, one, or two *C. gigas* copies was used to evaluate local ancestry under the reported breeding model of a *C. angulata* maternal lineage. The three-copy *C. gigas* state was not included in the HMM and was instead evaluated separately as a model-consistency diagnostic (Section 2.5.5). All emission and transition parameters were fixed a priori from this read-sampling model rather than fitted to the triploid data; the robustness of the resulting ancestry boundaries was assessed by the segment-support filters described below, and the copy-weighted ancestry fraction derived from the HMM was cross-checked against the mapping-based dosage, D-statistic, and k-mer estimates, none of which depend on the HMM.

The most probable ancestry-state sequence was inferred independently for each individual and chromosome using Viterbi decoding. Consecutive diagnostic sites assigned to the same state were merged into contiguous ancestry segments, and chromosome-wide ancestry paintings were generated for all ten chromosomes. The distribution of distances between adjacent moderate-tier diagnostic markers is summarized in Table S7.

To evaluate the robustness of inferred ancestry transitions, segment boundaries were recalculated after sequentially excluding short or weakly supported segments. Filters required segments to contain at least three or five diagnostic sites and, in additional analyses, to span at least 1 or 2 Mb. The persistence of chromosome-scale ancestry transitions across these increasingly stringent filters was used to distinguish robust ancestry tracts from transitions supported only by isolated or sparsely distributed markers.

*C. gigas* ancestry within HMM-assigned segments was summarized as a copy-weighted fraction, (f1 + 2f2)/3, where f1 and f2 represent the proportions of the summed segment span assigned to the one-copy and two-copy states, respectively. Segment span was defined as the distance between the first and last supporting markers. Unassigned intervals and chromosome ends beyond the outermost markers were excluded from the denominator. The corresponding haploid-equivalent dosage was calculated as three times the copy-weighted fraction (Table S8).

#### 2.5.4. Discrete Dosage-State Analysis to Distinguish Ancestry from Low-Level DNA Contamination

To distinguish genuine chromosomal incorporation of *C. gigas* ancestry from low-level exogenous DNA contamination, *C. gigas* read fractions were examined at moderate-tier *C. gigas*–*C. angulata* diagnostic sites on the *C. angulata* reference assembly, retaining individual-site observations with read depth ≥20. The same marker tier and depth threshold were used for the three-copy-state assessment in Section 2.5.5. For each site, the observed *C. gigas* read fraction was assigned to the nearest expected triploid dosage state (0, 1/3, 2/3, or 1), and the absolute deviation from that expected state was calculated.

Under a germline ancestry model, allelic fractions are expected to cluster around the discrete dosage ratios corresponding to zero, one, two, or three *C. gigas* copies. In contrast, low-level contamination is expected to produce a broadly distributed or systematically intermediate minor-allele signal rather than repeated clustering around triploid dosage ratios. Accordingly, the distribution of observed read fractions relative to the expected triploid dosage states was quantified for each individual.

#### 2.5.5. Maternal-Lineage Consistency Test

The mitochondrial lineage of each A3N individual was determined from its COI haplotype and used to evaluate consistency between mitochondrial and nuclear ancestry. Because mitochondrial DNA is maternally inherited, the *C. angulata* mitochondrial lineage was used to evaluate nuclear ancestry under a model retaining one maternally derived *C. angulata* chromosome copy. Under this model, the expected nuclear ancestry states are AAA, AAG, and AGG, corresponding to zero, one, and two *C. gigas* copies, respectively. A three-copy *C. gigas* state (GGG) would therefore be inconsistent with this maternal-lineage model.

High-confidence diagnostic sites assigned to the GGG state were therefore examined separately. For each individual, the frequency of GGG calls was quantified and compared with the frequencies of the AAA, AAG, and AGG states. To determine whether GGG calls represented genuine genomic segments or isolated errors, their sequencing depth was compared with the genome-wide depth distribution, and cross-individual recurrence was quantified as the number of individuals sharing each GGG-assigned diagnostic site. In addition, consecutive diagnostic sites assigned to the same state were grouped into runs, and run-length distributions were compared among the zero-, one-, two-, and three-copy states. A genuine three-copy ancestry component would be expected to form recurrent multi-marker tracts, whereas isolated, non-recurrent GGG calls were interpreted as inconsistent with chromosome-scale ancestry.

#### 2.5.6. D-Statistics, Synthetic Calibration, and Outgroup Cross-Validation

Genome-wide excess allele sharing between the target triploids and *C. gigas* was evaluated using the ABBA–BABA framework. This analysis used genome-wide variants without restriction to the diagnostic-marker panel used for ancestry estimation, providing a complementary assessment of allele-sharing asymmetry between A3N and *C. gigas*.

D-statistics were calculated for the topology D(P1 = *C. angulata*, P2 = target (A3N), P3 = *C. gigas*, O = outgroup) [18,19]. Because the reference/alternative labelling of a variant call does not correspond to the ancestral versus derived state, allele frequencies were not polarized by an inferred ancestral allele; instead, the standard frequency-based estimator of Durand et al. [19] was used, in which the per-site ABBA and BABA contributions include both allelic orientations, ABBA = (1 − p1)·p2·p3·(1 − pO) + p1·(1 − p2)·(1 − p3)·pO and BABA = p1·(1 − p2)·p3·(1 − pO) + (1 − p1)·p2·(1 − p3)·pO, where p1, p2, p3, and pO are the group alternative-allele frequencies, and were summed genome-wide to obtain D = (ΣABBA − ΣBABA)/(ΣABBA + ΣBABA). Because both orientations are included, this estimator is invariant to exchange of the reference and alternative alleles and therefore requires no ancestral-state polarization; when the outgroup is fixed for a single allele, it reduces to the conventional single-orientation form. The same estimator was applied to the empirical data and to the synthetic calibration series described below.

For every group, p1, p2, p3, and pO were computed as the alternative-allele frequency—the number of alternative alleles divided by the number of called alleles across all individuals of the group—and any site at which one or more of the four groups had no called allele was discarded. Only biallelic single-nucleotide sites from the reciprocal-mapping GATK joint callset were used, and standard errors and Z-scores were obtained by a 5 Mb block jackknife across the genome. As internal controls evaluated with the identical estimator, the contrast that splits a single *C. angulata* panel, D(P1 = *C. angulata*_a, P2 = *C. angulata*_b, P3 = *C. gigas*, O = outgroup), was expected to be approximately zero, whereas the positive control substituting the predominantly *C. gigas* triploid group (G3N) for P2 was expected to be strongly positive.

Two independent outgroups were used to evaluate the robustness of the results to outgroup choice: *C. hongkongensis* and *C. virginica*. For the latter, whole-genome sequencing reads from ten *C. virginica* individuals were obtained from the NCBI Sequence Read Archive (SRR24056612, SRR24056615, SRR24056605, SRR24056608, SRR24056600, SRR24056594, SRR24056588, SRR24056583, SRR24056577, and SRR24056651). Reads were downloaded using SRA Toolkit v3.2.1, aligned to the *C. gigas* reference genome (GCF_963853765.1) using the procedure described in Section 2.3, and jointly genotyped with the study samples using region-sharded GATK v4.6.2.0 GenomicsDBImport and GenotypeGVCFs workflows [17].

Because the magnitude of the D-statistic reflects allele-sharing asymmetry rather than chromosome copy number directly, a synthetic calibration series was generated separately for each analysed dataset. Diploid *C. angulata* and *C. gigas* allele frequencies were combined in fixed proportions corresponding to expected *C. gigas* contributions ranging from 0.0 to 1.0 copies in a triploid genome. D was calculated for each synthetic mixture using the same sites and outgroup as in the corresponding empirical analysis. Observed A3N values were then interpreted relative to these calibration curves rather than as direct estimates of copy number. Analyses were performed both for the pooled A3N group and separately for each A3N individual.

#### 2.5.7. Mapping-Independent k-mer Ancestry Analysis

A mapping-independent assessment of species ancestry was performed using canonical 21-mers counted directly from sequencing reads with Meryl v1.4.2 [20]. This analysis quantified diagnostic sequence content relative to the diploid reference panels and G3N controls; it did not infer ploidy from k-mer multiplicity peaks or estimate genomic heterozygosity or repeat content. Singleton k-mers were removed to reduce the contribution of sequencing errors. Reference genome assemblies were not used to anchor or validate these diagnostic k-mer counts. No additional repeat-specific filtering or upper k-mer multiplicity cutoff was applied before diagnostic k-mer quantification.

Species-specific k-mer sets were defined using the diploid reference panels. *C. gigas*-diagnostic k-mers relative to *C. angulata* were defined as canonical 21-mers detected in all diploid *C. gigas* individuals and absent from the pooled diploid *C. angulata* panel. *C. angulata*-diagnostic k-mers were defined reciprocally. These diagnostic k-mer sets were subsequently intersected with the k-mer spectrum of each triploid and diploid individual.

Because *C. gigas* and *C. angulata* are closely related, the number of strictly species-diagnostic k-mers is limited and raw diagnostic k-mer fractions were therefore not interpreted directly as genome-wide ancestry proportions. Instead, the number of distinct *C. gigas*-diagnostic k-mers detected in each individual was normalised relative to the *C. gigas* triploid control group (G3N). A complementary depth-weighted k-mer signal was calibrated against the same G3N panel and expressed on a relative *C. gigas* copy-dosage scale. The diploid *C. angulata* reference panel was included as a negative control for both measures.

#### 2.5.8. Software and Computational Implementation

Sequence reads were aligned using BWA-MEM v0.7.19-r1273 [14], and alignment files were processed with SAMtools v1.23.1 [15]. Variant calling and joint genotyping were performed using GATK v4.6.2.0 [17], and variant records and allele-depth information were processed using BCFtools v1.23.1 [21]. Canonical k-mer spectra were generated using Meryl v1.4.2 [20], and publicly available sequencing reads were retrieved using SRA Toolkit v3.2.1 [13].

All custom analyses described in Section 2.5.1, Section 2.5.2, Section 2.5.3, Section 2.5.4, Section 2.5.5, Section 2.5.6 and Section 2.5.7, including window-based ancestry estimation, HMM-based local ancestry inference, dosage-state analysis, maternal-lineage consistency testing, D-statistic calculation with block-jackknife resampling and synthetic calibration, and mapping-independent k-mer quantification, were implemented in Python v3.11.3. Numerical and statistical calculations were performed using NumPy v1.26.4 [22], SciPy v1.15.3 [23], and pandas v2.2.3 [24], and figures were generated using Matplotlib v3.10.0 [25].

The analytical procedures described above were consolidated into 3NdeTECT v0.2.0 [26], a taxon-animalia Snakemake workflow for whole-genome sequencing-based triploidy assessment and interspecific ancestry analysis. The workflow, configuration templates, environment specifications, and source code are publicly available on GitHub (https://github.com/krkim1989/3NdeTECT, accessed on 18 August 2026) [26]. 3NdeTECT is provided as a reproducible implementation of the analytical framework applied in this study rather than as a universally validated ploidy-classification tool.

### 2.6. Design of Species-Specific Primers for Three Crassostrea Species

Reference COI sequences for the three target species were downloaded from GenBank. For *Crassostrea gigas*, accessions EU672831.1 and KJ855241.1–KJ855245.1 were used; for *C. angulata*, NC_012648.1, FJ841965.1, KJ855246.1, KJ855248.1, and KJ855249.1; and for *C. hongkongensis*, NC_011518.2, EU266073.1, EU672834.1, FJ593172.1, FJ593173.1, FJ841963.1, and ON720160.1. To these, we added COI sequences determined here from five individuals per species (A3N01–A3N05, G3N01–G3N05, H3N01–H3N05). All sequences were aligned, and positions that were invariant within the sequences examined for one species and consistently different from the corresponding positions in the other two species were treated as candidate diagnostic sites. Thus, within-species variation at candidate primer-binding sites was assessed using the 15 study-derived and 18 GenBank COI sequences included in the alignment. This assessment was restricted to the listed sequences and did not constitute a comprehensive population-level survey of primer-binding variation.

Three primer pairs were then constructed, one per species, each anchored on such diagnostic positions. Wherever possible, the mismatch against non-target species was placed within the last three bases of the 3′ terminus, since extension is most sensitive to mismatches at this position. Primer length, GC content, melting temperature, and potential hairpin, self-dimer, and cross-dimer formation were evaluated using PCR Primer Stats in the Sequence Manipulation Suite (https://www.bioinformatics.org/sms2/pcr_primer_stats.html, accessed on 2 August 2026).

### 2.7. Multiplex PCR Amplification

Amplifications were run in a total volume of 20 µL with AccuPower^®^ Multiplex PCR Pre-mix (Bioneer Inc., Daejeon, Republic of Korea) on a Bio-Rad thermal cycler (Bio-Rad Laboratories, Hercules, CA, USA). Each 20 µL reaction contained 10 ng of template DNA, with each primer added at 10 pmol per reaction, corresponding to a final concentration of 0.5 µM. The cycling profile began with denaturation at 94 °C for 5 min, followed by 30 cycles of 94 °C for 30 s, 62 °C for 30 s, and 72 °C for 30 s, and closed with a 7 min extension at 72 °C. Every run included a control reaction in which nuclease-free water substituted for template. Products were electrophoresed through 1.5% agarose against a 100 bp ladder and imaged under UV light.

### 2.8. Annealing Temperature

Five annealing temperatures were compared, namely 55, 57, 59, 60, and 62 °C. Apart from the annealing temperature, all reaction conditions followed Section 2.7. Amplification products were evaluated for the presence of the expected target band and any non-specific amplification products.

### 2.9. Sensitivity to Template DNA Concentration

Three template inputs, 50, 10, and 1 ng per reaction, were tested for each target lineage, with PCR reactions performed in triplicate at each input. All other reaction conditions followed Section 2.7. Amplification was assessed qualitatively according to the presence of an amplicon of the expected size rather than band intensity. The number of positive reactions was recorded for each lineage and template input. Mitochondrial-lineage identification was based on the presence of an amplicon of the expected size rather than band intensity.

### 2.10. Validation on Field and Market Specimens

Once optimized, the assay was tested against individual oysters spanning several production sites and both ploidy levels. These comprised triploid *C. gigas* from Jindo cage culture, Jindo offshore cage culture, Goheung, and two Namhae sites (Seolcheon and Dolseom), along with triploid *C. hongkongensis*, triploid *C. angulata*, and paired diploid and triploid *C. gigas* from Dalian, China. Reactions were performed on each specimen separately, and mitochondrial lineages were assigned according to amplicon size.

## 3. Results

### 3.1. Whole-Genome Sequencing and Verification of Triploidy

**Figure 1:**
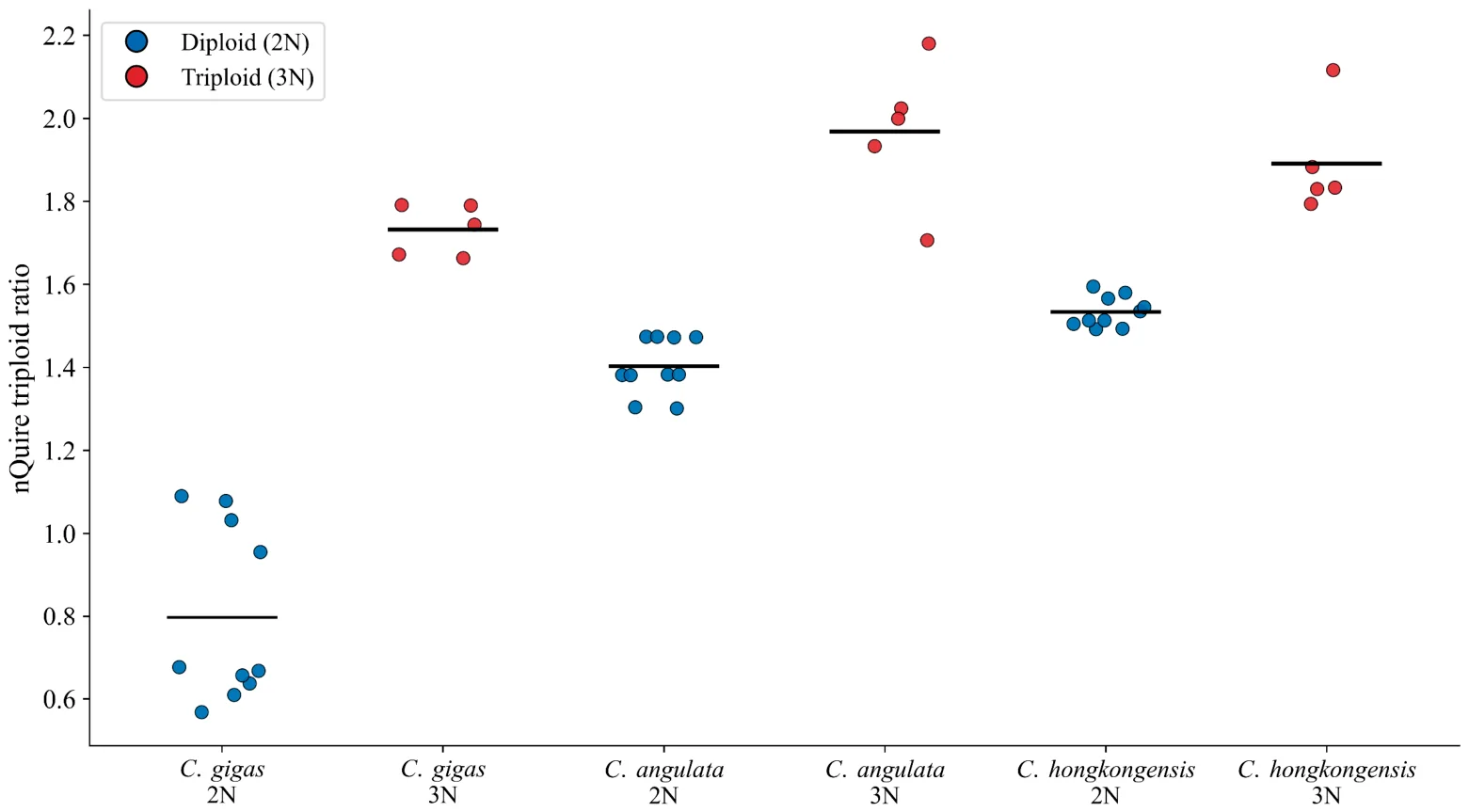
Whole-genome verification of triploidy in commercial *Crassostrea* oysters. Genome-wide allele-frequency distributions were used to distinguish diploid and triploid individuals using nQuire. The triploid-ratio statistic derived from the nQuire allele-frequency spectra is shown for species-matched diploid (2N) reference panels and commercial triploid (3N) groups of *C. gigas*, *C. angulata*, and *C. hongkongensis*. Each point represents one individual, and horizontal black bars indicate group medians. Blue and red symbols denote diploid and triploid individuals, respectively. Diploid and triploid distributions were non-overlapping within each species, allowing all 15 commercial triploid specimens to be distinguished from their corresponding species-matched diploid reference panels.

All 15 commercial triploid individuals exhibited allele-frequency spectra characteristic of triploid genomes and were clearly separated from the corresponding diploid controls (Figure 1). The triploid-ratio statistic derived from the nQuire allele-frequency spectra ranged from 1.706 to 2.180 in A3N, from 1.663 to 1.791 in G3N, and from 1.794 to 2.116 in H3N. In comparison, the corresponding diploid reference panels showed lower values, ranging from 1.301 to 1.474 in *C. angulata*, 0.568 to 1.090 in *C. gigas*, and 1.492 to 1.595 in *C. hongkongensis*. Although the absolute ratio values differed among species, the diploid and triploid distributions did not overlap within any species. Thus, ploidy could be unambiguously distinguished by species-matched comparison without requiring a common decision threshold. These results confirmed the triploid status of all individuals used for subsequent genomic and molecular analyses.

### 3.2. Cross-Species Ancestry Dosage of Triploid Groups

**Figure 2:**
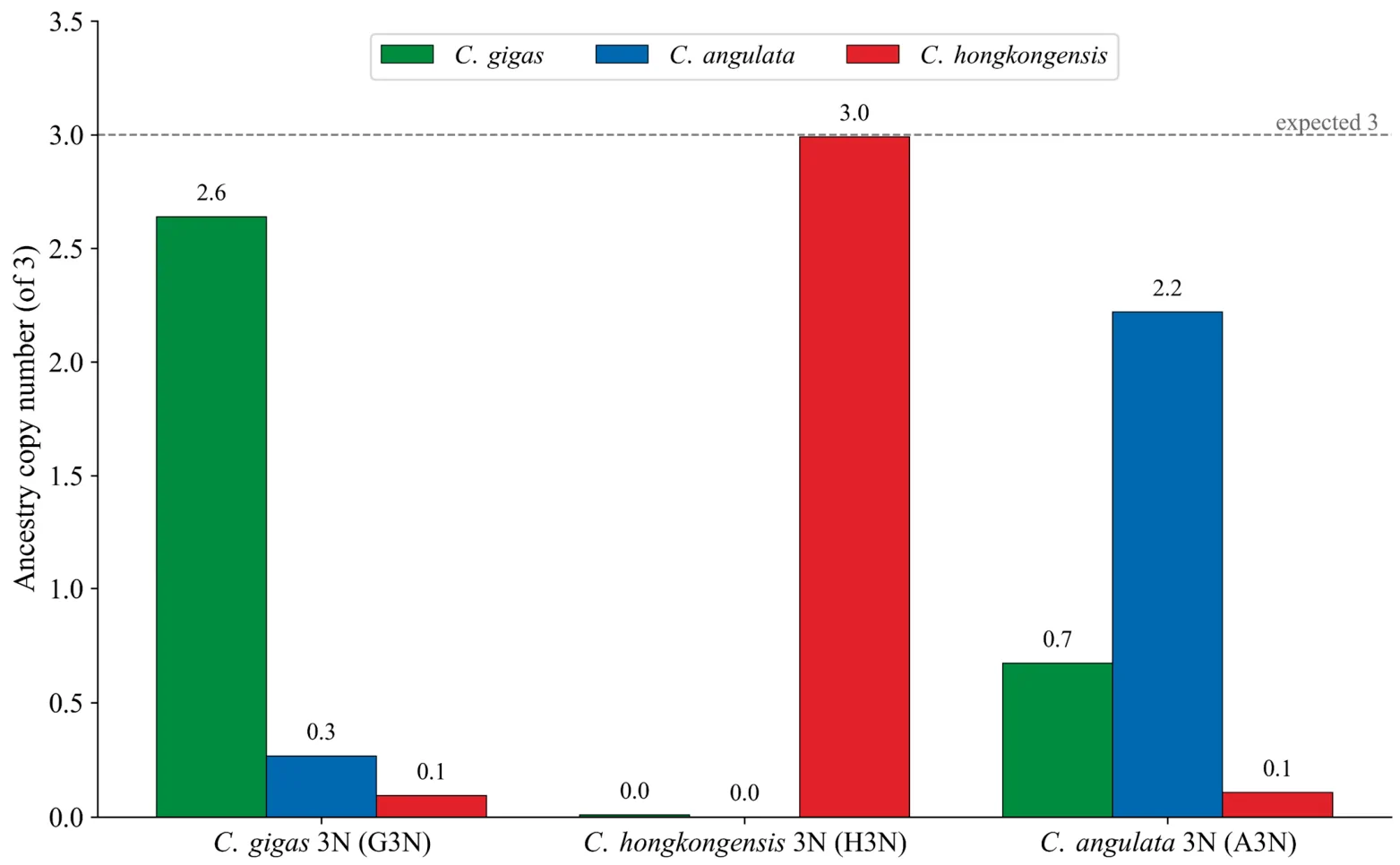
Cross-species ancestry dosage of the three triploid oyster groups. Genome-wide ancestry dosage was estimated for G3N (*Crassostrea gigas* 3N), H3N (*C. hongkongensis* 3N), and A3N (*C. angulata* 3N) using species-diagnostic nuclear variants defined from the corresponding diploid reference panels. Bias-corrected ancestry fractions were converted to haploid-equivalent copy numbers on a three-copy scale. Green, blue, and red bars represent ancestry assigned to *C. gigas*, *C. angulata*, and *C. hongkongensis*, respectively. Values above the bars indicate group-level ancestry dosage estimates, and the horizontal dashed line denotes the expected total genomic dosage of three copies in a triploid. The *C. hongkongensis* assignments are group medians; their background-control evaluation is provided in Table S12. G3N was predominantly assigned to *C. gigas* ancestry (2.6 copies), with minor *C. angulata* (0.3) and *C. hongkongensis* (0.1) signals, whereas H3N was almost exclusively assigned to *C. hongkongensis* ancestry (3.0 copies). In contrast, A3N showed mixed *C. angulata*–*C. gigas* nuclear ancestry, with approximately 2.2 and 0.7 haploid-equivalent copies assigned to these species, respectively. The minor *C. hongkongensis* assignments in A3N and G3N were interpreted as background cross-assignment rather than evidence of three-species ancestry.

Cross-species ancestry dosage analysis revealed contrasting genomic compositions among the three triploid groups (Figure 2). G3N was predominantly composed of *C. gigas*-derived ancestry, with estimated mean contributions of approximately 2.6 *C. gigas*, 0.3 *C. angulata*, and 0.1 *C. hongkongensis* haploid-equivalent copies. H3N showed the clearest single-species composition, with approximately 3.0 copies assigned to *C. hongkongensis* and negligible contributions from *C. gigas* or *C. angulata*.

In contrast, A3N displayed mixed *C. angulata*–*C. gigas* nuclear ancestry, with approximately 2.2 and 0.7 haploid-equivalent copies assigned to these species, respectively. The minor *C. hongkongensis* assignments in A3N and G3N were evaluated against diploid controls using two pairwise diagnostic-marker panels (Table S12). With the moderate-tier *C. gigas*–*C. hongkongensis* panel used for Figure 2, median *C. hongkongensis* assignments were 0.107 copies in A3N and 0.093 copies in G3N, compared with 0.167 three-copy-equivalent copies in the diploid *C. angulata* controls, which were not used to ascertain this panel. With the alternative *C. angulata*–*C. hongkongensis* panel, the corresponding estimates were 0.060, 0.121, and 0.152 copies for A3N, G3N, and the non-ascertainment diploid *C. gigas* controls, respectively.

### 3.3. Genome-Wide Ancestry Heterogeneity in A3N Triploids

**Figure 3:**
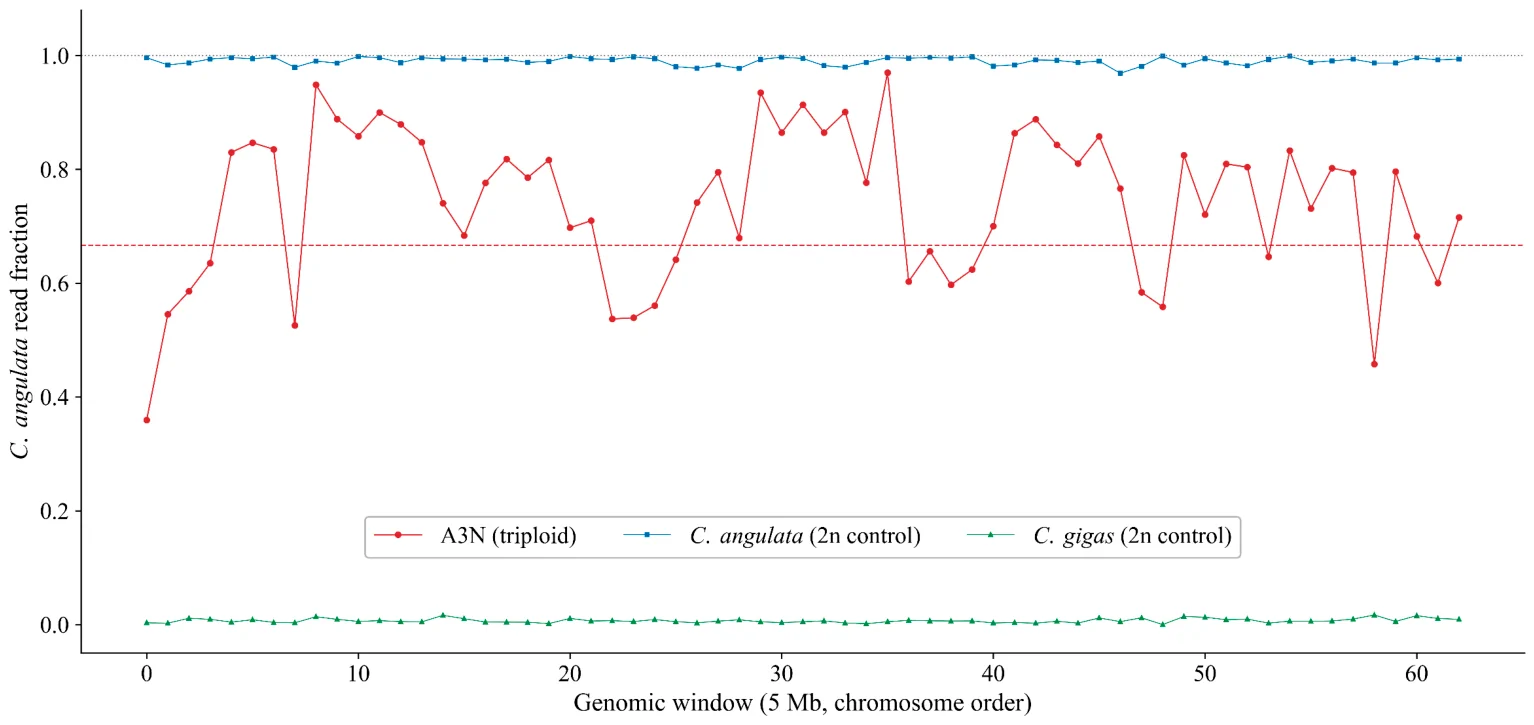
Genome-wide heterogeneity in A3N triploid oysters. Window-wise nuclear ancestry across the ten *C. angulata* reference chromosomes. Species-diagnostic loci were aggregated into non-overlapping 5 Mb windows, and the depth-weighted *C. angulata*-derived read fraction was calculated for A3N together with diploid *C. angulata* and *C. gigas* reference panels. Windows containing fewer than ten diagnostic sites were excluded. The horizontal dashed line at 2/3 represents the *C. angulata* fraction corresponding to one *C. gigas*-derived and two *C. angulata*-derived copies in a triploid genome and is shown as a dosage reference rather than as a classification threshold. The pure-species controls remained near their corresponding ancestry extremes, whereas A3N showed pronounced variation among genomic windows.

Species-diagnostic nuclear markers revealed substantial *C. gigas*–*C. angulata* ancestry heterogeneity across the A3N genomes (Figure 3). Diagnostic markers were unevenly distributed across genomic windows, with a coefficient of variation of 1.42 and a Gini coefficient of 0.67 for the moderate panel (Table S6). Across 5 Mb genomic windows, the mean *C. angulata*-derived read fraction in A3N was 0.762, corresponding to an average contribution of 2.28 *C. angulata* haploid-equivalent copies when expressed on a three-copy scale. However, window-level ancestry values showed considerably greater dispersion than expected from read-sampling variance alone. The observed standard deviation among windows was 0.140, producing an overdispersion ratio of 17.24 relative to the binomial expectation.

The ancestry variation was highly reproducible within individuals. Independent ancestry profiles calculated from odd- and even-ranked diagnostic sites were strongly correlated (split-half reliability, r = 0.989). In contrast, the mean pairwise correlation of window profiles among different A3N individuals was only r = 0.246. Thus, the genomic heterogeneity could be reproduced from independent marker subsets within the same individual but was not shared uniformly among individuals. This pattern indicates individual-specific ancestry structure rather than stochastic read-depth variation or a common mapping artefact.

### 3.4. Chromosome-Scale Local Ancestry in A3N Triploids

**Figure 4:**
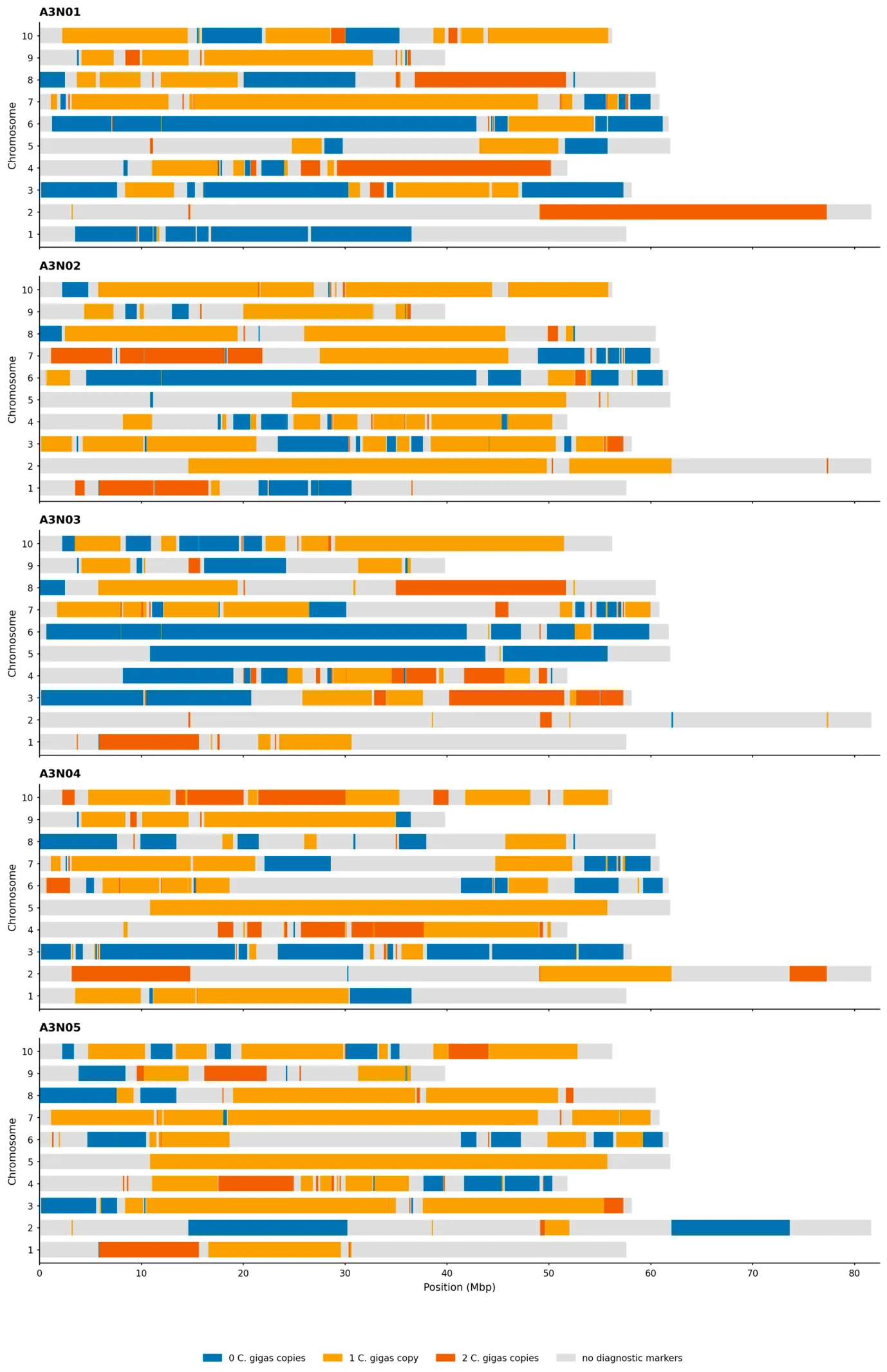
Chromosome-scale local ancestry mosaics in A3N triploid oysters. Local ancestry across the ten *C. angulata* reference chromosomes was inferred independently for A3N01–A3N05 using a three-state hidden Markov model. Blue, yellow, and red segments indicate genomic regions assigned to zero, one, and two *C. gigas*-derived copies, respectively. Grey regions indicate intervals lacking sufficient diagnostic-marker information for ancestry assignment. Chromosomes are displayed separately for each individual according to physical position in the *C. angulata* reference assembly. Marker-bracketed transition intervals and the supporting marker counts of adjacent segments are provided in Table S9; these intervals do not represent precisely resolved recombination breakpoints. Extended ancestry tracts spanning multiple megabases (Mbp) and marked differences in their chromosomal positions among individuals demonstrate chromosome-scale, individual-specific mosaic ancestry in A3N triploids.

The pronounced window-level ancestry heterogeneity in A3N was further examined by reconstructing local ancestry along each of the ten chromosomes. Hidden Markov modelling resolved the diagnostic-site data into extended genomic regions containing zero, one, or two *C. gigas*-derived copies (Figure 4). Rather than exhibiting a uniform ancestry dosage across the genome, all five A3N individuals showed mosaics of distinct ancestry states distributed across multiple chromosomes.

The chromosomal arrangement of ancestry states differed markedly among individuals. Large regions assigned to the zero-copy state were interspersed with regions containing one or two *C. gigas* copies, and several ancestry-state transitions occurred within individual chromosomes. Some chromosomes were dominated by a single ancestry state over tens of megabases, whereas others contained multiple transitions among the zero-, one-, and two-copy states. Importantly, the locations of these transitions were not shared consistently among A3N01–A3N05, consistent with the low inter-individual correlation observed in the window-based analysis.

Across individuals, 51.7–77.9% of the diagnostically informative genome contained at least one *C. gigas*-derived copy. Within the HMM-assigned segments, the copy-weighted *C. gigas* ancestry fraction ranged from 0.231 to 0.294, corresponding to approximately 0.69–0.88 haploid-equivalent copies per individual. Individual-level diagnostic-marker, HMM, and k-mer ancestry estimates are provided in Table S8. Extended *C. gigas*-containing tracts were observed in every A3N individual, with maximum tract lengths ranging from 22.48 to 44.88 Mb.

These chromosome-scale patterns demonstrate that the interspecific ancestry signal was not distributed as a uniform fractional contribution throughout the genome. Instead, *C. gigas* ancestry occurred in long, spatially structured tracts whose positions and extents differed among A3N individuals, producing individual-specific mosaic nuclear genomes.

### 3.5. Discrete Dosage Structure and Maternal-Lineage Consistency

**Figure 5:**
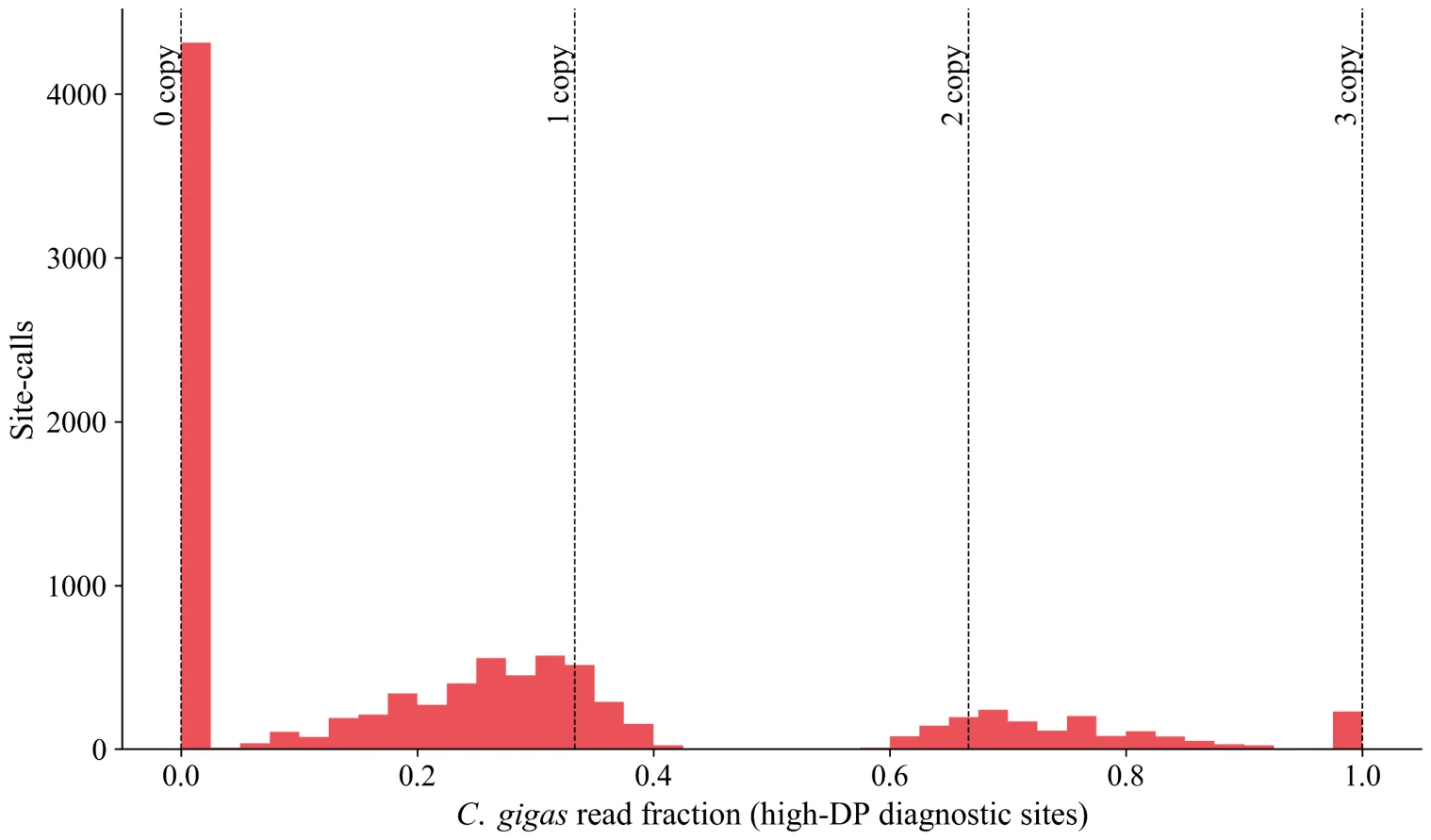
Distribution of *Crassostrea gigas* read fractions at high-confidence diagnostic sites in A3N triploid oysters. The distribution of *C. gigas*-derived read fractions was examined at species-diagnostic sites with sequencing depth ≥ 20 in the A3N triploid individuals. Vertical dashed lines indicate the expected read fractions for zero, one, two, and three *C. gigas* copies in a triploid genome (0, 1/3, 2/3, and 1, respectively). Site calls were concentrated around the expected discrete triploid dosage states, with prominent distributions near 0, 1/3, and 2/3. Calls near the three-copy state were comparatively infrequent. The discrete distribution of allele fractions supports chromosome-level *C. gigas* dosage rather than a uniformly distributed low-level *C. gigas* signal expected from simple exogenous DNA contamination.

At high-confidence species-diagnostic loci, allelic read fractions were concentrated near the expected triploid dosage states rather than forming a continuous distribution of low-frequency *C. gigas* reads (Figure 5). Distinct groups of loci occurred near the expected values for zero, one, and two *C. gigas* copies, supporting a chromosomal dosage origin of the interspecific signal. This discrete structure was inconsistent with a simple model in which the *C. gigas* reads arose from a low and approximately uniform level of exogenous DNA contamination.

Because all A3N individuals possessed *C. angulata* mitochondrial haplotypes, three-copy *C. gigas* calls were examined as a test of consistency with a maternally derived *C. angulata* genomic contribution. Three-copy *C. gigas* calls accounted for 3.0% of high-confidence individual-site observations (277 of 9098), 248 distinct diagnostic loci. Their median read depth (27×) matched the genome-wide median (27×), indicating that they were not enriched in low-coverage regions. Of the 248 distinct loci assigned to the three-copy (GGG) state, 221 (89%) occurred in only a single individual, and 93 of 150 three-copy runs consisted of a single diagnostic site, with the longest three-copy run spanning only 10 sites. By contrast, the zero-, one-, and two-copy states frequently formed multi-marker runs and long genomic tracts (Figure 4). The absence of recurrent chromosome-scale three-copy *C. gigas* segments therefore indicates that these sporadic calls are unlikely to represent a genuine GGG ancestry state.

### 3.6. Complementary Genomic Evidence of C. gigas Ancestry

**Figure 6:**
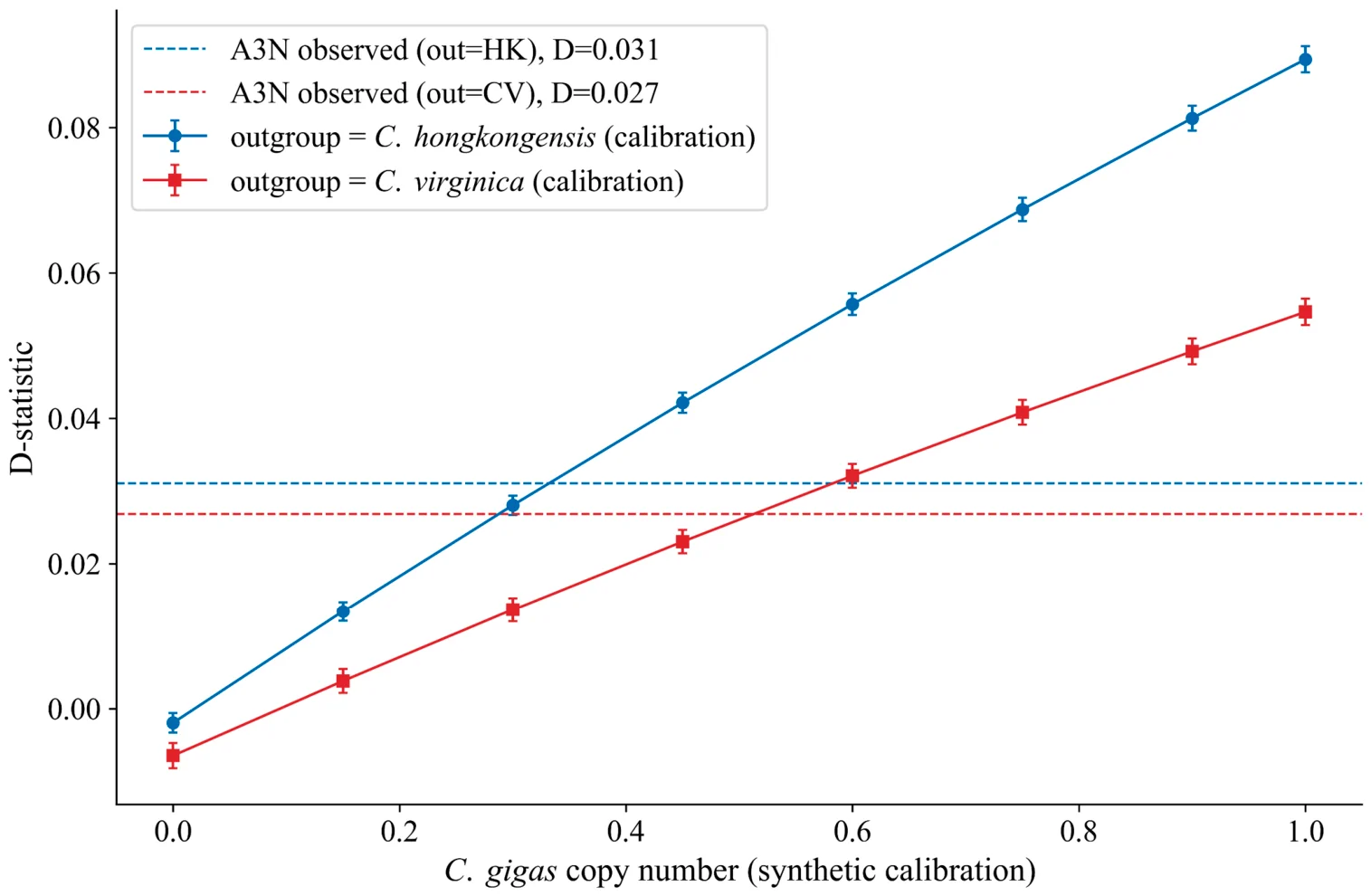
D-statistic evidence of excess *Crassostrea gigas* allele sharing in A3N triploid oysters using two alternative outgroups. Genome-wide D-statistics were calculated for the topology D(P1 = *C. angulata*, P2 = A3N, P3 = *C. gigas*, O = outgroup) using *C. hongkongensis* and *C. virginica* as independent outgroups. Blue circles and red squares represent synthetic calibration series generated with *C. hongkongensis* and *C. virginica* as outgroups, respectively. The *x*-axis indicates the simulated *C. gigas* contribution, ranging from 0 to 1 haploid-equivalent copy in a triploid genome. Error bars indicate standard errors estimated by a 5 Mb block jackknife. Horizontal dashed lines indicate the observed pooled A3N D-statistics obtained with *C. hongkongensis* (D = 0.031) and *C. virginica* (D = 0.027) as outgroups. In both calibration series, D increased monotonically with increasing simulated *C. gigas* contribution, and the observed A3N values fell within the range corresponding to partial *C. gigas* genomic contribution. Concordant positive D-statistics obtained with both outgroups support the robustness of the observed allele-sharing asymmetry to outgroup choice. The synthetic calibration was used to contextualize the magnitude of D and was not interpreted as a direct estimate of chromosome copy number.

Genome-wide ABBA–BABA analyses detected significant excess allele sharing between A3N and *C. gigas*, corroborating the ancestry estimates obtained from diagnostic markers (Figure 6). With *C. hongkongensis* as the outgroup, the pooled A3N group produced a strongly positive D-statistic (D = 0.031, SE = 0.0025, Z = 12.38). The result remained highly significant when the more distantly related *C. virginica* was used as an alternative outgroup (D = 0.027, SE = 0.0021, Z = 12.79).

The same direction of allele-sharing asymmetry was observed independently in all five A3N individuals. Using *C. hongkongensis* as the outgroup, individual D-statistics ranged from 0.0233 to 0.0358, with Z-scores from 6.61 to 11.64. With *C. virginica*, D ranged from 0.0134 to 0.0510, with Z-scores from 5.48 to 18.88. Thus, the excess sharing with *C. gigas* was not driven by a subset of individuals and was robust to outgroup choice.

Synthetic mixtures generated from diploid *C. angulata* and *C. gigas* allele frequencies produced a monotonic increase in D with increasing simulated *C. gigas* contribution for both outgroups. The observed A3N values fell between the pure *C. angulata* endpoint and the synthetic one-copy *C. gigas* calibration, consistent with an intermediate genomic contribution from *C. gigas*. Because the D-statistic measures allele-sharing asymmetry rather than chromosome dosage directly, these calibration results were used as corroborative evidence rather than as direct copy-number estimates.

**Figure 7:**
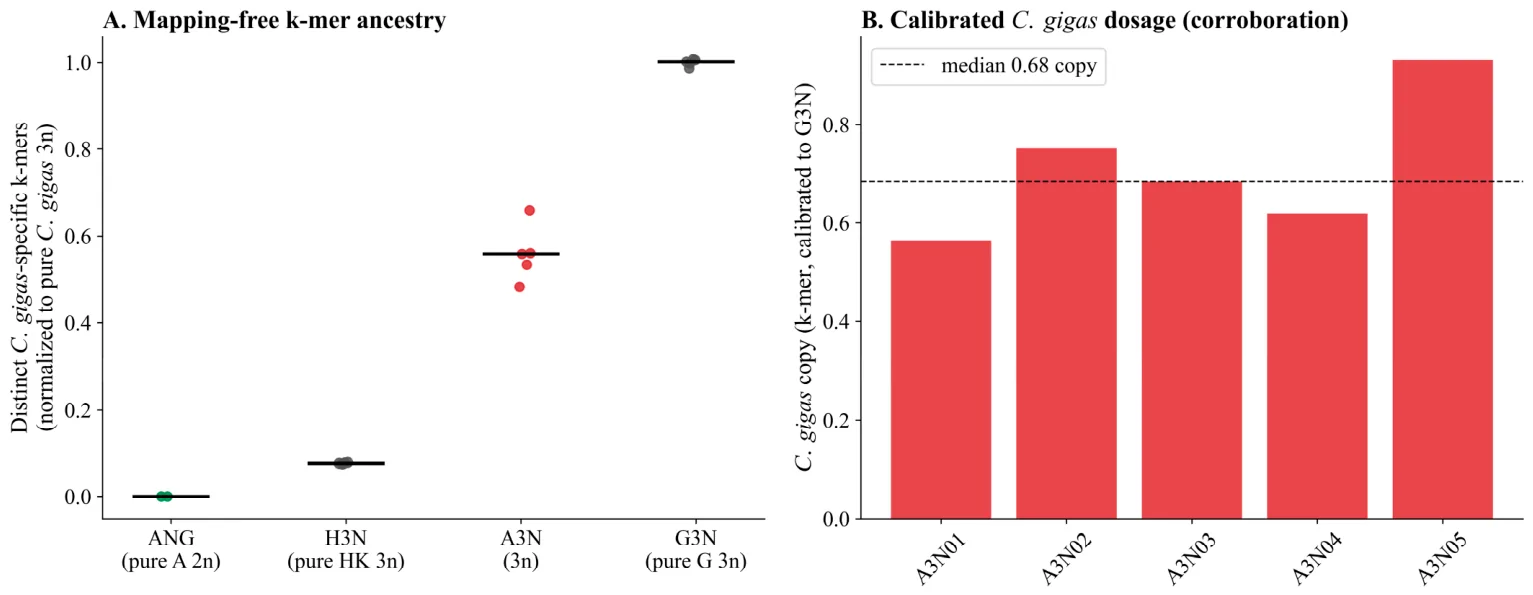
Mapping-independent k-mer evidence of *C. gigas* ancestry in A3N triploid oysters. (**A**) Mapping-independent ancestry analysis based on canonical 21-mers identified directly from whole-genome sequencing reads. *C. gigas*-diagnostic k-mers were defined using the diploid reference panels and quantified in each group after removal of singleton k-mers. Values were normalized to the *C. gigas* triploid control group (G3N), which was set to 1.0. ANG represents diploid *C. angulata*, H3N represents triploid *C. hongkongensis*, A3N represents the target triploid group, and G3N represents triploid *C. gigas*. Individual values are shown as points, and horizontal black lines indicate group medians. *C. gigas*-diagnostic k-mers were essentially absent from the ANG controls, occurred at low levels in H3N, and were consistently detected at intermediate levels in all A3N individuals relative to G3N. (**B**) Depth-calibrated *C. gigas* dosage estimates for individual A3N oysters derived from the diagnostic k-mer signal relative to the G3N control group. Bars represent the estimated haploid-equivalent *C. gigas* copy number for A3N01–A3N05. The horizontal dashed line indicates the median estimate of 0.68 *C. gigas* copies across the five A3N individuals. The calibrated estimates ranged from approximately 0.56 to 0.93 copies, providing a mapping-independent corroboration of the partial *C. gigas* nuclear genomic contribution detected by the reference-based ancestry analyses.

Analysis of canonical 21-mers provided mapping-independent corroboration of the nuclear ancestry results (Figure 7). *C. gigas*-diagnostic k-mers defined relative to *C. angulata* were absent from the diploid *C. angulata* negative controls but were detected in all five A3N individuals. The number of distinct *C. gigas*-diagnostic k-mers in A3N corresponded to 48.4–65.9% of the level observed in the G3N control panel.

After depth-based calibration against G3N, the estimated *C. gigas* contribution ranged from 0.565 to 0.931 haploid-equivalent copies among the five A3N individuals, with a median of approximately 0.68 copies. G3N individuals calibrated close to the expected three-copy state, whereas the *C. angulata* negative controls yielded no detectable *C. gigas*-diagnostic signal.

The k-mer estimates broadly overlapped the 0.69–0.88-copy range inferred from HMM-based local ancestry. Although the two methods quantify different properties of the sequence data and are not expected to yield identical estimates, their convergence supports a *C. gigas* nuclear ancestry component that is detectable without read alignment or reference-coordinate-based inference.

### 3.7. COI Region and Primer Design

**Figure 8:**
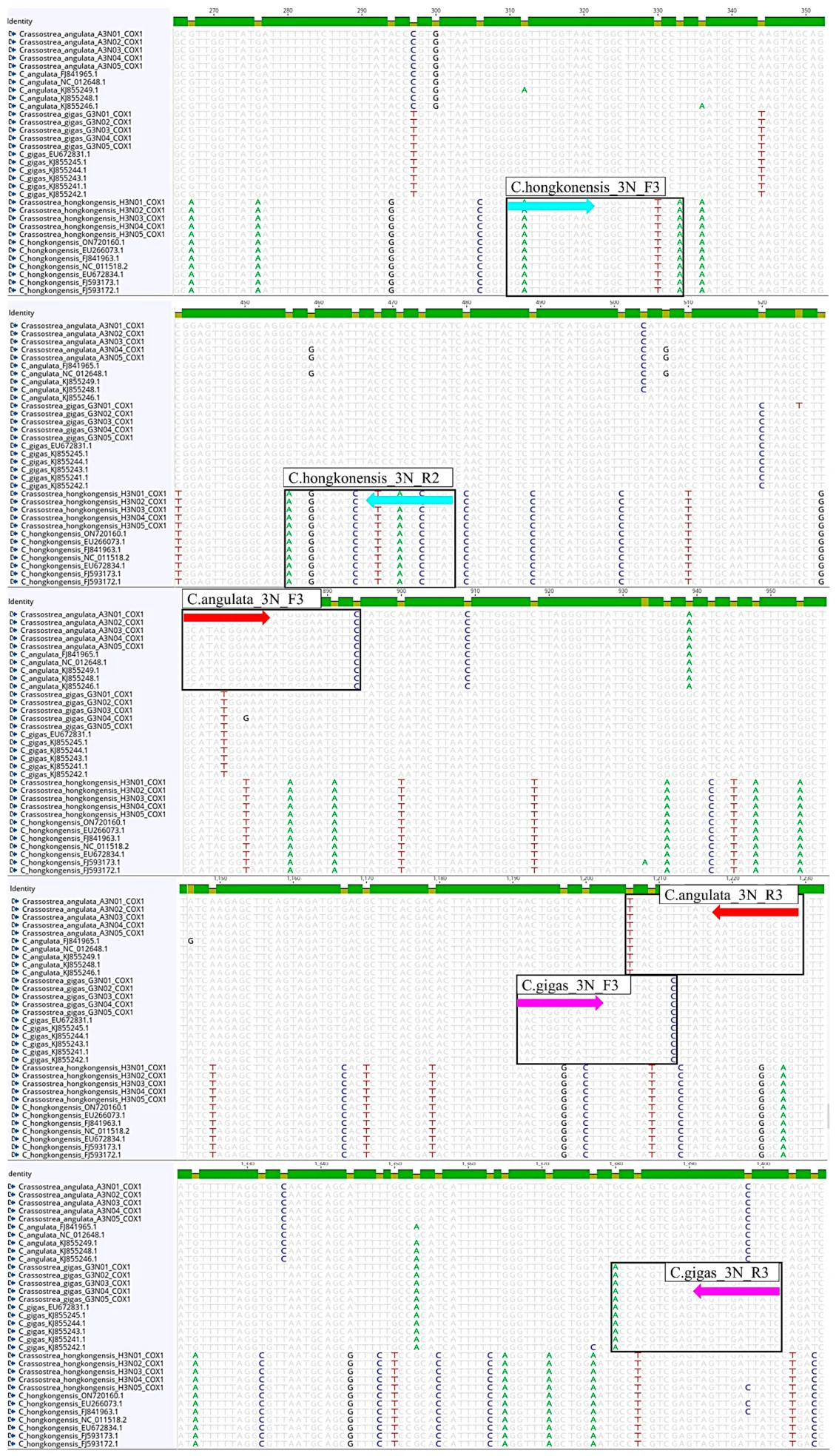
Design of species-specific COI primers based on a multiple sequence alignment of *Crassostrea gigas*, *C. angulata*, and *C. hongkongensis*. Boxes and arrows indicate the binding positions and orientations of primer pairs specific to *C. hongkongensis* (cyan), *C. angulata* (red), and *C. gigas* (magenta). Only nucleotides differing from the consensus are displayed, and each primer spans diagnostic substitutions unique to its target species.

Alignment of COI sequences from the fifteen triploid individuals examined in this study together with eighteen reference sequences retrieved from GenBank revealed species-diagnostic nucleotide substitutions suitable for primer design (Figure 8). Sequence differentiation was greater between *C. hongkongensis* and the other two species, whereas fewer diagnostic substitutions were observed between *C. gigas* and *C. angulata*.

Three species-specific primer pairs were designed from these diagnostic regions, with one forward–reverse primer pair assigned to each target species. Diagnostic substitutions were incorporated into primer-binding regions, including positions near the 3′ termini where sequence variation permitted. Reference-based primer-binding coordinates, discriminatory nucleotide positions, and their distances from the primer 3′ termini are provided in Table S10. The resulting primer pairs generated predicted amplicon sizes of 169 bp for *C. hongkongensis*, 359 bp for *C. angulata*, and 212 bp for *C. gigas* (Table 1).

### 3.8. Species Specificity of the Multiplex PCR Assay

**Figure 9:**
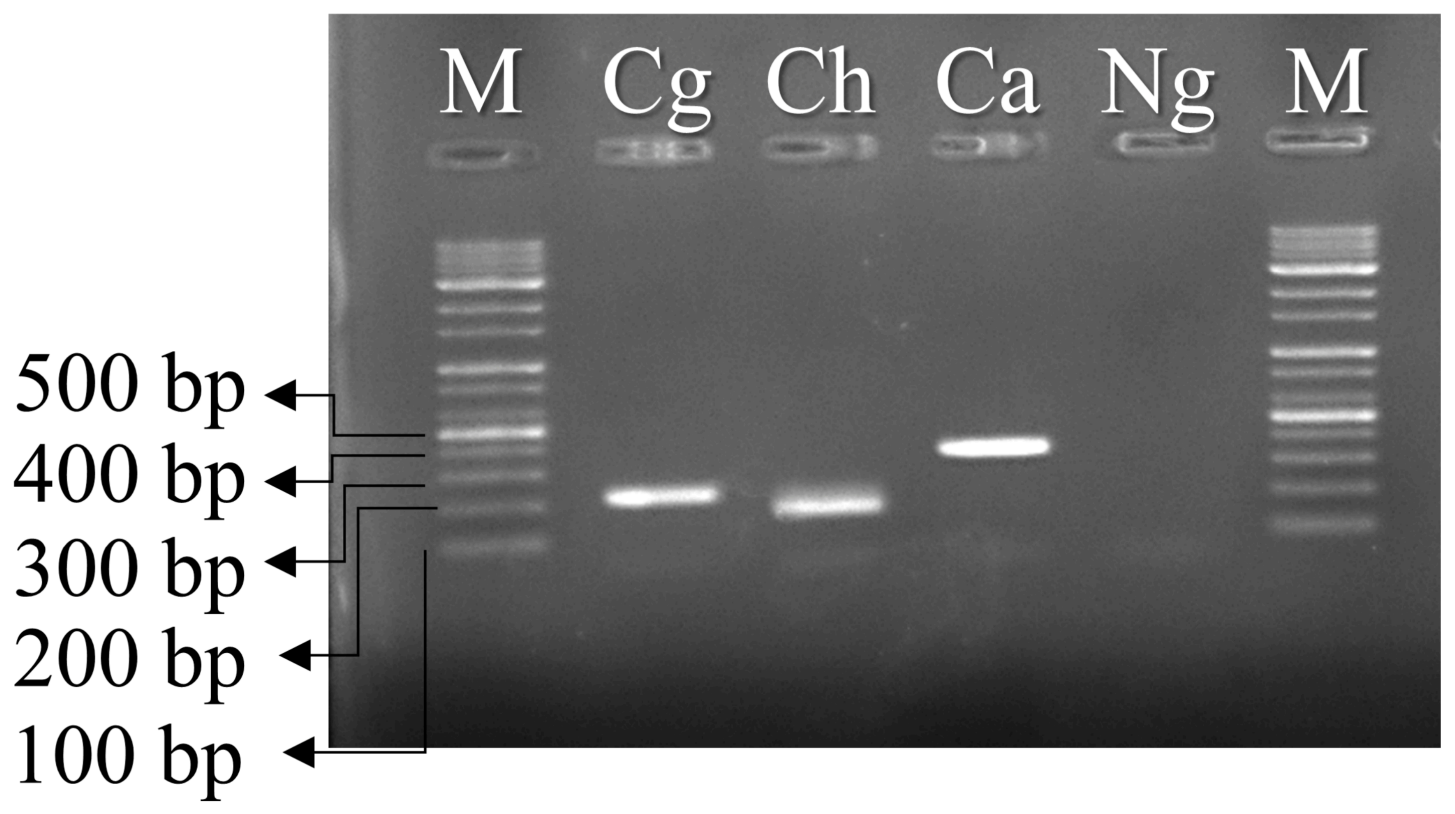
Species-specific amplification of the multiplex PCR assay for *Crassostrea gigas*, *C. hongkongensis*, and *C. angulata*. Multiplex PCR products amplified from genomic DNA of each target species and resolved on a 1.5% agarose gel. Lane M, 100 bp DNA ladder; lane Cg, *C. gigas*; lane Ch, *C. hongkongensis*; lane Ca, *C. angulata*; lane Ng, negative control containing nuclease-free water in place of template DNA. Each species yielded a single band of the expected size, and no non-specific or cross-species products were observed. The absence of amplification in the negative control indicated no detectable contamination in the assay run. Positions of the 100–500 bp ladder fragments are indicated on the left.

Under the optimized multiplex PCR conditions, each target species produced a single amplicon of the expected size (Figure 9). *Crassostrea gigas*, *C. hongkongensis*, and *C. angulata* yielded products of 212, 169, and 359 bp, respectively. No detectable cross-amplification was observed among the three species, and no amplification was detected in the no-template control.

### 3.9. Optimization of Annealing Stringency

**Figure 10:**
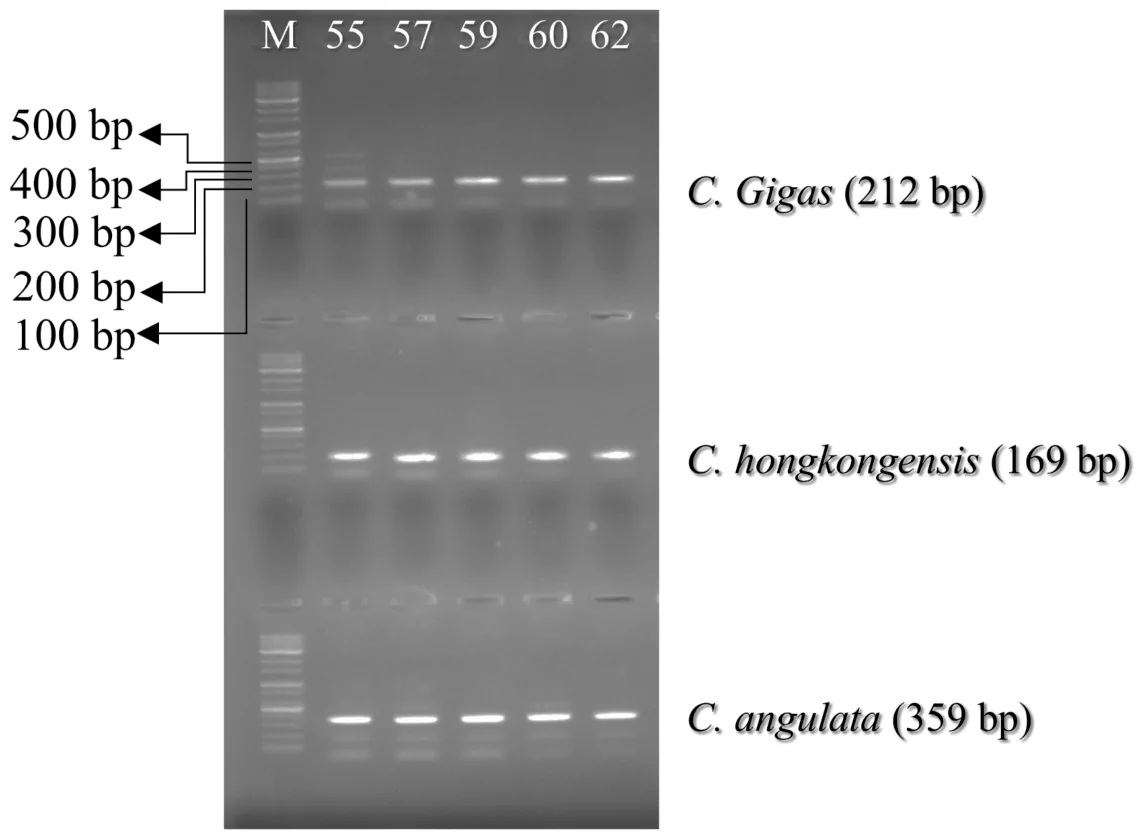
Effect of annealing temperature on species-specific amplification. Genomic DNA of *Crassostrea gigas* (212 bp), *C. hongkongensis* (169 bp), and *C. angulata* (359 bp) was amplified at 55, 57, 59, 60, and 62 °C and resolved on a 1.5% agarose gel. M, 100 bp DNA ladder; selected ladder bands are labelled with their fragment sizes in base pairs. Target bands of the expected sizes were obtained at all temperatures tested; however, faint non-specific products were observed at 60 °C and below. At 62 °C, each species yielded a single diagnostic band without detectable secondary amplification.

All three species-specific primer pairs produced the expected target amplicons across the tested annealing-temperature range of 55–62 °C (Figure 10). However, faint non-specific amplification products were observed at 60 °C and below, with secondary amplification becoming more evident as the annealing temperature decreased. At 62 °C, each species produced a single band of the expected size without detectable secondary products. An annealing temperature of 62 °C was therefore selected for subsequent multiplex PCR assays.

### 3.10. Sensitivity Across Template DNA Inputs

**Figure 11:**
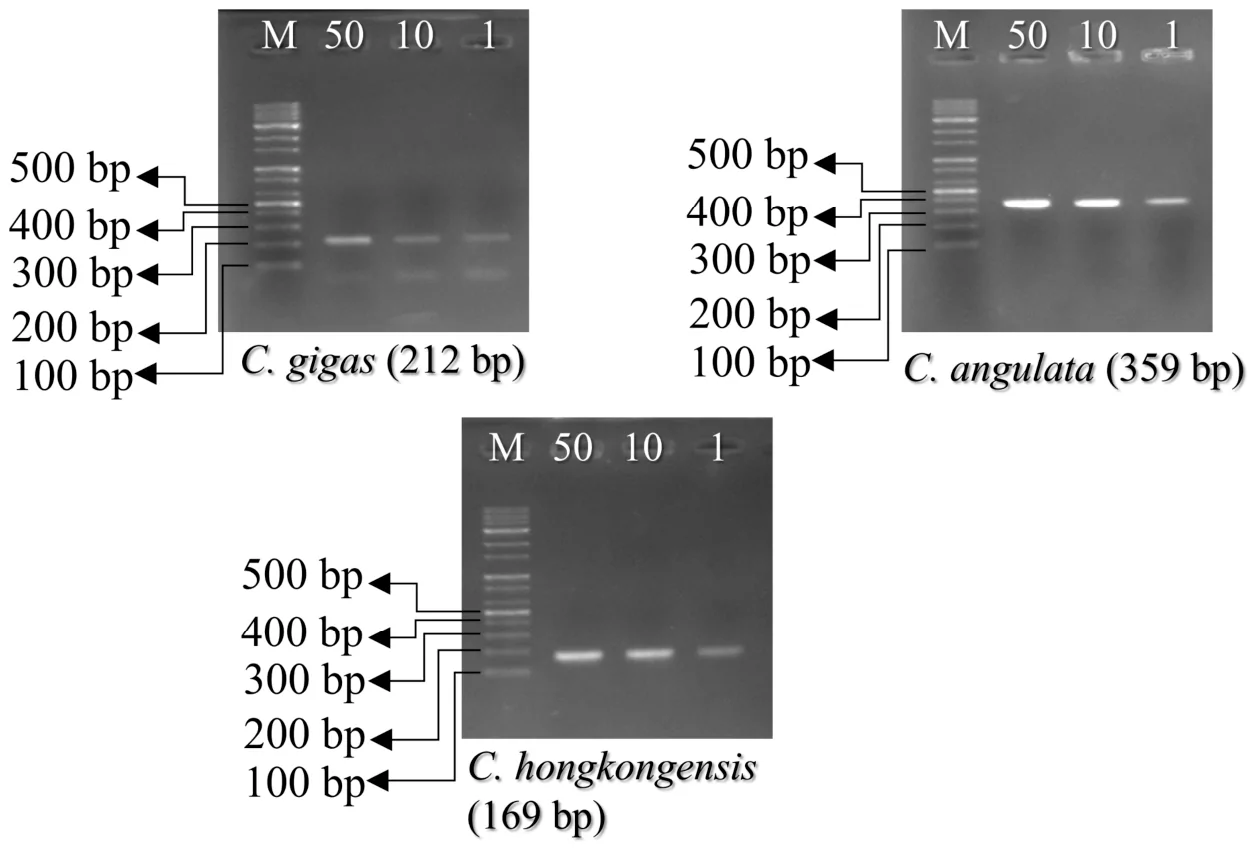
Amplification performance at varying template DNA inputs (50, 10, and 1 ng per reaction) for *Crassostrea gigas* (212 bp), *C. angulata* (359 bp), and *C. hongkongensis* (169 bp). M, 100 bp DNA ladder; selected ladder bands are labelled with their fragment sizes in base pairs. PCR reactions were performed in triplicate for each target lineage at each template input. Expected-size products were detected in all three replicates, including at 1 ng per reaction, the lowest input tested. Representative amplification results are shown.

Expected-size products were detected in all three PCR replicates at each template input of 50, 10, and 1 ng per reaction (Figure 11). *Crassostrea gigas*, *C. angulata*, and *C. hongkongensis* yielded the expected 212 bp, 359 bp, and 169 bp products, respectively. At the lowest input tested, 1 ng per reaction, all three replicates were positive for each target lineage. These results demonstrate repeatable detection under the tested conditions but do not establish a statistically defined limit of detection.

### 3.11. Validation Using Aquaculture and Commercial Oyster Specimens

**Figure 12:**
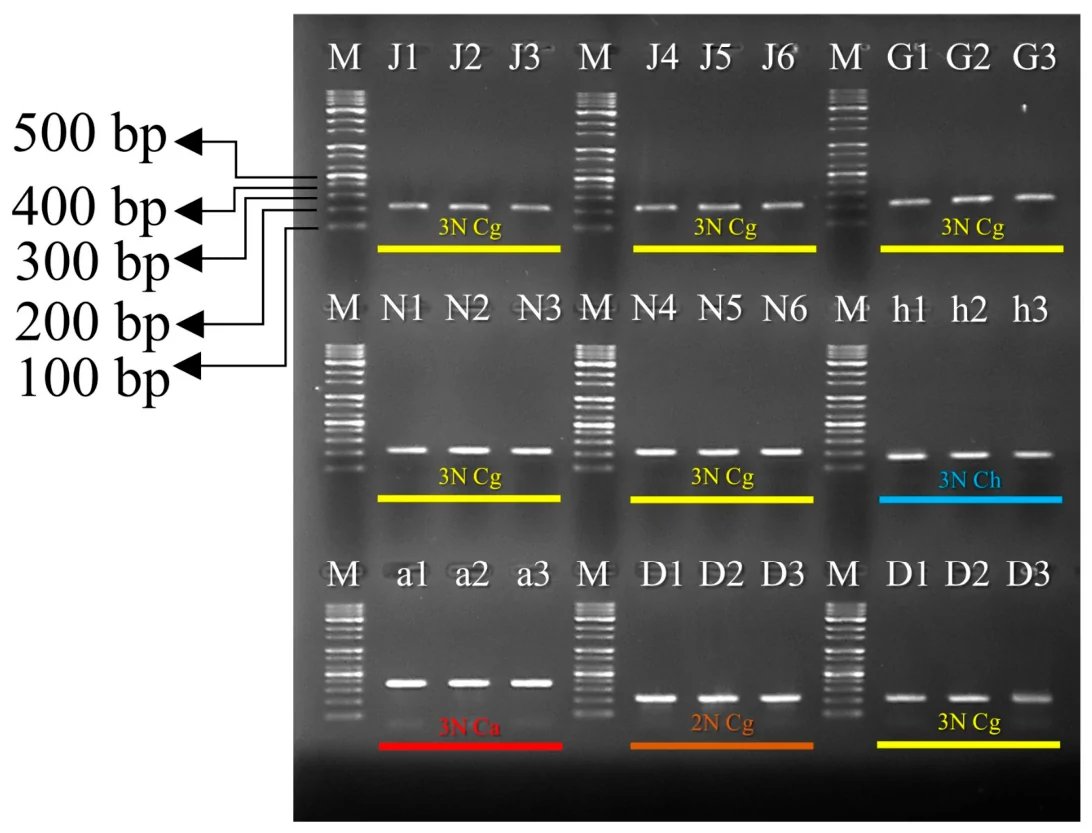
Application of the species-specific PCR assay to commercial oyster specimens. M, 100 bp DNA ladder; J1–J3 and J4–J6, Crassostrea gigas from cage and offshore cage culture in Jindo, respectively; G1–G3, *C. gigas* from Goheung; N1–N3 and N4–N6, *C. gigas* from Seolcheon and Dolseom, Namhae, respectively; h1–h3, C. hongkongensis; a1–a3, C. *angulata*; D1–D3, diploid and triploid *C. gigas* from Dalian, China. Colored bars denote the mitochondrial lineage assigned from amplicon size and indicate specimen ploidy (yellow, triploid *C. gigas*; orange, diploid *C. gigas*; cyan, triploid *C. hongkongensis*; red, triploid *C. angulata*). All specimens yielded a single diagnostic band with no cross-amplification, and diploid and triploid *C. gigas* yielded the same species-specific banding pattern.

The optimized multiplex PCR assay was applied to oyster specimens obtained from five production sites in South Korea and from Dalian, China (Figure 12). All specimens generated a single amplicon of the expected size for their COI-defined mitochondrial lineage, with no detectable cross-amplification.

Triploid *C. gigas* specimens collected from Jindo cage culture, Jindo offshore cage culture, Goheung, Seolcheon, and Dolseom produced the expected 212 bp *C. gigas*-specific amplicon. Triploid *C. hongkongensis* and A3N specimens produced the expected 169 bp and 359 bp products, respectively. The *C. gigas* specimens from Dalian also produced the 212 bp diagnostic product in both diploid and triploid individuals. Across all specimens examined, mitochondrial-lineage assignments based on multiplex PCR product size were concordant with the prior COI-based assignments.

## 4. Discussion

### 4.1. Observed Nuclear Ancestry

The present study combined whole-genome analyses and a COI-based multiplex PCR assay to characterize nuclear ancestry and identify mitochondrial lineages, respectively, in commercially produced triploid oysters. All specimens designated as triploid were unambiguously distinguished from their species-matched diploid reference panels by genome-wide allele-frequency distributions, confirming their triploid status before subsequent ancestry analyses. More importantly, the A3N oysters displayed a nuclear genomic composition that was substantially more complex than would be inferred from their nominal species designation alone.

Cross-species ancestry dosage distinguished the predominantly single-species assignments of H3N and G3N from the mixed *C. angulata*–*C. gigas* ancestry of A3N. The approximately 0.1-copy *C. hongkongensis* assignments in A3N and G3N were comparable to or below the background assignments observed in diploid controls not used for marker ascertainment and were sensitive to marker-panel selection and filtering stringency (Table S12). These findings support background cross-assignment rather than a third ancestral contribution. Possible sources include population variation not represented by the finite reference panels, shared ancestral polymorphism, and residual paralogous alignment or mapping error; their relative contributions were not resolved here. Because the same diploid individuals contributed to marker ascertainment and control evaluation, low assignments within the ascertainment panels are not independent validation. Accordingly, these results characterize panel-dependent background behaviour rather than establish a universal analytical detection limit. In contrast, the *C. gigas* ancestry signal in A3N was supported by chromosome-scale tracts, discrete dosage-state patterns, D-statistics, and mapping-independent k-mer analysis.

The whole-genome data did not support a uniform 2:1 *C. gigas*–*C. angulata* genomic composition. Cross-species dosage analysis indicated mixed *C. angulata*–*C. gigas* ancestry in A3N, whereas G3N and H3N were predominantly assigned to their nominal species. The *C. gigas* contribution in A3N exceeded the minor non-target ancestry signals observed in the predominantly single-species groups and was supported by convergent evidence from chromosome-scale, D-statistic, and mapping-independent k-mer analyses. Consistent with this result, A3N individuals showed a mean *C. angulata*-derived read fraction of 0.762, while local-ancestry reconstruction estimated approximately 0.69–0.88 haploid-equivalent *C. gigas* genome copies per individual. Importantly, the *C. gigas* contribution was not distributed as a uniform two-copy paternal component but occurred in long chromosome-scale tracts containing zero, one, or two *C. gigas*-derived copies. The locations and extents of these tracts differed substantially among individuals, revealing mosaic and individual-specific nuclear ancestry. Thus, although the A3N stock retained a *C. angulata* maternal lineage, its nuclear genomic composition was inconsistent with the simple GGA configuration expected from genetically homogeneous tetraploid *C. gigas* and diploid *C. angulata* parents.

### 4.2. Corroboration of the Ancestry Signal

Several complementary analyses support the interpretation that the *C. gigas* signal reflects nuclear ancestry rather than solely mapping or read-sampling artefacts. High-depth diagnostic loci clustered around discrete triploid dosage states; genome-wide D-statistics showed significant excess allele sharing between A3N and *C. gigas* with both *C. hongkongensis* and *C. virginica* as outgroups, and mapping-independent 21-mer analysis detected *C. gigas*-diagnostic sequence content relative to *C. angulata* in all A3N individuals. These approaches examine different properties of the sequence data but share the same biological specimens and WGS datasets; their agreement therefore constitutes complementary analytical corroboration rather than independent experimental replication. This convergence extends earlier studies of *C. gigas*–*C. angulata* allotriploids, which primarily examined growth, survival, reproductive traits, or population-level genetic diversity rather than chromosome-scale ancestry composition [2,4,5,6]. Qin et al. [2] compared production traits among reciprocal allotriploid and autotriploid crosses; Jiang et al. [6] evaluated genetically improved allotriploids derived from allotetraploid backgrounds, and Ma et al. [4] assessed genetic diversity and population structure using microsatellite markers. The present whole-genome results therefore extend these studies by documenting extensive within-genome variation in ancestry dosage and long chromosome-scale mosaic ancestry tracts in the sampled A3N individuals, features that are not apparent from ploidy or mitochondrial-lineage identification alone.

The mitochondrial and nuclear results further constrain the interpretation of the A3N ancestry. All A3N individuals carried *C. angulata* mitochondrial haplotypes, consistent with a *C. angulata* maternal lineage. Mitochondrial DNA has been shown to be maternally inherited in *C. gigas* [11]. Under a model in which the *C. angulata* mitochondrial lineage is accompanied by retention of a maternally derived *C. angulata* nuclear chromosome copy, genuine chromosome-scale regions containing three *C. gigas* copies would be unexpected. The observed three-copy calls were rare and occurred predominantly as isolated, non-recurrent sites rather than extended tracts, whereas the zero-, one-, and two-copy states formed long genomic segments. Interestingly, the greater affinity of previously produced *C. gigas*–*C. angulata* allotriploids to the female *C. angulata* population reported by Ma et al. [4] is directionally consistent with the strong *C. angulata* genomic component observed here, although microsatellite-based population affinity should not be interpreted as equivalent to chromosome-copy dosage.

### 4.3. Possible Breeding Mechanisms

Commercial triploid oysters are commonly produced by crossing tetraploid males with diploid females, a strategy that efficiently generates triploid progeny and is widely used in oyster aquaculture [27,28]. In *C. gigas* and *C. angulata*, Qin et al. [2] directly compared autotriploid and reciprocal allotriploid crosses produced from tetraploid males and diploid females, including diploid *C. angulata* females crossed with tetraploid *C. gigas* males. These reciprocal-cross experiments established the tetraploid-male × diploid-female scheme as a practical route for producing *C. gigas*–*C. angulata* allotriploids and demonstrated that such hybrids can exhibit distinct growth, survival, and reproductive characteristics depending on the direction of the cross [2]. More recently, Ma et al. [4] specifically characterized allotriploid populations generated from tetraploid *C. gigas* males and diploid *C. angulata* females and reported that these allotriploids were genetically more closely related to the female *C. angulata* population than to the paternal tetraploid population.

Accordingly, a cross between genetically pure tetraploid *C. gigas* males and diploid *C. angulata* females was considered a baseline model for evaluating ancestry dosage, rather than an established breeding history of the sampled A3N oysters. Under a simple model in which the paternal tetraploid is genetically pure *C. gigas* and the maternal diploid is pure *C. angulata*, the triploid offspring would be expected to inherit two paternal *C. gigas* chromosome copies and one maternal *C. angulata* copy, yielding an approximately 2:1 *C. gigas*–*C. angulata* genomic contribution. This expectation is also consistent with the nomenclature used in previous breeding studies, in which the corresponding allotriploid cross has been represented as GGA or DATG, reflecting two *C. gigas*-derived chromosome sets and one *C. angulata*-derived set [2,6].

Several breeding histories could account for the observed ancestry mosaics, but the available offspring data do not distinguish among them. One possibility is that the tetraploid paternal broodstock itself carried admixed or recombinant *C. gigas*–*C. angulata* ancestry rather than representing a genetically homogeneous *C. gigas* lineage. This possibility is biologically plausible because reciprocal tetraploid hybrids between *C. gigas* and *C. angulata* have already been experimentally produced, shown to survive to reproductive stages, and reported to generate functional gametes [5]. Furthermore, genetically improved allotriploid lines have recently been generated from allotetraploid broodstock containing both *C. gigas* and *C. angulata* genomic components, demonstrating that tetraploid broodstock used for triploid production need not consist exclusively of a single-species autotetraploid genome [6]. Alternatively, the breeding history of the commercial stock may have involved undocumented hybridization or introgression before triploid production. Because the actual parental broodstock used to generate the A3N individuals was unavailable for sequencing, these alternative histories cannot be distinguished directly in the present study.

The individual-specific chromosome paintings are consistent with segregation of parental chromosomes carrying different *C. gigas*–*C. angulata* ancestry backgrounds rather than with inheritance of a single fixed interspecific dosage across all offspring. Previous studies have shown that *C. gigas* and *C. angulata* can produce viable reciprocal hybrids and that interspecific crosses have been incorporated into polyploid breeding programs [2,5,6]. The long 0G, 1G, and 2G tracts observed here are therefore compatible with a breeding history involving mosaic parental ancestry. However, because the actual tetraploid sire and diploid dam were not available for genomic analysis, the breeding events that generated the observed ancestry mosaics cannot be determined directly.

### 4.4. Implications for Commercial Production and Authentication

The mitochondrial data provide a complementary view of the A3N stock. All five A3N individuals carried *C. angulata* COI haplotypes despite an estimated *C. gigas* nuclear contribution of approximately 0.7 haploid-equivalent copies. Their classification as the *C. angulata* mitochondrial lineage by multiplex PCR therefore illustrates that COI-based lineage identification does not resolve mixed nuclear ancestry. The assay generated diagnostic products of 212, 359, and 169 bp for *C. gigas*, *C. angulata*, and *C. hongkongensis*, respectively, without detectable cross-amplification, and amplification remained detectable at template inputs as low as 1 ng. This distinction is particularly relevant for allotriploid aquaculture because reciprocal *C. gigas*–*C. angulata* hybrids may differ in parental origin and cross direction [2], whereas a mitochondrial COI assay identifies only the maternal lineage.

Previous studies have demonstrated the utility of mitochondrial markers for seafood authentication, including COI-based identification of *Crassostrea* oysters and the 291 bp NAD2 assay developed by Ceruso et al. for *Pagellus erythrinus* using endpoint and real-time PCR [9,29,30]. The present assay uses diagnostic COI substitutions, preferentially positioned near primer 3′ termini, in a three-lineage multiplex endpoint PCR format. Its specificity was evaluated among the three target lineages, and expected-size products were detected at template inputs down to 1 ng per reaction. These results extend mitochondrial-lineage identification to genomically characterized commercial triploid oysters, without establishing superior specificity or sensitivity relative to previous assays. However, the discovery of mosaic nuclear ancestry in A3N emphasizes that mitochondrial lineage authentication and nuclear ancestry characterization address different biological questions. The multiplex PCR assay supports rapid mitochondrial-lineage identification in the specimens examined, whereas genome-wide analysis is required to characterize mixed nuclear ancestry. Amplification at 1 ng represents detection at the lowest template input tested rather than a statistically established limit of detection. Although the assay amplified the tested specimens successfully, the field-validation sample was limited and did not establish performance across the geographic and genetic diversity of the target lineages. Primer-binding sites were examined in the study-derived and selected GenBank sequences, but unsampled within-species variants, particularly near the primer 3′ termini, could reduce amplification efficiency or cause false-negative results. Broader application therefore requires validation in larger, geographically diverse sample sets, together with further assessment of extraction reproducibility, mixed-species templates, additional non-target taxa, and degraded or processed samples.

### 4.5. Limitations and Future Validation

The limited A3N sample size and substantial inter-individual variation preclude treating the estimated ancestry proportions as representative of commercial stocks. Diagnostic markers were defined using limited diploid reference panels, and unsampled population variation may affect marker diagnosticity and ancestry estimates. The diagnostic 21-mer analysis provides a complementary measure of sequence content rather than a model-based estimate of genomic heterozygosity or repeat content. Repetitive sequences and copy-number variation may influence diagnostic k-mer abundance, particularly the depth-weighted estimates, and normalization to G3N does not necessarily remove sample-specific effects. Separately, paralogous alignment and allele-specific mapping may distort the allelic read fractions used for alignment-based ploidy assessment. The k-mer-derived dosages were therefore interpreted alongside the other ancestry analyses rather than as exact genome-wide ancestry proportions. The absence of genomic data from the actual parental broodstock prevents reconstruction of the specific breeding history. Broader sampling across documented production batches and geographic sources, together with parental–offspring genomic analysis, is required to establish the generality and origin of the observed ancestry mosaics.

## 5. Conclusions

Whole-genome characterization of five commercial A3N oysters revealed chromosome-scale mosaics of *C. gigas*–*C. angulata* nuclear ancestry despite a shared *C. angulata* mitochondrial lineage. Local-ancestry analysis estimated 0.69–0.88 haploid-equivalent *C. gigas* copies per individual, supported by concordant cross-species dosage, discrete dosage-state, D-statistic, and mapping-independent k-mer analyses. These findings establish heterogeneous nuclear ancestry in the sampled A3N individuals, while its prevalence and distribution across commercial stocks remain to be determined. These results are inconsistent with the uniform two-*C. gigas*-copy and one-*C. angulata*-copy configuration expected under a genetically homogeneous parental model. However, the observed mosaics do not establish a specific parental origin or crossing pathway, because the actual parental broodstock was unavailable for genomic analysis. In parallel, the COI-based multiplex PCR assay distinguished the mitochondrial lineages of *C. gigas*, *C. angulata*, and *C. hongkongensis* in the tested specimens, with expected-size products detected at template inputs down to 1 ng per reaction. Genome-wide ancestry analysis and mitochondrial PCR authentication therefore provide complementary information: the former resolves complex nuclear ancestry, whereas the latter offers a rapid and practical means of determining maternal mitochondrial lineage. Together, these approaches provide a framework for more accurate characterization, authentication, and traceability of commercial triploid oyster stocks.

## Figures and Tables

**Table 1:** COI-targeted primers for mitochondrial-lineage identification of *C. gigas*, *C. angulata*, and *C. hongkongensis*, validated in triploid specimens.

Primer Name	Target Species	Nucleotide Sequence (5′-3′)	Direction	Expected Amplicon Size (bp)
C.hongkongensis_3N_F3	*C. hongkongensis*	GGATTTGGTAACTGGCTTATTCCA	Forward	169
C.hongkongensis_3N_R2		ATAAGGGTGGATAGATTGTCCAT	Reverse	
C.angulata_3N_F3	*C. angulata*	GCATACGGAAATATGGGAATGTTC	Forward	359
C.angulata_3N_R3		ACCGCACCCATTGATAAAACGTAA	Reverse	
C.gigas_3N_F3	*C. gigas*	AACTGGTCATTTTCACTACGTC	Forward	212
C.gigas_3N_R3		GATAATCAACTACTCGACGTGGT	Reverse	

## Data Availability

The publicly available sequencing datasets used as reference panels and outgroups are listed in Table S2. The 3NdeTECT v0.2.0 workflow and source code are publicly available at https://github.com/krkim1989/3NdeTECT, accessed on 18 August 2026. The raw sequencing data generated in this study have been deposited in the NCBI Sequence Read Archive (SRA) under BioProject accession PRJNA1514805 (SRA accession: SRR40253183-SRR40253197), with BioSample accessions SAMN62522670–SAMN62522684.

## Supplementary Materials

The following supporting information can be downloaded at: https://www.mdpi.com/article/10.3390/foods15213921/s1. Table S1: Sampling sites of *Crassostrea* species in the study; Table S2: Publicly available whole-genome sequencing datasets used as diploid reference panels and outgroups; Table S3: Step-wise filtering of species-diagnostic SNPs between *Crassostrea gigas* and *C. angulata*; Table S4: Distribution of species-diagnostic markers among chromosomes; Table S5: Number of species-diagnostic markers in each 5-Mb window; Table S6: Uniformity of species-diagnostic marker density across 5-Mb windows; Table S7: Distances between adjacent moderate-tier diagnostic markers; Table S8: Individual-level nuclear ancestry estimates for A3N oysters; Table S9: Genomic coordinates and marker support of inferred ancestry transitions in A3N oysters; Table S10: COI reference coordinates and diagnostic nucle-otide positions underlying primer specificity; Table S11: D-statistics, block-jackknife standard errors, Z-scores and 95% confidence intervals for the estimates in Figure 6 and Section 3.6; Table S12: *Crassostrea hongkongensis* ancestry assigned to diploid reference panels and triploid groups by the two *C. hongkongensis* pairwise diagnostic-marker panels; Figure S1: Individual-level profiles of the *C. angulata* read fraction across 5-Mb genomic windows in A3N oysters.
