# Supplementary material for: Genomic Characterization and Multiplex PCR Authentication of Commercial Triploid Oysters: Evidence for Mosaic Crassostrea gigas–C. angulata Ancestry
Source: Foods. 2026 Nov 2;15(21):3921. doi: 10.3390/foods15213921 (PMC13762542; doi:10.3390/foods15213921)
Supplement: Supplementary file 1 [file foods-15-03921-s001.zip › foods-4548521-supplementary.pdf]

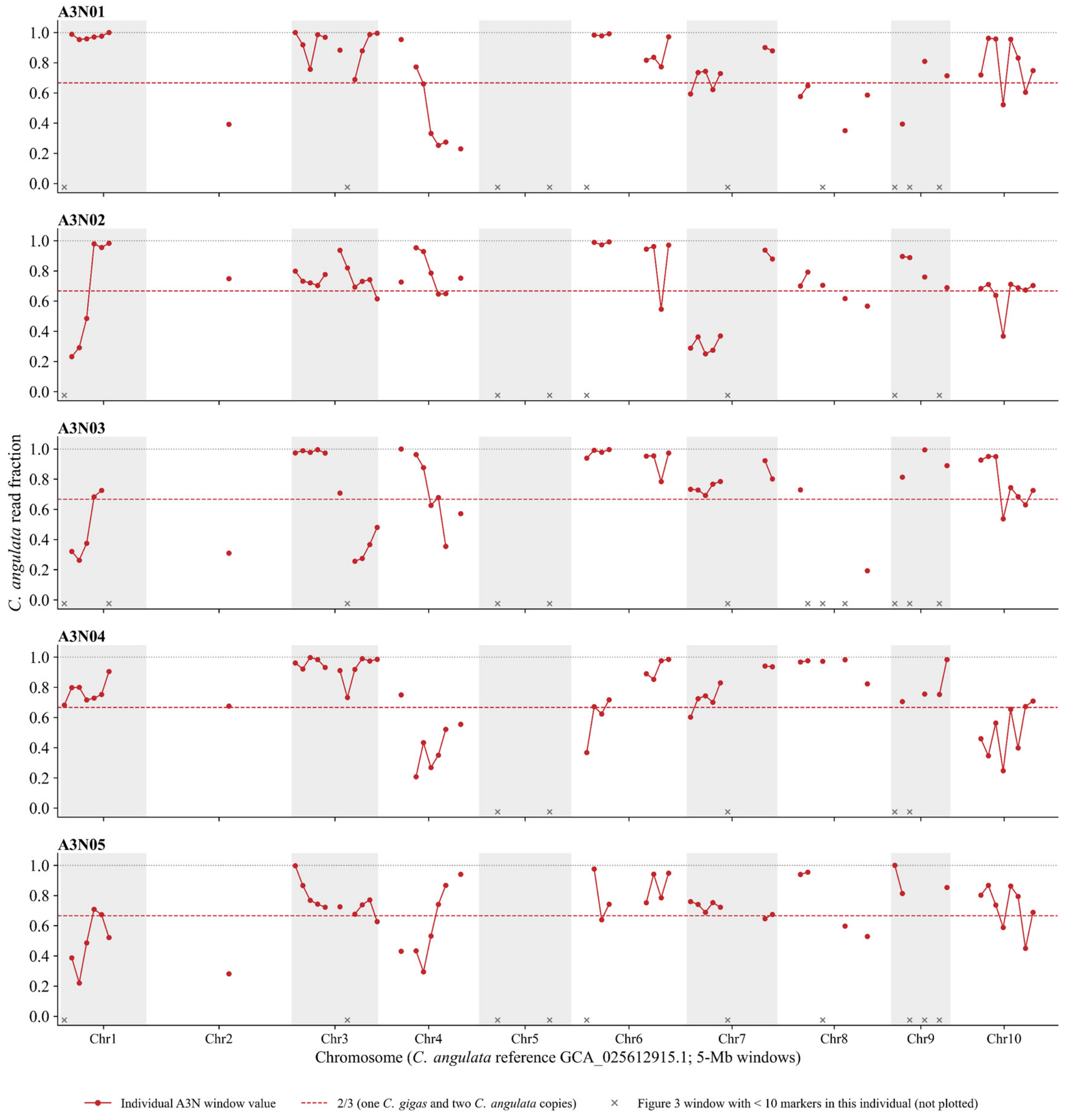
A3N01
A3N02
A3N03
A3N04
A3N05
1.0
0.8
0.6
0.4
0.2
0.0
C. angulata read fraction
Chr1
Chr2
Chr3
Chr4
Chr5
Chr6
Chr7
Chr8
Chr9
Chr10
Chromosome (C. angulata reference GCA_025612915.1; 5-Mb windows)
Individual A3N window value
2/3 (one C. gigas and two C. angulata copies)
Figure 3 window with < 10 markers in this individual (not plotted)

Figure S1. Individual-level profiles of the C. angulata read fraction across 5-Mb genomic windows in A3N oysters.

Table S1. Sampling sites of *Crasosstrea* species in the study

Species	Ploidy	Country	Sample source	Location	Location	Sample date
C. gigas	3N	South Korea	Commercial market	Namhae Seolcheon	34°56'08″ N, 127°51'39" E	2024.08
C. gigas	3N	South Korea	Commercial market	Namhae Dolseom	34°44'24″ N, 127°40'18" E	2024.08
C. gigas	3N	South Korea	Commercial market	Goheung	34°25'21″ N, 127°09'29" E	2024.08
C. gigas	3N	South Korea	Commercial market	Jindo cage	34°23'34″ N, 126°15'43" E	2024.08
C. gigas	3N	South Korea	Commercial market	Jindo offshore cage	34°23'15″ N, 126°16'16" E	2024.08
C. gigas	3N	South Korea	Hatchery	Gojaedo	34°52′50″ N, 128°37′16″ E	2024.08
C. gigas	3N	China	Commercial market	Dalian	38°56'33″ N, 121°37'19" E	2024.08
C. gigas	2N	China	Commercial market	Dalian	38°56'33″ N, 121°37'19" E	2024.08
C. angulata	3N	China	Commercial market	Shenzhen	22°32'13″ N, 114°13'28" E	2024.08
C. hongkongensis	3N	China	Commercial market	Shanghai	30°48'38″ N, 121°38'59" E	2024.08

Table S2. Publicly available whole-genome sequencing datasets used as diploid reference panels and outgroups.

Sample	Species	Role	SRA_Run
ANG01	*C. angulata*	2N reference panel	SRR31365005
ANG02	*C. angulata*	2N reference panel	SRR31365006
ANG03	*C. angulata*	2N reference panel	SRR31365007
ANG04	*C. angulata*	2N reference panel	SRR31365008
ANG05	*C. angulata*	2N reference panel	SRR31365009
ANG06	*C. angulata*	2N reference panel	SRR31365010
ANG07	*C. angulata*	2N reference panel	SRR31365011
ANG08	*C. angulata*	2N reference panel	SRR31365012
ANG09	*C. angulata*	2N reference panel	SRR31365013
ANG10	*C. angulata*	2N reference panel	SRR31365014
GIG01	*C. gigas*	2N reference panel	SRR6062976
GIG02	*C. gigas*	2N reference panel	SRR6062977
GIG03	*C. gigas*	2N reference panel	SRR6062978
GIG04	*C. gigas*	2N reference panel	SRR6062979
GIG05	*C. gigas*	2N reference panel	SRR6062980
GIG06	*C. gigas*	2N reference panel	SRR6062981
GIG07	*C. gigas*	2N reference panel	SRR6062982
GIG08	*C. gigas*	2N reference panel	SRR6062983
GIG09	*C. gigas*	2N reference panel	SRR6062984
GIG10	*C. gigas*	2N reference panel	SRR6062985
HK01	*C. hongkongensis*	2N reference panel	SRR16685763
HK02	*C. hongkongensis*	2N reference panel	SRR16685762
HK03	*C. hongkongensis*	2N reference panel	SRR16685761
HK04	*C. hongkongensis*	2N reference panel	SRR16685760
HK05	*C. hongkongensis*	2N reference panel	SRR16685759
HK06	*C. hongkongensis*	2N reference panel	SRR16685758
HK07	*C. hongkongensis*	2N reference panel	SRR16685757

HK08	*C. hongkongensis*	2N reference panel	SRR16685756
HK09	*C. hongkongensis*	2N reference panel	SRR16685755
HK10	*C. hongkongensis*	2N reference panel	SRR16685754
CV01	*C. virginica*	2N outgroup	SRR24056612
CV02	*C. virginica*	2N outgroup	SRR24056615
CV03	*C. virginica*	2N outgroup	SRR24056605
CV04	*C. virginica*	2N outgroup	SRR24056608
CV05	*C. virginica*	2N outgroup	SRR24056600
CV06	*C. virginica*	2N outgroup	SRR24056594
CV07	*C. virginica*	2N outgroup	SRR24056588
CV08	*C. virginica*	2N outgroup	SRR24056583
CV09	*C. virginica*	2N outgroup	SRR24056577
CV10	*C. virginica*	2N outgroup	SRR24056651

Table S3. Step-wise filtering of species-diagnostic SNPs between *Crassostrea gigas* and *C. angulata*.

Step	Filter	Sites retained (n)	Retained from previous step (%)	Tier
0	Biallelic SNPs with QUAL ≥ 30 in the joint callset (25 individuals)	49,065,942	—	
1	≥ 1 valid genotype (DP ≥ 8, GQ ≥ 20) in each diploid panel	36,947,696	75.30	
2	≥ 7 of 10 individuals with valid genotypes in each panel	3,465,948	9.38	
3	Opposite near-fixation between panels (pooled ALT read fraction ≤ 0.05 vs. ≥ 0.95)	3,653	0.11	
4	≥ 80% of valid individuals per panel carrying the panel-specific allele	3,623	99.18	Moderate tier
5	≥ 8 of 10 individuals with valid genotypes in each panel	965	26.64	
6	Pooled ALT read fraction ≤ 0.02 vs. ≥ 0.98	608	63.01	
7	≥ 90% of valid individuals per panel carrying the panel-specific allele	594	97.70	Strict tier

Filters were applied sequentially; each row gives the number of sites passing that filter and all preceding filters. A valid genotype required read depth (DP) ≥ 8 and genotype quality (GQ) ≥ 20. Pooled ALT read fraction = alternative-allele reads / total reads summed over valid individuals of a panel. Steps 0–4 define the moderate tier used for the window-based, local-ancestry and dosage-state analyses; steps 5–7 define the strict subset of the moderate tier. Triploid A3N individuals were not used in any filtering step.

Table S4. Distribution of species-diagnostic markers among chromosomes.

Chromosome	Length (Mb)	Moderate-tier markers (n)	Strict-tier markers (n)	Markers with C. angulata allele = REF (n)	Markers with C. angulata allele = ALT (n)	Density (moderate markers/Mb)	5-Mb windows (n)	Windows used in window analysis (n)
Chr1 (CM046776.1)	57.61	349	49	284	65	6.06	12	7
Chr2 (CM046777.1)	81.66	56	4	50	6	0.69	17	1
Chr3 (CM046778.1)	58.15	684	121	593	91	11.76	12	11
Chr4 (CM046779.1)	51.82	513	81	449	64	9.9	11	7
Chr5 (CM046780.1)	61.93	52	8	43	9	0.84	13	2
Chr6 (CM046781.1)	61.76	735	150	627	108	11.9	13	8
Chr7 (CM046782.1)	60.85	498	86	401	97	8.18	13	8
Chr8 (CM046783.1)	60.48	131	14	110	21	2.17	13	5
Chr9 (CM046784.1)	39.83	175	25	159	16	4.39	8	6
Chr10 (CM046785.1)	56.23	430	56	313	117	7.65	12	8
Total	590.31	3,623	594	3,029	594	6.14	124	63

Diploid reference panels: ten *C. gigas* and ten *C. angulata* individuals (Table S2). REF/ALT: whether the *C. angulata*-specific allele is the reference or the alternative base; both orientations were used for the reciprocal bias correction (Section 2.4). Windows used in window analysis: 5-Mb windows containing ≥ 10 diagnostic sites with A3N read data (Section 2.5.1).

Table S5. Number of species-diagnostic markers in each 5-Mb window.

Chromosome	Window start (bp)	Window end (bp)	Biallelic SNPs, QUAL ≥ 30 (n)	Moderate-tier markers (n)	Strict-tier markers (n)	Moderate markers with A3N read data (n)	Used in window analysis
Chr1 (CM046776.1)	1	5,000,000	436,643	10	0	10	Yes
Chr1 (CM046776.1)	5,000,001	10,000,000	427,119	71	4	71	Yes
Chr1 (CM046776.1)	10,000,001	15,000,000	460,077	88	11	86	Yes
Chr1 (CM046776.1)	15,000,001	20,000,000	471,736	64	10	64	Yes
Chr1 (CM046776.1)	20,000,001	25,000,000	413,805	38	16	38	Yes
Chr1 (CM046776.1)	25,000,001	30,000,000	437,922	61	5	61	Yes
Chr1 (CM046776.1)	30,000,001	35,000,000	446,176	16	3	16	Yes
Chr1 (CM046776.1)	35,000,001	40,000,000	460,676	1	0	1	No
Chr1 (CM046776.1)	40,000,001	45,000,000	383,281	0	0	0	No
Chr1 (CM046776.1)	45,000,001	50,000,000	408,520	0	0	0	No
Chr1 (CM046776.1)	50,000,001	55,000,000	360,208	0	0	0	No
Chr1 (CM046776.1)	55,000,001	57,608,718	210,398	0	0	0	No
Chr2 (CM046777.1)	1	5,000,000	328,473	1	0	1	No
Chr2 (CM046777.1)	5,000,001	10,000,000	330,035	0	0	0	No
Chr2 (CM046777.1)	10,000,001	15,000,000	413,297	4	2	4	No
Chr2 (CM046777.1)	15,000,001	20,000,000	371,866	0	0	0	No
Chr2 (CM046777.1)	20,000,001	25,000,000	461,837	0	0	0	No
Chr2 (CM046777.1)	25,000,001	30,000,000	414,921	0	0	0	No
Chr2 (CM046777.1)	30,000,001	35,000,000	442,083	1	0	1	No
Chr2 (CM046777.1)	35,000,001	40,000,000	360,538	1	0	1	No
Chr2 (CM046777.1)	40,000,001	45,000,000	438,732	0	0	0	No
Chr2 (CM046777.1)	45,000,001	50,000,000	442,205	40	2	39	Yes

Chr2 (CM046777.1)	50,000,001	55,000,000	449,673	3	0	3	No
Chr2 (CM046777.1)	55,000,001	60,000,000	364,591	0	0	0	No
Chr2 (CM046777.1)	60,000,001	65,000,000	315,438	3	0	3	No
Chr2 (CM046777.1)	65,000,001	70,000,000	307,360	0	0	0	No
Chr2 (CM046777.1)	70,000,001	75,000,000	395,786	1	0	1	No
Chr2 (CM046777.1)	75,000,001	80,000,000	439,311	2	0	1	No
Chr2 (CM046777.1)	80,000,001	81,655,936	137,677	0	0	0	No
Chr3 (CM046778.1)	1	5,000,000	389,436	33	1	33	Yes
Chr3 (CM046778.1)	5,000,001	10,000,000	401,809	52	9	52	Yes
Chr3 (CM046778.1)	10,000,001	15,000,000	437,969	151	31	149	Yes
Chr3 (CM046778.1)	15,000,001	20,000,000	439,222	81	18	81	Yes
Chr3 (CM046778.1)	20,000,001	25,000,000	464,879	20	3	20	Yes
Chr3 (CM046778.1)	25,000,001	30,000,000	329,189	4	0	4	No
Chr3 (CM046778.1)	30,000,001	35,000,000	472,958	74	8	74	Yes
Chr3 (CM046778.1)	35,000,001	40,000,000	447,459	12	2	11	Yes
Chr3 (CM046778.1)	40,000,001	45,000,000	417,802	49	2	49	Yes
Chr3 (CM046778.1)	45,000,001	50,000,000	419,030	57	12	57	Yes
Chr3 (CM046778.1)	50,000,001	55,000,000	382,461	89	17	89	Yes
Chr3 (CM046778.1)	55,000,001	58,148,000	233,837	62	18	62	Yes
Chr4 (CM046779.1)	1	5,000,000	409,004	0	0	0	No
Chr4 (CM046779.1)	5,000,001	10,000,000	400,305	32	12	32	Yes
Chr4 (CM046779.1)	10,000,001	15,000,000	362,629	3	0	3	No
Chr4 (CM046779.1)	15,000,001	20,000,000	444,686	25	2	25	Yes
Chr4 (CM046779.1)	20,000,001	25,000,000	366,566	98	14	97	Yes
Chr4 (CM046779.1)	25,000,001	30,000,000	469,282	49	9	49	Yes

Chr4 (CM046779.1)	30,000,001	35,000,000	466,073	109	30	108	Yes
Chr4 (CM046779.1)	35,000,001	40,000,000	450,440	133	10	133	Yes
Chr4 (CM046779.1)	40,000,001	45,000,000	490,971	6	1	6	No
Chr4 (CM046779.1)	45,000,001	50,000,000	426,690	56	3	56	Yes
Chr4 (CM046779.1)	50,000,001	51,822,500	141,230	2	0	2	No
Chr5 (CM046780.1)	1	5,000,000	386,255	0	0	0	No
Chr5 (CM046780.1)	5,000,001	10,000,000	398,825	0	0	0	No
Chr5 (CM046780.1)	10,000,001	15,000,000	414,548	10	3	10	Yes
Chr5 (CM046780.1)	15,000,001	20,000,000	472,264	0	0	0	No
Chr5 (CM046780.1)	20,000,001	25,000,000	451,888	2	0	2	No
Chr5 (CM046780.1)	25,000,001	30,000,000	406,492	5	3	5	No
Chr5 (CM046780.1)	30,000,001	35,000,000	441,267	0	0	0	No
Chr5 (CM046780.1)	35,000,001	40,000,000	462,853	0	0	0	No
Chr5 (CM046780.1)	40,000,001	45,000,000	450,223	6	1	6	No
Chr5 (CM046780.1)	45,000,001	50,000,000	439,685	11	1	11	Yes
Chr5 (CM046780.1)	50,000,001	55,000,000	431,251	10	0	9	No
Chr5 (CM046780.1)	55,000,001	60,000,000	366,496	8	0	8	No
Chr5 (CM046780.1)	60,000,001	61,934,658	117,613	0	0	0	No
Chr6 (CM046781.1)	1	5,000,000	456,560	15	1	15	Yes
Chr6 (CM046781.1)	5,000,001	10,000,000	353,961	160	41	160	Yes
Chr6 (CM046781.1)	10,000,001	15,000,000	341,079	219	53	219	Yes
Chr6 (CM046781.1)	15,000,001	20,000,000	362,239	175	31	175	Yes
Chr6 (CM046781.1)	20,000,001	25,000,000	403,142	1	0	0	No
Chr6 (CM046781.1)	25,000,001	30,000,000	444,884	0	0	0	No
Chr6 (CM046781.1)	30,000,001	35,000,000	453,351	0	0	0	No

Chr6 (CM046781.1)	35,000,001	40,000,000	413,131	0	0	0	No
Chr6 (CM046781.1)	40,000,001	45,000,000	455,108	21	3	21	Yes
Chr6 (CM046781.1)	45,000,001	50,000,000	443,978	25	2	25	Yes
Chr6 (CM046781.1)	50,000,001	55,000,000	405,772	59	6	59	Yes
Chr6 (CM046781.1)	55,000,001	60,000,000	372,841	57	12	57	Yes
Chr6 (CM046781.1)	60,000,001	61,759,399	123,483	3	1	3	No
Chr7 (CM046782.1)	1	5,000,000	391,209	67	12	67	Yes
Chr7 (CM046782.1)	5,000,001	10,000,000	369,508	61	12	60	Yes
Chr7 (CM046782.1)	10,000,001	15,000,000	368,907	114	23	114	Yes
Chr7 (CM046782.1)	15,000,001	20,000,000	367,803	101	17	99	Yes
Chr7 (CM046782.1)	20,000,001	25,000,000	413,749	24	3	24	Yes
Chr7 (CM046782.1)	25,000,001	30,000,000	448,451	11	2	11	Yes
Chr7 (CM046782.1)	30,000,001	35,000,000	440,302	1	0	1	No
Chr7 (CM046782.1)	35,000,001	40,000,000	382,560	0	0	0	No
Chr7 (CM046782.1)	40,000,001	45,000,000	459,599	1	0	1	No
Chr7 (CM046782.1)	45,000,001	50,000,000	451,682	4	0	4	No
Chr7 (CM046782.1)	50,000,001	55,000,000	410,937	22	2	22	Yes
Chr7 (CM046782.1)	55,000,001	60,000,000	402,337	92	15	92	Yes
Chr7 (CM046782.1)	60,000,001	60,847,761	59,905	0	0	0	No
Chr8 (CM046783.1)	1	5,000,000	439,833	7	0	7	No
Chr8 (CM046783.1)	5,000,001	10,000,000	447,381	29	8	29	Yes
Chr8 (CM046783.1)	10,000,001	15,000,000	445,237	15	2	15	Yes
Chr8 (CM046783.1)	15,000,001	20,000,000	450,013	4	0	4	No
Chr8 (CM046783.1)	20,000,001	25,000,000	480,989	13	1	13	Yes
Chr8 (CM046783.1)	25,000,001	30,000,000	423,356	3	0	3	No

Chr8 (CM046783.1)	30,000,001	35,000,000	463,936	6	0	6	No
Chr8 (CM046783.1)	35,000,001	40,000,000	380,869	19	0	18	Yes
Chr8 (CM046783.1)	40,000,001	45,000,000	413,508	1	0	1	No
Chr8 (CM046783.1)	45,000,001	50,000,000	401,750	9	1	9	No
Chr8 (CM046783.1)	50,000,001	55,000,000	398,536	25	2	25	Yes
Chr8 (CM046783.1)	55,000,001	60,000,000	375,182	0	0	0	No
Chr8 (CM046783.1)	60,000,001	60,476,782	41,081	0	0	0	No
Chr9 (CM046784.1)	1	5,000,000	312,711	11	0	11	Yes
Chr9 (CM046784.1)	5,000,001	10,000,000	455,341	29	3	29	Yes
Chr9 (CM046784.1)	10,000,001	15,000,000	468,347	16	0	14	Yes
Chr9 (CM046784.1)	15,000,001	20,000,000	456,589	3	0	3	No
Chr9 (CM046784.1)	20,000,001	25,000,000	438,598	14	0	14	Yes
Chr9 (CM046784.1)	25,000,001	30,000,000	485,690	1	0	1	No
Chr9 (CM046784.1)	30,000,001	35,000,000	454,292	15	3	15	Yes
Chr9 (CM046784.1)	35,000,001	39,826,282	408,573	86	19	86	Yes
Chr10 (CM046785.1)	1	5,000,000	345,799	8	1	8	No
Chr10 (CM046785.1)	5,000,001	10,000,000	359,961	8	2	8	No
Chr10 (CM046785.1)	10,000,001	15,000,000	370,393	100	10	100	Yes
Chr10 (CM046785.1)	15,000,001	20,000,000	413,614	81	12	81	Yes
Chr10 (CM046785.1)	20,000,001	25,000,000	428,819	61	11	61	Yes
Chr10 (CM046785.1)	25,000,001	30,000,000	482,461	38	4	35	Yes
Chr10 (CM046785.1)	30,000,001	35,000,000	390,315	26	3	26	Yes
Chr10 (CM046785.1)	35,000,001	40,000,000	460,331	20	0	20	Yes
Chr10 (CM046785.1)	40,000,001	45,000,000	424,153	33	4	33	Yes
Chr10 (CM046785.1)	45,000,001	50,000,000	412,962	49	8	49	Yes

Chr10 (CM046785.1)	50,000,001	55,000,000	415,696	5	1	5	No
Chr10 (CM046785.1)	55,000,001	56,228,760	89,177	1	0	1	No

Windows were generated from the full chromosome lengths; windows without markers are included. A window was used in the window-based analysis when ≥ 10 moderate-tier markers had A3N read data. Chr5 (CM046780.1) 50,000,001–55,000,000 contains 10 moderate markers, but one had no A3N read data, and the window was therefore excluded.

Table S6. Uniformity of species-diagnostic marker density across 5-Mb windows.

Statistic	Moderate tier	Strict tier
5-Mb windows (n)	124	124
Markers (n)	3,623	594
Mean markers per window	29.2	4.8
Median markers per window	10	1
Range	0–219	0–53
Coefficient of variation	1.42	1.81
Windows without markers (n)	26	58
Windows with ≥ 10 markers (n)	64	25
Gini coefficient	0.67	0.75
Dispersion index vs. length-proportional expectation [a]	55.7	15.1
χ^2 P (uniform by length)	< 0.001	< 0.001
Dispersion index vs. SNP-density-proportional expectation [b]	61.1	16.9
Spearman ρ, markers vs. biallelic SNPs per window	0.12	0.04
P (Spearman)	0.19	0.65

Statistics are computed over all 124 5-Mb windows, including windows without markers. [a] Variance-to-mean ratio of observed counts relative to an expectation proportional to window length (1 = uniform). [b] As [a], but with the expectation proportional to the number of biallelic QUAL ≥ 30 SNPs in each window, which accounts for differences in callable and variable sequence.

Table S7. Distances between adjacent moderate-tier diagnostic markers.

Statistic	Moderate tier
Median inter-marker distance (kb)	1.6
90th percentile inter-marker distance (kb)	257.7
Maximum inter-marker distance (Mb)	20.7
Inter-marker distances > 1 Mb (n)	104
Inter-marker distances > 2 Mb (n)	45

Distances are calculated between consecutive markers on the same chromosome (n = 3,613 intervals).

Table S8. Individual-level nuclear ancestry estimates for A3N oysters.

Individual	Diagnostic-marker-based *C. angulata* dosage (haploid-equivalent copies)	Diagnostic-marker-based *C. gigas* dosage (haploid-equivalent copies)	Diagnostic-marker-based *C. hongkongensis* assignment (haploid-equivalent copies)	HMM copy-weighted *C. gigas* ancestry fraction	HMM *C. gigas* dosage (haploid-equivalent copies)	K-mer-based *C. gigas* dosage (haploid-equivalent copies)
A3N01	2.415	0.478	0.107	0.2653	0.796	0.565
A3N02	2.114	0.789	0.096	0.2883	0.865	0.751
A3N03	2.413	0.460	0.127	0.2305	0.692	0.685
A3N04	2.219	0.673	0.108	0.2936	0.881	0.619
A3N05	2.234	0.683	0.084	0.2885	0.866	0.931

HMM estimates: three-state hidden Markov model (0, 1 or 2 C. gigas-derived copies; Section 2.5.3) applied to moderate-tier C. gigas–C. angulata markers on the C. angulata reference assembly GCA_025612915.1, using A3N site-calls with read depth ≥ 6 (the segments shown in Figure 4). Copy-weighted fraction = (f1 + 2 f2)/3, where fk is the summed span of segments assigned to k C. gigas copies divided by the summed span of all HMM segments of that individual (331.5–388.6 Mb). A segment span is the distance between its first and last supporting marker; intervals between adjacent segments, chromosome ends beyond the outermost markers and chromosomes with < 3 markers are excluded, and single-marker segments contribute 0 bp. HMM dosage = 3 × copy-weighted fraction (same denominator).

Table S9. Genomic coordinates and marker support of inferred ancestry transitions in A3N oysters.

Individual	Reference chromosome accession	Left ancestry state (0G, 1G, or 2G)	Right ancestry state (0G, 1G, or 2G)	Last supporting marker position on the left (bp)	First supporting marker position on the right (bp)	Inter-marker distance (bp)	Left segment span (bp)	Right segment span (bp)	Supporting diagnostic markers in the left segment (n)	Supporting diagnostic markers in the right segment (n)	Inter-marker distance > 2 Mb (Yes/No)	Notes
A3N01	CM046776.1	0G	2G	9,540,007	9,546,622	6615	6,025,637	0	64	1	No	Adjacent to a single-marker segment
A3N01	CM046776.1	2G	0G	9,546,622	9,805,466	258,844	0	1,317,040	1	25	No	Adjacent to a single-marker segment
A3N01	CM046776.1	0G	2G	11,122,506	11,171,205	48,699	1,317,040	285	25	2	No	Adjacent to a segment supported by < 3 markers
A3N01	CM046776.1	2G	0G	11,171,490	11,215,811	44,321	285	294,163	2	16	No	Adjacent to a segment supported by < 3 markers
A3N01	CM046776.1	0G	1G	11,509,974	11,562,997	53,023	294,163	212,514	16	6	No	Both flanking segments supported by >= 5 markers
A3N01	CM046776.1	1G	0G	11,775,511	12,392,014	616,503	212,514	2,942,795	6	48	No	Both flanking segments supported by >= 5 markers
A3N01	CM046776.1	0G	1G	15,334,809	15,434,264	99,455	2,942,795	12,829	48	4	No	Flanking segments supported by 3-4

												markers
A3N01	CM046776.1	1G	0G	15,447,093	15,465,624	18,531	12,829	1,113,249	4	13	No	Flanking segments supported by 3-4 markers
A3N01	CM046776.1	0G	2G	16,578,873	16,839,364	260,491	1,113,249	0	13	1	No	Adjacent to a single-marker segment
A3N01	CM046776.1	2G	0G	16,839,364	16,881,633	42,269	0	9,462,277	1	61	No	Adjacent to a single-marker segment
A3N01	CM046776.1	0G	1G	26,343,910	26,658,157	314,247	9,462,277	40	61	2	No	Adjacent to a segment supported by < 3 markers
A3N01	CM046776.1	1G	0G	26,658,197	26,671,307	13,110	40	9,845,456	2	54	No	Adjacent to a segment supported by < 3 markers
A3N01	CM046777.1	1G	2G	3,156,242	14,623,210	11,466,968	0	178,168	1	2	Yes	Transition interval spans a marker gap > 2 Mb (no diagnostic marker in between); transition position is not resolved within this interval; Adjacent

												to a single-marker segment
A3N01	CM046777.1	2G	1G	14,801,378	49,058,732	34,257,354	178,168	84,415	2	5	Yes	Transition interval spans a marker gap > 2 Mb (no diagnostic marker in between); transition position is not resolved within this interval; Adjacent to a segment supported by < 3 markers
A3N01	CM046777.1	1G	2G	49,143,147	49,143,948	801	84,415	28,142,536	5	21	No	Both flanking segments supported by >= 5 markers
A3N01	CM046778.1	0G	1G	7,601,463	8,418,170	816,707	7,428,395	4,773,671	57	93	No	Both flanking segments supported by >= 5 markers
A3N01	CM046778.1	1G	0G	13,191,841	14,502,060	1,310,219	4,773,671	743,828	93	11	No	Both flanking segments supported by >= 5 markers

A3N01	CM046778.1	0G	1G	15,245,888	16,071,462	825,574	743,828	0	11	1	No	Adjacent to a single-marker segment
A3N01	CM046778.1	1G	0G	16,071,462	16,112,817	41,355	0	14,221,731	1	98	No	Adjacent to a single-marker segment
A3N01	CM046778.1	0G	1G	30,334,548	30,351,356	16,808	14,221,731	1,113,881	98	4	No	Flanking segments supported by 3-4 markers
A3N01	CM046778.1	1G	2G	31,465,237	32,442,787	977,550	1,113,881	1,362,046	4	5	No	Flanking segments supported by 3-4 markers
A3N01	CM046778.1	2G	0G	33,804,833	34,095,194	290,361	1,362,046	616,878	5	39	No	Both flanking segments supported by >= 5 markers
A3N01	CM046778.1	0G	1G	34,712,072	34,985,070	272,998	616,878	9,194,517	39	38	No	Both flanking segments supported by >= 5 markers
A3N01	CM046778.1	1G	2G	44,179,587	44,433,795	254,208	9,194,517	7	38	3	No	Flanking segments supported by 3-4 markers
A3N01	CM046778.1	2G	1G	44,433,802	44,457,664	23,862	7	2,554,678	3	26	No	Flanking segments supported by 3-4 markers
A3N01	CM046778.1	1G	0G	47,012,342	47,380,699	368,357	2,554,678	9,949,159	26	167	No	Both flanking segments

												supported by >= 5 markers
A3N01	CM046779.1	0G	1G	8,646,804	11,056,026	2,409,222	404,469	6,459,975	30	8	Yes	Transition interval spans a marker gap > 2 Mb (no diagnostic marker in between); transition position is not resolved within this interval; Both flanking segments supported by >= 5 markers
A3N01	CM046779.1	1G	0G	17,516,001	17,516,079	78	6,459,975	11,431	8	4	No	Flanking segments supported by 3-4 markers
A3N01	CM046779.1	0G	2G	17,527,510	17,558,519	31,009	11,431	10,420	4	3	No	Flanking segments supported by 3-4 markers
A3N01	CM046779.1	2G	0G	17,568,939	17,792,324	223,385	10,420	18	3	2	No	Adjacent to a segment supported by < 3 markers
A3N01	CM046779.1	0G	1G	17,792,342	19,036,731	1,244,389	18	1,061,393	2	3	No	Adjacent to a segment

												supported by < 3 markers
A3N01	CM046779.1	1G	0G	20,098,124	20,177,799	79,675	1,061,393	496,015	3	11	No	Flanking segments supported by 3-4 markers
A3N01	CM046779.1	0G	2G	20,673,814	20,744,642	70,828	496,015	547,094	11	19	No	Both flanking segments supported by >= 5 markers
A3N01	CM046779.1	2G	0G	21,291,736	21,811,441	519,705	547,094	2,200,180	19	3	No	Flanking segments supported by 3-4 markers
A3N01	CM046779.1	0G	1G	24,011,621	24,012,985	1364	2,200,180	346,621	3	35	No	Flanking segments supported by 3-4 markers
A3N01	CM046779.1	1G	2G	24,359,606	25,661,464	1,301,858	346,621	1,897,323	35	14	No	Both flanking segments supported by >= 5 markers
A3N01	CM046779.1	2G	1G	27,558,787	28,259,428	700,641	1,897,323	657,474	14	11	No	Both flanking segments supported by >= 5 markers
A3N01	CM046779.1	1G	2G	28,916,902	29,192,185	275,283	657,474	21,024,949	11	182	No	Both flanking segments supported by >= 5 markers

A3N01	CM046780.1	2G	1G	11,161,421	24,776,200	13,614,779	295,294	2,936,856	9	3	Yes	Transition interval spans a marker gap > 2 Mb (no diagnostic marker in between); transition position is not resolved within this interval; Flanking segments supported by 3-4 markers
A3N01	CM046780.1	1G	0G	27,713,056	27,967,125	254,069	2,936,856	1,789,484	3	4	No	Flanking segments supported by 3-4 markers
A3N01	CM046780.1	0G	1G	29,756,609	43,195,180	13,438,571	1,789,484	7,725,692	4	11	Yes	Transition interval spans a marker gap > 2 Mb (no diagnostic marker in between); transition position is not resolved within this interval; Flanking segments supported

												by 3-4 markers
A3N01	CM046780.1	1G	0G	50,920,872	51,598,189	677,317	7,725,692	4,146,282	11	7	No	Both flanking segments supported by >= 5 markers
A3N01	CM046781.1	0G	2G	7,048,375	7,097,912	49,537	5,793,260	81,108	12	3	No	Flanking segments supported by 3-4 markers
A3N01	CM046781.1	2G	0G	7,179,020	7,206,989	27,969	81,108	4,720,465	3	196	No	Flanking segments supported by 3-4 markers
A3N01	CM046781.1	0G	1G	11,927,454	11,930,479	3025	4,720,465	7340	196	7	No	Both flanking segments supported by >= 5 markers
A3N01	CM046781.1	1G	0G	11,937,819	12,011,779	73,960	7340	30,867,601	7	282	No	Both flanking segments supported by >= 5 markers
A3N01	CM046781.1	0G	2G	42,879,380	44,038,805	1,159,425	30,867,601	7122	282	4	No	Flanking segments supported by 3-4 markers
A3N01	CM046781.1	2G	0G	44,045,927	44,348,511	302,584	7122	118,316	4	2	No	Adjacent to a segment supported by < 3 markers

A3N01	CM046781.1	0G	2G	44,466,827	44,510,413	43,586	118,316	0	2	1	No	Adjacent to a single-marker segment
A3N01	CM046781.1	2G	0G	44,510,413	44,700,060	189,647	0	1,263,735	1	22	No	Adjacent to a single-marker segment
A3N01	CM046781.1	0G	1G	45,963,795	46,055,187	91,392	1,263,735	8,374,188	22	40	No	Both flanking segments supported by >= 5 markers
A3N01	CM046781.1	1G	0G	54,429,375	54,589,616	160,241	8,374,188	1,096,990	40	18	No	Both flanking segments supported by >= 5 markers
A3N01	CM046781.1	0G	1G	55,686,606	55,789,766	103,160	1,096,990	51	18	2	No	Adjacent to a segment supported by < 3 markers
A3N01	CM046781.1	1G	0G	55,789,817	55,819,750	29,933	51	5,367,724	2	47	No	Adjacent to a segment supported by < 3 markers
A3N01	CM046782.1	2G	1G	1,149,249	1,177,276	28,027	1854	543,460	4	8	No	Flanking segments supported by 3-4 markers
A3N01	CM046782.1	1G	0G	1,720,736	2,064,639	343,903	543,460	503,923	8	3	No	Flanking segments supported by 3-4 markers

A3N01	CM046782.1	0G	2G	2,568,562	2,850,240	281,678	503,923	7140	3	7	No	Flanking segments supported by 3-4 markers
A3N01	CM046782.1	2G	1G	2,857,380	3,163,050	305,670	7140	9,503,920	7	104	No	Both flanking segments supported by >= 5 markers
A3N01	CM046782.1	1G	2G	12,666,970	14,063,913	1,396,943	9,503,920	13	104	4	No	Flanking segments supported by 3-4 markers
A3N01	CM046782.1	2G	1G	14,063,926	14,734,201	670,275	13	307,644	4	15	No	Flanking segments supported by 3-4 markers
A3N01	CM046782.1	1G	2G	15,041,845	15,090,020	48,175	307,644	84,570	15	6	No	Both flanking segments supported by >= 5 markers
A3N01	CM046782.1	2G	1G	15,174,590	15,231,624	57,034	84,570	1,900,528	6	7	No	Both flanking segments supported by >= 5 markers
A3N01	CM046782.1	1G	2G	17,132,152	17,135,916	3764	1,900,528	55	7	3	No	Flanking segments supported by 3-4 markers
A3N01	CM046782.1	2G	1G	17,135,971	17,136,687	716	55	31,783,935	3	59	No	Flanking segments supported by 3-4 markers

A3N01	CM046782.1	1G	2G	48,920,622	51,087,587	2,166,965	31,783,935	555	59	2	Yes	Transition interval spans a marker gap > 2 Mb (no diagnostic marker in between); transition position is not resolved within this interval; Adjacent to a segment supported by < 3 markers
A3N01	CM046782.1	2G	1G	51,088,142	51,221,278	133,136	555	1,092,953	2	4	No	Adjacent to a segment supported by < 3 markers
A3N01	CM046782.1	1G	0G	52,314,231	53,479,092	1,164,861	1,092,953	2,117,906	4	23	No	Flanking segments supported by 3-4 markers
A3N01	CM046782.1	0G	2G	55,596,998	55,645,071	48,073	2,117,906	18	23	2	No	Adjacent to a segment supported by < 3 markers
A3N01	CM046782.1	2G	1G	55,645,089	55,801,848	156,759	18	960,100	2	15	No	Adjacent to a segment supported by < 3

												markers
A3N01	CM046782.1	1G	0G	56,761,948	56,872,230	110,282	960,100	57,985	15	10	No	Both flanking segments supported by >= 5 markers
A3N01	CM046782.1	0G	1G	56,930,215	56,965,435	35,220	57,985	26,640	10	3	No	Flanking segments supported by 3-4 markers
A3N01	CM046782.1	1G	0G	56,992,075	57,006,132	14,057	26,640	530,129	3	20	No	Flanking segments supported by 3-4 markers
A3N01	CM046782.1	0G	2G	57,536,261	57,551,375	15,114	530,129	241,632	20	2	No	Adjacent to a segment supported by < 3 markers
A3N01	CM046782.1	2G	0G	57,793,007	58,016,146	223,139	241,632	1,956,331	2	12	No	Adjacent to a segment supported by < 3 markers
A3N01	CM046783.1	0G	1G	2,504,850	3,670,005	1,165,155	2,475,201	1,872,123	6	2	No	Adjacent to a segment supported by < 3 markers
A3N01	CM046783.1	1G	2G	5,542,128	5,927,845	385,717	1,872,123	31	2	2	No	Adjacent to a segment supported by < 3 markers
A3N01	CM046783.1	2G	1G	5,927,876	5,954,360	26,484	31	3,971,644	2	10	No	Adjacent

												to a segment supported by < 3 markers
A3N01	CM046783.1	1G	2G	9,926,004	11,082,688	1,156,684	3,971,644	0	10	1	No	Adjacent to a single-marker segment
A3N01	CM046783.1	2G	1G	11,082,688	11,927,004	844,316	0	7,539,130	1	11	No	Adjacent to a single-marker segment
A3N01	CM046783.1	1G	2G	19,466,134	20,054,493	588,359	7,539,130	1	11	2	No	Adjacent to a segment supported by < 3 markers
A3N01	CM046783.1	2G	0G	20,054,494	20,070,970	16,476	1	10,946,146	2	3	No	Adjacent to a segment supported by < 3 markers
A3N01	CM046783.1	0G	2G	31,017,116	34,982,859	3,965,743	10,946,146	348,515	3	3	Yes	Transition interval spans a marker gap > 2 Mb (no diagnostic marker in between); transition position is not resolved within this interval; Flanking segments supported

												by 3-4 markers
A3N01	CM046783.1	2G	1G	35,331,374	35,332,114	740	348,515	43	3	3	No	Flanking segments supported by 3-4 markers
A3N01	CM046783.1	1G	2G	35,332,157	36,847,076	1,514,919	43	14,822,618	3	24	No	Flanking segments supported by 3-4 markers
A3N01	CM046783.1	2G	0G	51,669,694	52,426,188	756,494	14,822,618	4987	24	9	No	Both flanking segments supported by >= 5 markers
A3N01	CM046784.1	0G	1G	3,868,587	4,122,539	253,952	157,065	3,165,757	4	13	No	Flanking segments supported by 3-4 markers
A3N01	CM046784.1	1G	2G	7,288,296	8,433,188	1,144,892	3,165,757	1,427,227	13	11	No	Both flanking segments supported by >= 5 markers
A3N01	CM046784.1	2G	1G	9,860,415	10,082,037	221,622	1,427,227	4,558,846	11	8	No	Both flanking segments supported by >= 5 markers
A3N01	CM046784.1	1G	2G	14,640,883	15,788,290	1,147,407	4,558,846	3	8	2	No	Adjacent to a segment supported by < 3 markers

A3N01	CM046784.1	2G	1G	15,788,293	16,190,229	401,936	3	16,562,750	2	17	No	Adjacent to a segment supported by < 3 markers
A3N01	CM046784.1	1G	2G	32,752,979	34,989,469	2,236,490	16,562,750	69	17	3	Yes	Transition interval spans a marker gap > 2 Mb (no diagnostic marker in between); transition position is not resolved within this interval; Flanking segments supported by 3-4 markers
A3N01	CM046784.1	2G	1G	34,989,538	35,469,278	479,740	69	100,486	3	8	No	Flanking segments supported by 3-4 markers
A3N01	CM046784.1	1G	0G	35,569,764	35,911,631	341,867	100,486	122,784	8	20	No	Both flanking segments supported by >= 5 markers
A3N01	CM046784.1	0G	1G	36,034,415	36,125,208	90,793	122,784	16,499	20	8	No	Both flanking segments supported by >= 5 markers

A3N01	CM046784.1	1G	2G	36,141,707	36,155,535	13,828	16,499	301,170	8	16	No	Both flanking segments supported by >= 5 markers
A3N01	CM046785.1	1G	0G	14,567,082	15,458,537	891,455	12,344,077	177,136	72	12	No	Both flanking segments supported by >= 5 markers
A3N01	CM046785.1	0G	1G	15,635,673	15,651,738	16,065	177,136	1603	12	5	No	Both flanking segments supported by >= 5 markers
A3N01	CM046785.1	1G	0G	15,653,341	15,979,845	326,504	1603	5,860,884	5	95	No	Both flanking segments supported by >= 5 markers
A3N01	CM046785.1	0G	1G	21,840,729	22,212,038	371,309	5,860,884	6,315,808	95	21	No	Both flanking segments supported by >= 5 markers
A3N01	CM046785.1	1G	2G	28,527,846	28,624,168	96,322	6,315,808	1,434,904	21	7	No	Both flanking segments supported by >= 5 markers
A3N01	CM046785.1	2G	0G	30,059,072	30,094,675	35,603	1,434,904	5,227,684	7	23	No	Both flanking segments supported by >= 5 markers

A3N01	CM046785.1	0G	1G	35,322,359	38,681,683	3,359,324	5,227,684	1,082,104	23	11	Yes	Transition interval spans a marker gap > 2 Mb (no diagnostic marker in between); transition position is not resolved within this interval; Both flanking segments supported by >= 5 markers
A3N01	CM046785.1	1G	2G	39,763,787	40,143,450	379,663	1,082,104	877,933	11	2	No	Adjacent to a segment supported by < 3 markers
A3N01	CM046785.1	2G	1G	41,021,383	41,373,081	351,698	877,933	2,214,024	2	11	No	Adjacent to a segment supported by < 3 markers
A3N01	CM046785.1	1G	2G	43,587,105	44,035,115	448,010	2,214,024	7	11	3	No	Flanking segments supported by 3-4 markers
A3N01	CM046785.1	2G	1G	44,035,122	44,085,032	49,910	7	11,748,647	3	35	No	Flanking segments supported by 3-4 markers

A3N02	CM046776.1	2G	0G	4,453,952	5,813,347	1,359,395	939,582	0	9	1	No	Adjacent to a single-marker segment
A3N02	CM046776.1	0G	2G	5,813,347	5,861,938	48,591	0	5,353,891	1	66	No	Adjacent to a single-marker segment
A3N02	CM046776.1	2G	0G	11,215,829	11,277,605	61,776	5,353,891	18	66	3	No	Flanking segments supported by 3-4 markers
A3N02	CM046776.1	0G	2G	11,277,623	11,312,347	34,724	18	4,170,041	3	46	No	Flanking segments supported by 3-4 markers
A3N02	CM046776.1	2G	0G	15,482,388	15,482,512	124	4,170,041	0	46	1	No	Adjacent to a single-marker segment
A3N02	CM046776.1	0G	2G	15,482,512	15,484,658	2146	0	1,094,215	1	10	No	Adjacent to a single-marker segment
A3N02	CM046776.1	2G	1G	16,578,873	16,839,364	260,491	1,094,215	861,788	10	15	No	Both flanking segments supported by >= 5 markers
A3N02	CM046776.1	1G	0G	17,701,152	21,506,391	3,805,239	861,788	869,357	15	27	Yes	Transition interval spans a marker gap > 2 Mb (no diagnostic marker in between); transition position is

												not resolved within this interval; Both flanking segments supported by >= 5 markers
A3N02	CM046776.1	0G	2G	22,375,748	22,485,248	109,500	869,357	0	27	1	No	Adjacent to a single-marker segment
A3N02	CM046776.1	2G	0G	22,485,248	22,537,263	52,015	0	3,806,647	1	20	No	Adjacent to a single-marker segment
A3N02	CM046776.1	0G	2G	26,343,910	26,658,157	314,247	3,806,647	0	20	1	No	Adjacent to a single-marker segment
A3N02	CM046776.1	2G	0G	26,658,157	26,671,307	13,150	0	688,521	1	15	No	Adjacent to a single-marker segment
A3N02	CM046776.1	0G	1G	27,359,828	27,369,505	9677	688,521	50	15	2	No	Adjacent to a segment supported by < 3 markers
A3N02	CM046776.1	1G	0G	27,369,555	27,432,786	63,231	50	3,205,085	2	37	No	Adjacent to a segment supported by < 3 markers
A3N02	CM046776.1	0G	2G	30,637,871	36,516,763	5,878,892	3,205,085	0	37	1	Yes	Transition interval spans a marker gap > 2

												Mb (no diagnostic marker in between); transition position is not resolved within this interval; Adjacent to a single-marker segment
A3N02	CM046777.1	1G	2G	49,807,828	50,289,549	481,721	35,184,522	0	31	1	No	Adjacent to a single-marker segment
A3N02	CM046777.1	2G	1G	50,289,549	52,009,449	1,719,900	0	10,060,698	1	4	No	Adjacent to a single-marker segment
A3N02	CM046777.1	1G	2G	62,070,147	77,286,484	15,216,337	10,060,698	0	4	1	Yes	Transition interval spans a marker gap > 2 Mb (no diagnostic marker in between); transition position is not resolved within this interval; Adjacent to a single-marker segment
A3N02	CM046778.1	1G	0G	3,194,474	3,677,047	482,573	3,021,406	94,809	18	6	No	Both flanking

												segments supported by >= 5 markers
A3N02	CM046778.1	0G	1G	3,771,856	4,254,716	482,860	94,809	5,949,558	6	40	No	Both flanking segments supported by >= 5 markers
A3N02	CM046778.1	1G	2G	10,204,274	10,322,388	118,114	5,949,558	10,970	40	5	No	Both flanking segments supported by >= 5 markers
A3N02	CM046778.1	2G	0G	10,333,358	10,346,302	12,944	10,970	167,848	5	3	No	Flanking segments supported by 3-4 markers
A3N02	CM046778.1	0G	2G	10,514,150	10,548,313	34,163	167,848	0	3	1	No	Adjacent to a single-marker segment
A3N02	CM046778.1	2G	1G	10,548,313	10,563,467	15,154	0	10,723,607	1	153	No	Adjacent to a single-marker segment
A3N02	CM046778.1	1G	0G	21,287,074	23,392,007	2,104,933	10,723,607	6,942,886	153	14	Yes	Transition interval spans a marker gap > 2 Mb (no diagnostic marker in between); transition position is not resolved within this

												interval; Both flanking segments supported by >= 5 markers
A3N02	CM046778.1	0G	2G	30,334,893	30,351,356	16,463	6,942,886	0	14	1	No	Adjacent to a single-marker segment
A3N02	CM046778.1	2G	0G	30,351,356	31,064,162	712,806	0	401,075	1	2	No	Adjacent to a single-marker segment
A3N02	CM046778.1	0G	1G	31,465,237	31,748,215	282,978	401,075	2,290,942	2	10	No	Adjacent to a segment supported by < 3 markers
A3N02	CM046778.1	1G	0G	34,039,157	34,095,194	56,037	2,290,942	889,876	10	42	No	Both flanking segments supported by >= 5 markers
A3N02	CM046778.1	0G	1G	34,985,070	35,086,867	101,797	889,876	1,220,309	42	4	No	Flanking segments supported by 3-4 markers
A3N02	CM046778.1	1G	0G	36,307,176	36,536,647	229,471	1,220,309	1,113,710	4	4	No	Flanking segments supported by 3-4 markers
A3N02	CM046778.1	0G	1G	37,650,357	38,407,694	757,337	1,113,710	5,734,063	4	23	No	Flanking segments supported by 3-4 markers

A3N02	CM046778.1	1G	0G	44,141,757	44,142,080	323	5,734,063	5731	23	5	No	Both flanking segments supported by >= 5 markers
A3N02	CM046778.1	0G	1G	44,147,811	44,175,162	27,351	5731	6,504,572	5	52	No	Both flanking segments supported by >= 5 markers
A3N02	CM046778.1	1G	0G	50,679,734	51,511,226	831,492	6,504,572	701,940	52	7	No	Both flanking segments supported by >= 5 markers
A3N02	CM046778.1	0G	1G	52,213,166	52,680,299	467,133	701,940	2,706,366	7	82	No	Both flanking segments supported by >= 5 markers
A3N02	CM046778.1	1G	2G	55,386,665	55,431,559	44,894	2,706,366	153,464	82	3	No	Flanking segments supported by 3-4 markers
A3N02	CM046778.1	2G	1G	55,585,023	55,647,138	62,115	153,464	10,021	3	3	No	Flanking segments supported by 3-4 markers
A3N02	CM046778.1	1G	2G	55,657,159	55,763,472	106,313	10,021	1,566,273	3	5	No	Flanking segments supported by 3-4 markers
A3N02	CM046779.1	1G	0G	11,056,473	17,515,790	6,459,317	2,860,754	276,552	32	20	Yes	Transition interval spans a marker

												gap > 2 Mb (no diagnostic marker in between); transition position is not resolved within this interval; Both flanking segments supported by >= 5 markers
A3N02	CM046779.1	0G	1G	17,792,342	17,935,422	143,080	276,552	402,873	20	2	No	Adjacent to a segment supported by < 3 markers
A3N02	CM046779.1	1G	0G	18,338,295	19,036,731	698,436	402,873	1,637,083	2	26	No	Adjacent to a segment supported by < 3 markers
A3N02	CM046779.1	0G	1G	20,673,814	20,744,621	70,807	1,637,083	547,115	26	12	No	Both flanking segments supported by >= 5 markers
A3N02	CM046779.1	1G	0G	21,291,736	21,788,168	496,432	547,115	2,411,689	12	17	No	Both flanking segments supported by >= 5 markers
A3N02	CM046779.1	0G	1G	24,199,857	24,233,564	33,707	2,411,689	21	17	4	No	Flanking segments

												supported by 3-4 markers
A3N02	CM046779.1	1G	0G	24,233,585	24,235,202	1617	21	124,404	4	27	No	Flanking segments supported by 3-4 markers
A3N02	CM046779.1	0G	1G	24,359,606	24,945,762	586,156	124,404	2,613,025	27	15	No	Both flanking segments supported by >= 5 markers
A3N02	CM046779.1	1G	0G	27,558,787	28,259,428	700,641	2,613,025	378,840	15	10	No	Both flanking segments supported by >= 5 markers
A3N02	CM046779.1	0G	2G	28,638,268	28,660,142	21,874	378,840	4685	10	2	No	Adjacent to a segment supported by < 3 markers
A3N02	CM046779.1	2G	1G	28,664,827	28,859,774	194,947	4685	2,358,479	2	37	No	Adjacent to a segment supported by < 3 markers
A3N02	CM046779.1	1G	2G	31,218,253	32,566,855	1,348,602	2,358,479	125,753	37	4	No	Flanking segments supported by 3-4 markers
A3N02	CM046779.1	2G	1G	32,692,608	32,769,658	77,050	125,753	1,809,921	4	36	No	Flanking segments supported by 3-4 markers

A3N02	CM046779.1	1G	2G	34,579,579	34,595,703	16,124	1,809,921	0	36	1	No	Adjacent to a single-marker segment
A3N02	CM046779.1	2G	1G	34,595,703	34,600,045	4342	0	1,184,645	1	18	No	Adjacent to a single-marker segment
A3N02	CM046779.1	1G	2G	35,784,690	35,797,815	13,125	1,184,645	7062	18	5	No	Both flanking segments supported by >= 5 markers
A3N02	CM046779.1	2G	1G	35,804,877	35,993,692	188,815	7062	1,849,076	5	31	No	Both flanking segments supported by >= 5 markers
A3N02	CM046779.1	1G	2G	37,842,768	38,064,235	221,467	1,849,076	175,384	31	3	No	Flanking segments supported by 3-4 markers
A3N02	CM046779.1	2G	1G	38,239,619	38,487,609	247,990	175,384	6,906,700	3	40	No	Flanking segments supported by 3-4 markers
A3N02	CM046779.1	1G	0G	45,394,309	45,403,278	8969	6,906,700	533,671	40	9	No	Both flanking segments supported by >= 5 markers
A3N02	CM046779.1	0G	1G	45,936,949	45,959,515	22,566	533,671	4,373,084	9	31	No	Both flanking segments supported by >= 5 markers

A3N02	CM046780.1	1G	0G	10,866,127	10,886,630	20,503	0	274,791	1	8	No	Adjacent to a single-marker segment
A3N02	CM046780.1	0G	1G	11,161,421	24,776,200	13,614,779	274,791	26,917,489	8	14	Yes	Transition interval spans a marker gap > 2 Mb (no diagnostic marker in between); transition position is not resolved within this interval; Both flanking segments supported by >= 5 markers
A3N02	CM046780.1	1G	2G	51,693,689	54,921,457	3,227,768	26,917,489	5451	14	3	Yes	Transition interval spans a marker gap > 2 Mb (no diagnostic marker in between); transition position is not resolved within this interval; Flanking segments supported

												by 3-4 markers
A3N02	CM046780.1	2G	1G	54,926,908	55,737,582	810,674	5451	6889	3	3	No	Flanking segments supported by 3-4 markers
A3N02	CM046781.1	1G	0G	3,004,376	4,609,424	1,605,048	2,314,134	7,318,030	6	218	No	Both flanking segments supported by >= 5 markers
A3N02	CM046781.1	0G	1G	11,927,454	11,928,631	1177	7,318,030	9188	218	14	No	Both flanking segments supported by >= 5 markers
A3N02	CM046781.1	1G	0G	11,937,819	11,966,078	28,259	9188	30,913,302	14	281	No	Both flanking segments supported by >= 5 markers
A3N02	CM046781.1	0G	2G	42,879,380	44,038,922	1,159,542	30,913,302	0	281	1	No	Adjacent to a single-marker segment
A3N02	CM046781.1	2G	0G	44,038,922	44,045,927	7005	0	3,199,014	1	21	No	Adjacent to a single-marker segment
A3N02	CM046781.1	0G	1G	47,244,941	49,921,723	2,676,782	3,199,014	2,599,588	21	4	Yes	Transition interval spans a marker gap > 2 Mb (no diagnostic marker in

												between); transition position is not resolved within this interval; Flanking segments supported by 3-4 markers
A3N02	CM046781.1	1G	2G	52,521,311	52,569,300	47,989	2,599,588	1,046,855	4	24	No	Flanking segments supported by 3-4 markers
A3N02	CM046781.1	2G	1G	53,616,155	53,741,065	124,910	1,046,855	401,878	24	2	No	Adjacent to a segment supported by < 3 markers
A3N02	CM046781.1	1G	0G	54,142,943	54,143,252	309	401,878	2,706,161	2	51	No	Adjacent to a segment supported by < 3 markers
A3N02	CM046781.1	0G	1G	56,849,413	58,140,447	1,291,034	2,706,161	0	51	1	No	Adjacent to a single-marker segment
A3N02	CM046781.1	1G	0G	58,140,447	58,716,438	575,991	0	2,471,036	1	16	No	Adjacent to a single-marker segment
A3N02	CM046782.1	2G	0G	7,124,452	7,496,503	372,051	5,975,212	1731	60	3	No	Flanking segments supported by 3-4 markers

A3N02	CM046782.1	0G	2G	7,498,234	7,911,629	413,395	1731	2,345,718	3	21	No	Flanking segments supported by 3-4 markers
A3N02	CM046782.1	2G	0G	10,257,347	10,265,189	7842	2,345,718	36	21	2	No	Adjacent to a segment supported by < 3 markers
A3N02	CM046782.1	0G	2G	10,265,225	10,271,952	6727	36	0	2	1	No	Adjacent to a single-marker segment
A3N02	CM046782.1	2G	0G	10,271,952	10,275,587	3635	0	0	1	1	No	Adjacent to a single-marker segment
A3N02	CM046782.1	0G	2G	10,275,587	10,301,810	26,223	0	2,310,160	1	40	No	Adjacent to a single-marker segment
A3N02	CM046782.1	2G	0G	12,611,970	12,619,157	7187	2,310,160	0	40	1	No	Adjacent to a single-marker segment
A3N02	CM046782.1	0G	2G	12,619,157	12,621,589	2432	0	5,585,337	1	73	No	Adjacent to a single-marker segment
A3N02	CM046782.1	2G	0G	18,206,926	18,257,985	51,059	5,585,337	119,596	73	2	No	Adjacent to a segment supported by < 3 markers
A3N02	CM046782.1	0G	2G	18,377,581	18,486,070	108,489	119,596	3,395,347	2	16	No	Adjacent to a segment supported by < 3 markers

A3N02	CM046782.1	2G	1G	21,881,417	27,516,315	5,634,898	3,395,347	18,521,504	16	7	Yes	Transition interval spans a marker gap > 2 Mb (no diagnostic marker in between); transition position is not resolved within this interval; Both flanking segments supported by >= 5 markers
A3N02	CM046782.1	1G	0G	46,037,819	48,920,622	2,882,803	18,521,504	4,576,614	7	13	Yes	Transition interval spans a marker gap > 2 Mb (no diagnostic marker in between); transition position is not resolved within this interval; Both flanking segments supported by >= 5 markers

A3N02	CM046782.1	0G	2G	53,497,236	54,100,172	602,936	4,576,614	0	13	1	No	Adjacent to a single-marker segment
A3N02	CM046782.1	2G	0G	54,100,172	54,682,961	582,789	0	914,037	1	11	No	Adjacent to a single-marker segment
A3N02	CM046782.1	0G	1G	55,596,998	55,761,858	164,860	914,037	40,025	11	5	No	Both flanking segments supported by >= 5 markers
A3N02	CM046782.1	1G	0G	55,801,883	55,833,704	31,821	40,025	31,178	5	5	No	Both flanking segments supported by >= 5 markers
A3N02	CM046782.1	0G	2G	55,864,882	55,926,244	61,362	31,178	0	5	1	No	Adjacent to a single-marker segment
A3N02	CM046782.1	2G	0G	55,926,244	55,935,312	9068	0	994,903	1	18	No	Adjacent to a single-marker segment
A3N02	CM046782.1	0G	2G	56,930,215	56,965,435	35,220	994,903	0	18	1	No	Adjacent to a single-marker segment
A3N02	CM046782.1	2G	1G	56,965,435	56,984,770	19,335	0	7305	1	8	No	Adjacent to a single-marker segment
A3N02	CM046782.1	1G	0G	56,992,075	57,006,050	13,975	7305	123,841	8	6	No	Both flanking segments supported by >= 5 markers

A3N02	CM046782.1	0G	2G	57,129,891	57,255,126	125,235	123,841	39,469	6	4	No	Flanking segments supported by 3-4 markers
A3N02	CM046782.1	2G	0G	57,294,595	57,486,764	192,169	39,469	6207	4	2	No	Adjacent to a segment supported by < 3 markers
A3N02	CM046782.1	0G	2G	57,492,971	57,505,910	12,939	6207	0	2	1	No	Adjacent to a single-marker segment
A3N02	CM046782.1	2G	0G	57,505,910	57,505,914	4	0	2,466,563	1	19	No	Adjacent to a single-marker segment
A3N02	CM046783.1	0G	1G	2,184,069	2,504,850	320,781	2,154,420	16,961,284	4	31	No	Flanking segments supported by 3-4 markers
A3N02	CM046783.1	1G	2G	19,466,134	20,054,493	588,359	16,961,284	16,477	31	3	No	Flanking segments supported by 3-4 markers
A3N02	CM046783.1	2G	0G	20,070,970	21,503,407	1,432,437	16,477	3160	3	8	No	Flanking segments supported by 3-4 markers
A3N02	CM046783.1	0G	1G	21,506,567	25,993,580	4,487,013	3160	19,746,789	8	24	Yes	Transition interval spans a marker gap > 2 Mb (no diagnostic marker in between);

												transition position is not resolved within this interval; Both flanking segments supported by >= 5 markers
A3N02	CM046783.1	1G	2G	45,740,369	49,881,292	4,140,923	19,746,789	1,017,080	24	10	Yes	Transition interval spans a marker gap > 2 Mb (no diagnostic marker in between); transition position is not resolved within this interval; Both flanking segments supported by >= 5 markers
A3N02	CM046783.1	2G	1G	50,898,372	51,669,523	771,151	1,017,080	760,021	10	4	No	Flanking segments supported by 3-4 markers
A3N02	CM046783.1	1G	0G	52,429,544	52,431,076	1532	760,021	99	4	5	No	Flanking segments supported by 3-4 markers

A3N02	CM046784.1	1G	0G	7,262,471	8,433,188	1,170,717	2,880,306	1,122,898	15	9	No	Both flanking segments supported by >= 5 markers
A3N02	CM046784.1	0G	1G	9,556,086	9,860,415	304,329	1,122,898	391,923	9	5	No	Both flanking segments supported by >= 5 markers
A3N02	CM046784.1	1G	0G	10,252,338	13,028,669	2,776,331	391,923	1,611,166	5	9	Yes	Transition interval spans a marker gap > 2 Mb (no diagnostic marker in between); transition position is not resolved within this interval; Both flanking segments supported by >= 5 markers
A3N02	CM046784.1	0G	2G	14,639,835	15,788,290	1,148,455	1,611,166	3	9	2	No	Adjacent to a segment supported by < 3 markers
A3N02	CM046784.1	2G	1G	15,788,293	20,012,754	4,224,461	3	12,752,863	2	16	Yes	Transition interval spans a marker

												gap > 2 Mb (no diagnostic marker in between); transition position is not resolved within this interval; Adjacent to a segment supported by < 3 markers
A3N02	CM046784.1	1G	2G	32,765,617	34,989,469	2,223,852	12,752,863	69	16	3	Yes	Transition interval spans a marker gap > 2 Mb (no diagnostic marker in between); transition position is not resolved within this interval; Flanking segments supported by 3-4 markers
A3N02	CM046784.1	2G	1G	34,989,538	35,002,383	12,845	69	899,145	3	7	No	Flanking segments supported by 3-4 markers

A3N02	CM046784.1	1G	0G	35,901,528	35,911,631	10,103	899,145	41,486	7	18	No	Both flanking segments supported by >= 5 markers
A3N02	CM046784.1	0G	1G	35,953,117	35,954,401	1284	41,486	170,828	18	8	No	Both flanking segments supported by >= 5 markers
A3N02	CM046784.1	1G	0G	36,125,229	36,125,808	579	170,828	2338	8	7	No	Both flanking segments supported by >= 5 markers
A3N02	CM046784.1	0G	2G	36,128,146	36,132,491	4345	2338	324,214	7	23	No	Both flanking segments supported by >= 5 markers
A3N02	CM046785.1	0G	1G	4,785,560	5,770,947	985,387	2,562,555	15,702,426	8	157	No	Both flanking segments supported by >= 5 markers
A3N02	CM046785.1	1G	2G	21,473,373	21,489,441	16,068	15,702,426	38	157	2	No	Adjacent to a segment supported by < 3 markers
A3N02	CM046785.1	2G	1G	21,489,479	21,701,327	211,848	38	5,242,919	2	21	No	Adjacent to a segment supported by < 3 markers

A3N02	CM046785.1	1G	0G	26,944,246	28,344,006	1,399,760	5,242,919	802	21	2	No	Adjacent to a segment supported by < 3 markers
A3N02	CM046785.1	0G	2G	28,344,808	28,345,743	935	802	0	2	1	No	Adjacent to a single-marker segment
A3N02	CM046785.1	2G	0G	28,345,743	28,357,717	11,974	0	0	1	1	No	Adjacent to a single-marker segment
A3N02	CM046785.1	0G	2G	28,357,717	28,527,846	170,129	0	96,428	1	4	No	Adjacent to a single-marker segment
A3N02	CM046785.1	2G	1G	28,624,274	29,018,372	394,098	96,428	7247	4	3	No	Flanking segments supported by 3-4 markers
A3N02	CM046785.1	1G	2G	29,025,619	29,795,550	769,931	7247	214,807	3	9	No	Flanking segments supported by 3-4 markers
A3N02	CM046785.1	2G	1G	30,010,357	30,094,675	84,318	214,807	14,347,732	9	46	No	Both flanking segments supported by >= 5 markers
A3N02	CM046785.1	1G	2G	44,442,407	46,019,152	1,576,745	14,347,732	60,271	46	2	No	Adjacent to a segment supported by < 3 markers
A3N02	CM046785.1	2G	1G	46,079,423	46,079,428	5	60,271	9,754,251	2	36	No	Adjacent to a segment

												supported by < 3 markers
A3N03	CM046776.1	2G	0G	3,669,989	5,813,347	2,143,358	2186	0	6	1	Yes	Transition interval spans a marker gap > 2 Mb (no diagnostic marker in between); transition position is not resolved within this interval; Adjacent to a single-marker segment
A3N03	CM046776.1	0G	2G	5,813,347	5,861,938	48,591	0	3,449,028	1	22	No	Adjacent to a single-marker segment
A3N03	CM046776.1	2G	0G	9,310,966	9,311,174	208	3,449,028	47	22	6	No	Both flanking segments supported by >= 5 markers
A3N03	CM046776.1	0G	2G	9,311,221	9,311,718	497	47	6,335,893	6	70	No	Both flanking segments supported by >= 5 markers
A3N03	CM046776.1	2G	1G	15,647,611	16,839,364	1,191,753	6,335,893	42,269	70	3	No	Flanking segments supported by 3-4 markers

A3N03	CM046776.1	1G	2G	16,881,633	17,473,949	592,316	42,269	227,203	3	15	No	Flanking segments supported by 3-4 markers
A3N03	CM046776.1	2G	1G	17,701,152	21,473,001	3,771,849	227,203	1,208,823	15	24	Yes	Transition interval spans a marker gap > 2 Mb (no diagnostic marker in between); transition position is not resolved within this interval; Both flanking segments supported by >= 5 markers
A3N03	CM046776.1	1G	2G	22,681,824	23,120,292	438,468	1,208,823	30,570	24	3	No	Flanking segments supported by 3-4 markers
A3N03	CM046776.1	2G	1G	23,150,862	23,535,569	384,707	30,570	7,102,302	3	39	No	Flanking segments supported by 3-4 markers
A3N03	CM046777.1	2G	1G	14,801,378	38,511,280	23,709,902	178,168	0	2	1	Yes	Transition interval spans a marker gap > 2 Mb (no diagnostic

												marker in between); transition position is not resolved within this interval; Adjacent to a single-marker segment
A3N03	CM046777.1	1G	2G	38,511,280	49,143,938	10,632,658	0	1,145,611	1	15	Yes	Transition interval spans a marker gap > 2 Mb (no diagnostic marker in between); transition position is not resolved within this interval; Adjacent to a single-marker segment
A3N03	CM046777.1	2G	1G	50,289,549	52,009,449	1,719,900	1,145,611	0	15	1	No	Adjacent to a single-marker segment
A3N03	CM046777.1	1G	0G	52,009,449	62,070,141	10,060,692	0	6	1	3	Yes	Transition interval spans a marker gap > 2 Mb (no diagnostic marker in

												between); transition position is not resolved within this interval; Adjacent to a single-marker segment
A3N03	CM046777.1	0G	1G	62,070,147	77,286,484	15,216,337	6	0	3	1	Yes	Transition interval spans a marker gap > 2 Mb (no diagnostic marker in between); transition position is not resolved within this interval; Adjacent to a single-marker segment
A3N03	CM046778.1	0G	2G	10,204,291	10,328,364	124,073	10,031,223	17,938	78	4	No	Flanking segments supported by 3-4 markers
A3N03	CM046778.1	2G	0G	10,346,302	10,480,661	134,359	17,938	33,489	4	2	No	Adjacent to a segment supported by < 3 markers
A3N03	CM046778.1	0G	2G	10,514,150	10,548,313	34,163	33,489	0	2	1	No	Adjacent to a single-

												marker segment
A3N03	CM046778.1	2G	0G	10,548,313	10,563,467	15,154	0	10,239,278	1	208	No	Adjacent to a single-marker segment
A3N03	CM046778.1	0G	1G	20,802,745	25,834,991	5,032,246	10,239,278	6,808,677	208	20	Yes	Transition interval spans a marker gap > 2 Mb (no diagnostic marker in between); transition position is not resolved within this interval; Both flanking segments supported by >= 5 markers
A3N03	CM046778.1	1G	2G	32,643,668	32,848,638	204,970	6,808,677	1,190,519	20	4	No	Flanking segments supported by 3-4 markers
A3N03	CM046778.1	2G	1G	34,039,157	34,095,194	56,037	1,190,519	3,555,163	4	37	No	Flanking segments supported by 3-4 markers
A3N03	CM046778.1	1G	2G	37,650,357	40,221,487	2,571,130	3,555,163	11,289,739	37	50	Yes	Transition interval spans a marker gap > 2 Mb (no

												diagnostic marker in between); transition position is not resolved within this interval; Both flanking segments supported by >= 5 markers
A3N03	CM046778.1	2G	1G	51,511,226	52,065,882	554,656	11,289,739	595,779	50	6	No	Both flanking segments supported by >= 5 markers
A3N03	CM046778.1	1G	2G	52,661,661	52,681,490	19,829	595,779	2,376,191	6	39	No	Both flanking segments supported by >= 5 markers
A3N03	CM046778.1	2G	0G	55,057,681	55,073,594	15,913	2,376,191	5856	39	10	No	Both flanking segments supported by >= 5 markers
A3N03	CM046778.1	0G	2G	55,079,450	55,118,360	38,910	5856	2,211,385	10	21	No	Both flanking segments supported by >= 5 markers
A3N03	CM046779.1	0G	2G	19,036,812	20,026,219	989,407	10,841,093	0	53	1	No	Adjacent to a single-marker segment

A3N03	CM046779.1	2G	0G	20,026,219	20,097,827	71,608	0	575,987	1	20	No	Adjacent to a single-marker segment
A3N03	CM046779.1	0G	2G	20,673,814	20,744,621	70,807	575,987	547,115	20	10	No	Both flanking segments supported by >= 5 markers
A3N03	CM046779.1	2G	0G	21,291,736	21,788,168	496,432	547,115	2,534,064	10	43	No	Both flanking segments supported by >= 5 markers
A3N03	CM046779.1	0G	2G	24,322,232	24,330,165	7933	2,534,064	0	43	1	No	Adjacent to a single-marker segment
A3N03	CM046779.1	2G	1G	24,330,165	24,359,606	29,441	0	1,460,565	1	3	No	Adjacent to a single-marker segment
A3N03	CM046779.1	1G	2G	25,820,171	27,150,560	1,330,389	1,460,565	408,227	3	11	No	Flanking segments supported by 3-4 markers
A3N03	CM046779.1	2G	0G	27,558,787	28,259,428	700,641	408,227	378,840	11	12	No	Both flanking segments supported by >= 5 markers
A3N03	CM046779.1	0G	2G	28,638,268	28,656,558	18,290	378,840	8269	12	4	No	Flanking segments supported by 3-4 markers
A3N03	CM046779.1	2G	1G	28,664,827	28,859,774	194,947	8269	1,210,622	4	16	No	Flanking segments supported

												by 3-4 markers
A3N03	CM046779.1	1G	2G	30,070,396	30,075,652	5256	1,210,622	39	16	3	No	Flanking segments supported by 3-4 markers
A3N03	CM046779.1	2G	1G	30,075,691	30,107,141	31,450	39	4,472,438	3	58	No	Flanking segments supported by 3-4 markers
A3N03	CM046779.1	1G	2G	34,579,579	34,595,703	16,124	4,472,438	1,208,959	58	13	No	Both flanking segments supported by >= 5 markers
A3N03	CM046779.1	2G	0G	35,804,662	35,806,590	1928	1,208,959	0	13	1	No	Adjacent to a single-marker segment
A3N03	CM046779.1	0G	2G	35,806,590	35,993,692	187,102	0	2,958,206	1	51	No	Adjacent to a single-marker segment
A3N03	CM046779.1	2G	1G	38,951,898	39,221,179	269,281	2,958,206	449,558	51	14	No	Both flanking segments supported by >= 5 markers
A3N03	CM046779.1	1G	2G	39,670,737	41,696,128	2,025,391	449,558	3,958,460	14	5	Yes	Transition interval spans a marker gap > 2 Mb (no diagnostic marker in between); transition position is

												not resolved within this interval; Both flanking segments supported by >= 5 markers
A3N03	CM046779.1	2G	1G	45,654,588	45,687,692	33,104	3,958,460	2,465,557	5	16	No	Both flanking segments supported by >= 5 markers
A3N03	CM046779.1	1G	2G	48,153,249	49,013,480	860,231	2,465,557	833,594	16	7	No	Both flanking segments supported by >= 5 markers
A3N03	CM046779.1	2G	0G	49,847,074	50,217,134	370,060	833,594	115,465	7	2	No	Adjacent to a segment supported by < 3 markers
A3N03	CM046780.1	0G	1G	43,763,399	45,140,740	1,377,341	32,896,083	1215	19	2	No	Adjacent to a segment supported by < 3 markers
A3N03	CM046780.1	1G	0G	45,141,955	45,492,684	350,729	1215	10,251,787	2	21	No	Adjacent to a segment supported by < 3 markers
A3N03	CM046781.1	0G	1G	7,957,294	7,959,205	1911	7,267,052	53	60	3	No	Flanking segments supported

												by 3-4 markers
A3N03	CM046781.1	1G	0G	7,959,258	7,982,745	23,487	53	3,944,709	3	166	No	Flanking segments supported by 3-4 markers
A3N03	CM046781.1	0G	1G	11,927,454	11,928,631	1177	3,944,709	9188	166	8	No	Both flanking segments supported by >= 5 markers
A3N03	CM046781.1	1G	0G	11,937,819	11,966,078	28,259	9188	29,958,040	8	270	No	Both flanking segments supported by >= 5 markers
A3N03	CM046781.1	0G	1G	41,924,118	44,038,805	2,114,687	29,958,040	9	270	3	Yes	Transition interval spans a marker gap > 2 Mb (no diagnostic marker in between); transition position is not resolved within this interval; Flanking segments supported by 3-4 markers
A3N03	CM046781.1	1G	0G	44,038,814	44,348,511	309,697	9	2,896,430	3	31	No	Flanking segments supported by 3-4

												markers
A3N03	CM046781.1	0G	2G	47,244,941	49,087,949	1,843,008	2,896,430	0	31	1	No	Adjacent to a single-marker segment
A3N03	CM046781.1	2G	0G	49,087,949	49,840,459	752,510	0	2,680,383	1	5	No	Adjacent to a single-marker segment
A3N03	CM046781.1	0G	1G	52,520,842	52,520,848	6	2,680,383	1,622,404	5	30	No	Both flanking segments supported by >= 5 markers
A3N03	CM046781.1	1G	0G	54,143,252	54,429,375	286,123	1,622,404	5,430,748	30	61	No	Both flanking segments supported by >= 5 markers
A3N03	CM046782.1	1G	2G	7,919,342	7,957,603	38,261	6,201,335	16,078	65	2	No	Adjacent to a segment supported by < 3 markers
A3N03	CM046782.1	2G	1G	7,973,681	8,232,127	258,446	16,078	1,773,107	2	5	No	Adjacent to a segment supported by < 3 markers
A3N03	CM046782.1	1G	2G	10,005,234	10,007,096	1862	1,773,107	168,696	5	5	No	Both flanking segments supported by >= 5 markers
A3N03	CM046782.1	2G	1G	10,175,792	10,178,812	3020	168,696	335,087	5	17	No	Both flanking segments

												supported by >= 5 markers
A3N03	CM046782.1	1G	2G	10,513,899	10,779,154	265,255	335,087	0	17	1	No	Adjacent to a single-marker segment
A3N03	CM046782.1	2G	0G	10,779,154	11,081,665	302,511	0	1,056,850	1	5	No	Adjacent to a single-marker segment
A3N03	CM046782.1	0G	2G	12,138,515	12,188,895	50,380	1,056,850	70	5	3	No	Flanking segments supported by 3-4 markers
A3N03	CM046782.1	2G	1G	12,188,965	12,189,247	282	70	5,392,729	3	65	No	Flanking segments supported by 3-4 markers
A3N03	CM046782.1	1G	0G	17,581,976	17,588,232	6256	5,392,729	29,153	65	11	No	Both flanking segments supported by >= 5 markers
A3N03	CM046782.1	0G	1G	17,617,385	18,062,307	444,922	29,153	8,423,572	11	30	No	Both flanking segments supported by >= 5 markers
A3N03	CM046782.1	1G	0G	26,485,879	26,486,270	391	8,423,572	3,626,538	30	7	No	Both flanking segments supported by >= 5 markers
A3N03	CM046782.1	0G	2G	30,112,808	44,756,538	14,643,730	3,626,538	1,281,281	7	4	Yes	Transition interval spans a marker

												gap > 2 Mb (no diagnostic marker in between); transition position is not resolved within this interval; Flanking segments supported by 3-4 markers
A3N03	CM046782.1	2G	1G	46,037,819	51,088,142	5,050,323	1,281,281	1,223,069	4	4	Yes	Transition interval spans a marker gap > 2 Mb (no diagnostic marker in between); transition position is not resolved within this interval; Flanking segments supported by 3-4 markers
A3N03	CM046782.1	1G	0G	52,311,211	52,609,396	298,185	1,223,069	887,840	4	10	No	Flanking segments supported by 3-4 markers
A3N03	CM046782.1	0G	2G	53,497,236	54,100,172	602,936	887,840	0	10	1	No	Adjacent to a single-

												marker segment
A3N03	CM046782.1	2G	0G	54,100,172	54,682,961	582,789	0	914,037	1	11	No	Adjacent to a single-marker segment
A3N03	CM046782.1	0G	1G	55,596,998	55,645,071	48,073	914,037	116,787	11	3	No	Flanking segments supported by 3-4 markers
A3N03	CM046782.1	1G	0G	55,761,858	55,784,443	22,585	116,787	859,032	3	11	No	Flanking segments supported by 3-4 markers
A3N03	CM046782.1	0G	2G	56,643,475	56,763,405	119,930	859,032	0	11	1	No	Adjacent to a single-marker segment
A3N03	CM046782.1	2G	0G	56,763,405	56,872,230	108,825	0	57,985	1	9	No	Adjacent to a single-marker segment
A3N03	CM046782.1	0G	2G	56,930,215	56,965,435	35,220	57,985	19,366	9	2	No	Adjacent to a segment supported by < 3 markers
A3N03	CM046782.1	2G	0G	56,984,801	56,992,017	7216	19,366	23,107	2	10	No	Adjacent to a segment supported by < 3 markers
A3N03	CM046782.1	0G	2G	57,015,124	57,255,126	240,002	23,107	41,304	10	5	No	Both flanking segments supported by >= 5 markers

A3N03	CM046782.1	2G	1G	57,296,430	57,505,910	209,480	41,304	2,466,567	5	13	No	Both flanking segments supported by >= 5 markers
A3N03	CM046783.1	0G	1G	2,504,850	5,763,011	3,258,161	2,475,201	13,703,123	6	32	Yes	Transition interval spans a marker gap > 2 Mb (no diagnostic marker in between); transition position is not resolved within this interval; Both flanking segments supported by >= 5 markers
A3N03	CM046783.1	1G	2G	19,466,134	20,054,493	588,359	13,703,123	1	32	2	No	Adjacent to a segment supported by < 3 markers
A3N03	CM046783.1	2G	1G	20,054,494	30,825,912	10,771,418	1	191,179	2	2	Yes	Transition interval spans a marker gap > 2 Mb (no diagnostic marker in between); transition

												position is not resolved within this interval; Adjacent to a segment supported by < 3 markers
A3N03	CM046783.1	1G	2G	31,017,091	34,982,859	3,965,768	191,179	16,686,975	2	29	Yes	Transition interval spans a marker gap > 2 Mb (no diagnostic marker in between); transition position is not resolved within this interval; Adjacent to a segment supported by < 3 markers
A3N03	CM046783.1	2G	1G	51,669,834	52,429,617	759,783	16,686,975	1558	29	3	No	Flanking segments supported by 3-4 markers
A3N03	CM046784.1	0G	1G	3,868,587	4,122,539	253,952	157,065	4,793,506	4	16	No	Flanking segments supported by 3-4 markers

A3N03	CM046784.1	1G	0G	8,916,045	9,552,632	636,587	4,793,506	529,405	16	6	No	Both flanking segments supported by >= 5 markers
A3N03	CM046784.1	0G	1G	10,082,037	10,248,604	166,567	529,405	2677	6	2	No	Adjacent to a segment supported by < 3 markers
A3N03	CM046784.1	1G	2G	10,251,281	14,640,883	4,389,602	2677	1,147,410	2	3	Yes	Transition interval spans a marker gap > 2 Mb (no diagnostic marker in between); transition position is not resolved within this interval; Adjacent to a segment supported by < 3 markers
A3N03	CM046784.1	2G	0G	15,788,293	16,190,229	401,936	1,147,410	8,013,700	3	15	No	Flanking segments supported by 3-4 markers
A3N03	CM046784.1	0G	1G	24,203,929	31,280,007	7,076,078	8,013,700	4,289,757	15	10	Yes	Transition interval spans a marker gap > 2

												Mb (no diagnostic marker in between); transition position is not resolved within this interval; Both flanking segments supported by >= 5 markers
A3N03	CM046784.1	1G	0G	35,569,764	35,901,528	331,764	4,289,757	222,573	10	21	No	Both flanking segments supported by >= 5 markers
A3N03	CM046784.1	0G	1G	36,124,101	36,125,808	1707	222,573	330,897	21	18	No	Both flanking segments supported by >= 5 markers
A3N03	CM046785.1	0G	1G	3,469,616	3,469,689	73	1,246,611	4,498,349	4	6	No	Flanking segments supported by 3-4 markers
A3N03	CM046785.1	1G	0G	7,968,038	8,482,686	514,648	4,498,349	2,450,349	6	15	No	Both flanking segments supported by >= 5 markers
A3N03	CM046785.1	0G	1G	10,933,035	11,948,890	1,015,855	2,450,349	1,461,348	15	17	No	Both flanking segments supported

												by >= 5 markers
A3N03	CM046785.1	1G	0G	13,410,238	13,730,551	320,313	1,461,348	1,905,122	17	62	No	Both flanking segments supported by >= 5 markers
A3N03	CM046785.1	0G	1G	15,635,673	15,651,738	16,065	1,905,122	403	62	4	No	Flanking segments supported by 3-4 markers
A3N03	CM046785.1	1G	0G	15,652,141	15,653,205	1064	403	3,924,417	4	58	No	Flanking segments supported by 3-4 markers
A3N03	CM046785.1	0G	2G	19,577,622	19,836,574	258,952	3,924,417	0	58	1	No	Adjacent to a single-marker segment
A3N03	CM046785.1	2G	0G	19,836,574	20,045,990	209,416	0	1,794,739	1	47	No	Adjacent to a single-marker segment
A3N03	CM046785.1	0G	1G	21,840,729	22,212,038	371,309	1,794,739	1,925,454	47	8	No	Both flanking segments supported by >= 5 markers
A3N03	CM046785.1	1G	2G	24,137,492	25,308,597	1,171,105	1,925,454	4217	8	3	No	Flanking segments supported by 3-4 markers
A3N03	CM046785.1	2G	1G	25,312,814	25,729,710	416,896	4217	2,614,296	3	8	No	Flanking segments supported by 3-4 markers

A3N03	CM046785.1	1G	2G	28,344,006	28,345,743	1737	2,614,296	278,531	8	5	No	Both flanking segments supported by >= 5 markers
A3N03	CM046785.1	2G	1G	28,624,274	29,018,798	394,524	278,531	22,481,213	5	91	No	Both flanking segments supported by >= 5 markers
A3N04	CM046776.1	1G	0G	9,310,261	9,311,174	913	5,795,891	47	30	6	No	Both flanking segments supported by >= 5 markers
A3N04	CM046776.1	0G	1G	9,311,221	9,311,718	497	47	653,221	6	25	No	Both flanking segments supported by >= 5 markers
A3N04	CM046776.1	1G	0G	9,964,939	10,809,600	844,661	653,221	312,906	25	15	No	Both flanking segments supported by >= 5 markers
A3N04	CM046776.1	0G	1G	11,122,506	11,171,205	48,699	312,906	4,156,766	15	61	No	Both flanking segments supported by >= 5 markers
A3N04	CM046776.1	1G	2G	15,327,971	15,436,540	108,569	4,156,766	29,084	61	4	No	Flanking segments supported by 3-4 markers
A3N04	CM046776.1	2G	1G	15,465,624	15,484,658	19,034	29,084	14,870,892	4	92	No	Flanking segments

												supported by 3-4 markers
A3N04	CM046776.1	1G	0G	30,355,550	30,474,620	119,070	14,870,892	6,042,143	92	9	No	Both flanking segments supported by >= 5 markers
A3N04	CM046777.1	2G	0G	14,801,378	30,212,173	15,410,795	11,645,136	0	4	1	Yes	Transition interval spans a marker gap > 2 Mb (no diagnostic marker in between); transition position is not resolved within this interval; Adjacent to a single-marker segment
A3N04	CM046777.1	0G	2G	30,212,173	49,058,732	18,846,559	0	84,415	1	5	Yes	Transition interval spans a marker gap > 2 Mb (no diagnostic marker in between); transition position is not resolved within this interval;

A3N04	CM046777.1	2G	1G	49,143,147	49,143,938	791	84,415	12,926,209	5	28	No	Adjacent to a single-marker segment Both flanking segments supported by >= 5 markers
A3N04	CM046777.1	1G	2G	62,070,147	73,627,875	11,557,728	12,926,209	3,658,609	28	2	Yes	Transition interval spans a marker gap > 2 Mb (no diagnostic marker in between); transition position is not resolved within this interval; Adjacent to a segment supported by < 3 markers
A3N04	CM046778.1	0G	1G	3,059,958	3,193,249	133,291	2,886,890	1225	15	5	No	Both flanking segments supported by >= 5 markers
A3N04	CM046778.1	1G	0G	3,194,474	3,556,449	361,975	1225	706,967	5	9	No	Both flanking segments supported by >= 5 markers

A3N04	CM046778.1	0G	1G	4,263,416	5,405,961	1,142,545	706,967	0	9	1	No	Adjacent to a single-marker segment
A3N04	CM046778.1	1G	0G	5,405,961	5,511,705	105,744	0	62,800	1	5	No	Adjacent to a single-marker segment
A3N04	CM046778.1	0G	2G	5,574,505	5,724,117	149,612	62,800	0	5	1	No	Adjacent to a single-marker segment
A3N04	CM046778.1	2G	1G	5,724,117	5,907,749	183,632	0	58,719	1	4	No	Adjacent to a single-marker segment
A3N04	CM046778.1	1G	0G	5,966,468	5,966,568	100	58,719	2,564,271	4	23	No	Flanking segments supported by 3-4 markers
A3N04	CM046778.1	0G	2G	8,530,839	8,530,919	80	2,564,271	0	23	1	No	Adjacent to a single-marker segment
A3N04	CM046778.1	2G	0G	8,530,919	8,532,969	2050	0	10,641,377	1	220	No	Adjacent to a single-marker segment
A3N04	CM046778.1	0G	2G	19,174,346	19,263,509	89,163	10,641,377	0	220	1	No	Adjacent to a single-marker segment
A3N04	CM046778.1	2G	0G	19,263,509	19,574,548	311,039	0	818,441	1	19	No	Adjacent to a single-marker segment
A3N04	CM046778.1	0G	1G	20,392,989	20,614,405	221,416	818,441	672,669	19	5	No	Both flanking segments supported by >= 5 markers

A3N04	CM046778.1	1G	0G	21,287,074	23,392,007	2,104,933	672,669	8,383,908	5	24	Yes	Transition interval spans a marker gap > 2 Mb (no diagnostic marker in between); transition position is not resolved within this interval; Both flanking segments supported by >= 5 markers
A3N04	CM046778.1	0G	1G	31,775,915	32,442,787	666,872	8,383,908	405,851	24	5	No	Both flanking segments supported by >= 5 markers
A3N04	CM046778.1	1G	2G	32,848,638	33,804,817	956,179	405,851	234,340	5	3	No	Flanking segments supported by 3-4 markers
A3N04	CM046778.1	2G	1G	34,039,157	34,095,194	56,037	234,340	38,572	3	6	No	Flanking segments supported by 3-4 markers
A3N04	CM046778.1	1G	0G	34,133,766	34,171,319	37,553	38,572	540,753	6	33	No	Both flanking segments supported by >= 5 markers

A3N04	CM046778.1	0G	2G	34,712,072	34,985,070	272,998	540,753	101,797	33	2	No	Adjacent to a segment supported by < 3 markers
A3N04	CM046778.1	2G	1G	35,086,867	35,550,734	463,867	101,797	2,099,623	2	7	No	Adjacent to a segment supported by < 3 markers
A3N04	CM046778.1	1G	0G	37,650,357	38,039,732	389,375	2,099,623	6,139,855	7	37	No	Both flanking segments supported by >= 5 markers
A3N04	CM046778.1	0G	2G	44,179,587	44,433,795	254,208	6,139,855	7	37	3	No	Flanking segments supported by 3-4 markers
A3N04	CM046778.1	2G	0G	44,433,802	44,457,664	23,862	7	8,223,826	3	77	No	Flanking segments supported by 3-4 markers
A3N04	CM046778.1	0G	1G	52,681,490	52,681,543	53	8,223,826	30,310	77	5	No	Both flanking segments supported by >= 5 markers
A3N04	CM046778.1	1G	0G	52,711,853	52,918,679	206,826	30,310	4,411,179	5	119	No	Both flanking segments supported by >= 5 markers

A3N04	CM046779.1	1G	2G	8,646,804	17,515,790	8,868,986	404,469	1,521,022	24	19	Yes	Transition interval spans a marker gap > 2 Mb (no diagnostic marker in between); transition position is not resolved within this interval; Both flanking segments supported by >= 5 markers
A3N04	CM046779.1	2G	1G	19,036,812	20,026,219	989,407	1,521,022	151,820	19	3	No	Flanking segments supported by 3-4 markers
A3N04	CM046779.1	1G	2G	20,178,039	20,393,591	215,552	151,820	1,417,850	3	12	No	Flanking segments supported by 3-4 markers
A3N04	CM046779.1	2G	1G	21,811,441	23,990,055	2,178,614	1,417,850	23,992	12	6	Yes	Transition interval spans a marker gap > 2 Mb (no diagnostic marker in between); transition position is not

												resolved within this interval; Both flanking segments supported by >= 5 markers
A3N04	CM046779.1	1G	2G	24,014,047	24,072,465	58,418	23,992	257,700	6	15	No	Both flanking segments supported by >= 5 markers
A3N04	CM046779.1	2G	0G	24,330,165	24,945,762	615,597	257,700	21,544	15	2	No	Adjacent to a segment supported by < 3 markers
A3N04	CM046779.1	0G	2G	24,967,306	25,661,464	694,158	21,544	4,352,391	2	26	No	Adjacent to a segment supported by < 3 markers
A3N04	CM046779.1	2G	1G	30,013,855	30,050,811	36,956	4,352,391	56,922	26	10	No	Both flanking segments supported by >= 5 markers
A3N04	CM046779.1	1G	2G	30,107,733	30,651,756	544,023	56,922	2,154,978	10	22	No	Both flanking segments supported by >= 5 markers
A3N04	CM046779.1	2G	1G	32,806,734	32,806,879	145	2,154,978	12,630	22	7	No	Both flanking segments supported

												by >= 5 markers
A3N04	CM046779.1	1G	2G	32,819,509	32,887,847	68,338	12,630	4,829,078	7	63	No	Both flanking segments supported by >= 5 markers
A3N04	CM046779.1	2G	1G	37,716,925	37,717,123	198	4,829,078	11,298,782	63	68	No	Both flanking segments supported by >= 5 markers
A3N04	CM046779.1	1G	2G	49,015,905	49,083,608	67,703	11,298,782	360,219	68	10	No	Both flanking segments supported by >= 5 markers
A3N04	CM046779.1	2G	1G	49,443,827	49,864,958	421,131	360,219	352,176	10	4	No	Flanking segments supported by 3-4 markers
A3N04	CM046781.1	2G	0G	3,004,376	4,609,424	1,605,048	2,314,134	731,115	10	3	No	Flanking segments supported by 3-4 markers
A3N04	CM046781.1	0G	1G	5,340,539	6,188,285	847,746	731,115	1,633,167	3	26	No	Flanking segments supported by 3-4 markers
A3N04	CM046781.1	1G	2G	7,821,452	7,826,918	5466	1,633,167	132,314	26	9	No	Both flanking segments supported by >= 5 markers
A3N04	CM046781.1	2G	1G	7,959,232	7,982,745	23,513	132,314	3,790,804	9	114	No	Both flanking

												segments supported by >= 5 markers
A3N04	CM046781.1	1G	2G	11,773,549	11,927,085	153,536	3,790,804	10,734	114	16	No	Both flanking segments supported by >= 5 markers
A3N04	CM046781.1	2G	1G	11,937,819	11,966,078	28,259	10,734	2,631,793	16	56	No	Both flanking segments supported by >= 5 markers
A3N04	CM046781.1	1G	0G	14,597,871	14,605,707	7836	2,631,793	65	56	9	No	Both flanking segments supported by >= 5 markers
A3N04	CM046781.1	0G	1G	14,605,772	14,607,975	2203	65	318,775	9	17	No	Both flanking segments supported by >= 5 markers
A3N04	CM046781.1	1G	0G	14,926,750	15,135,492	208,742	318,775	158,892	17	3	No	Flanking segments supported by 3-4 markers
A3N04	CM046781.1	0G	2G	15,294,384	15,312,760	18,376	158,892	1339	3	3	No	Flanking segments supported by 3-4 markers
A3N04	CM046781.1	2G	1G	15,314,099	15,376,909	62,810	1339	3,281,872	3	98	No	Flanking segments supported by 3-4 markers

A3N04	CM046781.1	1G	0G	18,658,781	41,370,140	22,711,359	3,281,872	3,096,687	98	6	Yes	Transition interval spans a marker gap > 2 Mb (no diagnostic marker in between); transition position is not resolved within this interval; Both flanking segments supported by >= 5 markers
A3N04	CM046781.1	0G	2G	44,466,827	44,510,413	43,586	3,096,687	0	6	1	No	Adjacent to a single-marker segment
A3N04	CM046781.1	2G	0G	44,510,413	44,700,060	189,647	0	1,263,735	1	23	No	Adjacent to a single-marker segment
A3N04	CM046781.1	0G	1G	45,963,795	46,055,187	91,392	1,263,735	3,866,536	23	3	No	Flanking segments supported by 3-4 markers
A3N04	CM046781.1	1G	0G	49,921,723	52,520,842	2,599,119	3,866,536	4,328,571	3	88	Yes	Transition interval spans a marker gap > 2 Mb (no diagnostic marker in between);

												transition position is not resolved within this interval; Flanking segments supported by 3-4 markers
A3N04	CM046781.1	0G	1G	56,849,413	58,733,593	1,884,180	4,328,571	0	88	1	No	Adjacent to a single-marker segment
A3N04	CM046781.1	1G	0G	58,733,593	59,259,253	525,660	0	1,928,221	1	15	No	Adjacent to a single-marker segment
A3N04	CM046782.1	2G	1G	1,149,249	1,177,276	28,027	1854	887,363	4	7	No	Flanking segments supported by 3-4 markers
A3N04	CM046782.1	1G	0G	2,064,639	2,568,508	503,869	887,363	54	7	2	No	Adjacent to a segment supported by < 3 markers
A3N04	CM046782.1	0G	2G	2,568,562	2,847,295	278,733	54	8273	2	5	No	Adjacent to a segment supported by < 3 markers
A3N04	CM046782.1	2G	1G	2,855,568	3,163,050	307,482	8273	11,684,973	5	127	No	Both flanking segments supported by >= 5 markers

A3N04	CM046782.1	1G	2G	14,848,023	15,090,020	241,997	11,684,973	52	127	2	No	Adjacent to a segment supported by < 3 markers
A3N04	CM046782.1	2G	1G	15,090,072	15,112,181	22,109	52	6,066,915	2	76	No	Adjacent to a segment supported by < 3 markers
A3N04	CM046782.1	1G	0G	21,179,096	22,109,034	929,938	6,066,915	6,493,868	76	12	No	Both flanking segments supported by >= 5 markers
A3N04	CM046782.1	0G	1G	28,602,902	44,756,538	16,153,636	6,493,868	7,557,693	12	6	Yes	Transition interval spans a marker gap > 2 Mb (no diagnostic marker in between); transition position is not resolved within this interval; Both flanking segments supported by >= 5 markers
A3N04	CM046782.1	1G	0G	52,314,231	53,479,092	1,164,861	7,557,693	2,117,906	6	23	No	Both flanking segments supported

												by >= 5 markers
A3N04	CM046782.1	0G	1G	55,596,998	55,645,071	48,073	2,117,906	70	23	4	No	Flanking segments supported by 3-4 markers
A3N04	CM046782.1	1G	0G	55,645,141	55,784,443	139,302	70	859,032	4	16	No	Flanking segments supported by 3-4 markers
A3N04	CM046782.1	0G	2G	56,643,475	56,761,948	118,473	859,032	1457	16	2	No	Adjacent to a segment supported by < 3 markers
A3N04	CM046782.1	2G	0G	56,763,405	56,872,230	108,825	1457	142,894	2	25	No	Adjacent to a segment supported by < 3 markers
A3N04	CM046782.1	0G	1G	57,015,124	57,255,126	240,002	142,894	250,784	25	4	No	Flanking segments supported by 3-4 markers
A3N04	CM046782.1	1G	0G	57,505,910	57,505,914	4	250,784	2,466,563	4	19	No	Flanking segments supported by 3-4 markers
A3N04	CM046783.1	0G	2G	7,616,950	9,236,876	1,619,926	7,587,301	0	19	1	No	Adjacent to a single-marker segment
A3N04	CM046783.1	2G	0G	9,236,876	9,925,782	688,906	0	3,511,104	1	23	No	Adjacent to a single-marker segment

A3N04	CM046783.1	0G	1G	13,436,886	17,965,393	4,528,507	3,511,104	1,031,684	23	3	Yes	Transition interval spans a marker gap > 2 Mb (no diagnostic marker in between); transition position is not resolved within this interval; Flanking segments supported by 3-4 markers
A3N04	CM046783.1	1G	0G	18,997,077	19,466,134	469,057	1,031,684	2,037,378	3	11	No	Flanking segments supported by 3-4 markers
A3N04	CM046783.1	0G	1G	21,503,512	25,993,580	4,490,068	2,037,378	1,208,372	11	3	Yes	Transition interval spans a marker gap > 2 Mb (no diagnostic marker in between); transition position is not resolved within this interval; Flanking segments supported

												by 3-4 markers
A3N04	CM046783.1	1G	0G	27,201,952	30,825,888	3,623,936	1,208,372	191,228	3	5	Yes	Transition interval spans a marker gap > 2 Mb (no diagnostic marker in between); transition position is not resolved within this interval; Flanking segments supported by 3-4 markers
A3N04	CM046783.1	0G	2G	31,017,116	34,982,859	3,965,743	191,228	0	5	1	Yes	Transition interval spans a marker gap > 2 Mb (no diagnostic marker in between); transition position is not resolved within this interval; Adjacent to a single-marker segment

A3N04	CM046783.1	2G	0G	34,982,859	35,291,317	308,458	0	2,669,074	1	17	No	Adjacent to a single-marker segment
A3N04	CM046783.1	0G	1G	37,960,391	45,741,541	7,781,150	2,669,074	5,928,293	17	16	Yes	Transition interval spans a marker gap > 2 Mb (no diagnostic marker in between); transition position is not resolved within this interval; Both flanking segments supported by >= 5 markers
A3N04	CM046783.1	1G	0G	51,669,834	52,426,413	756,579	5,928,293	4762	16	7	No	Both flanking segments supported by >= 5 markers
A3N04	CM046784.1	0G	1G	3,868,587	4,122,539	253,952	157,065	4,310,694	4	15	No	Flanking segments supported by 3-4 markers
A3N04	CM046784.1	1G	2G	8,433,233	8,916,045	482,812	4,310,694	638,932	15	2	No	Adjacent to a segment supported by < 3 markers

A3N04	CM046784.1	2G	1G	9,554,977	10,082,037	527,060	638,932	4,558,846	2	9	No	Adjacent to a segment supported by < 3 markers
A3N04	CM046784.1	1G	2G	14,640,883	15,788,290	1,147,407	4,558,846	3	9	2	No	Adjacent to a segment supported by < 3 markers
A3N04	CM046784.1	2G	1G	15,788,293	16,190,229	401,936	3	18,799,309	2	26	No	Adjacent to a segment supported by < 3 markers
A3N04	CM046784.1	1G	0G	34,989,538	35,002,383	12,845	18,799,309	1,454,322	26	77	No	Both flanking segments supported by >= 5 markers
A3N04	CM046785.1	2G	1G	3,469,713	4,785,560	1,315,847	1,246,708	8,055,738	3	22	No	Flanking segments supported by 3-4 markers
A3N04	CM046785.1	1G	2G	12,841,298	13,384,953	543,655	8,055,738	915,974	22	23	No	Both flanking segments supported by >= 5 markers
A3N04	CM046785.1	2G	1G	14,300,927	14,301,523	596	915,974	153,904	23	4	No	Flanking segments supported by 3-4 markers
A3N04	CM046785.1	1G	2G	14,455,427	14,514,750	59,323	153,904	5,531,240	4	53	No	Flanking segments supported

												by 3-4 markers
A3N04	CM046785.1	2G	1G	20,045,990	20,459,911	413,921	5,531,240	951,194	53	13	No	Both flanking segments supported by >= 5 markers
A3N04	CM046785.1	1G	2G	21,411,105	21,489,441	78,336	951,194	8,569,631	13	35	No	Both flanking segments supported by >= 5 markers
A3N04	CM046785.1	2G	1G	30,059,072	30,094,675	35,603	8,569,631	5,227,684	35	19	No	Both flanking segments supported by >= 5 markers
A3N04	CM046785.1	1G	2G	35,322,359	38,681,683	3,359,324	5,227,684	1,461,865	19	11	Yes	Transition interval spans a marker gap > 2 Mb (no diagnostic marker in between); transition position is not resolved within this interval; Both flanking segments supported by >= 5 markers
A3N04	CM046785.1	2G	1G	40,143,548	41,807,005	1,663,457	1,461,865	6,373,737	11	44	No	Both flanking

												segments supported by >= 5 markers
A3N04	CM046785.1	1G	2G	48,180,742	49,913,169	1,732,427	6,373,737	220,584	44	2	No	Adjacent to a segment supported by < 3 markers
A3N04	CM046785.1	2G	1G	50,133,753	51,422,252	1,288,499	220,584	4,411,427	2	4	No	Adjacent to a segment supported by < 3 markers
A3N05	CM046776.1	0G	2G	5,813,347	5,861,938	48,591	0	3,449,028	1	23	No	Adjacent to a single-marker segment
A3N05	CM046776.1	2G	1G	9,310,966	9,311,174	208	3,449,028	47	23	6	No	Both flanking segments supported by >= 5 markers
A3N05	CM046776.1	1G	2G	9,311,221	9,311,718	497	47	6,335,893	6	81	No	Both flanking segments supported by >= 5 markers
A3N05	CM046776.1	2G	1G	15,647,611	16,578,873	931,262	6,335,893	1,079,638	81	12	No	Both flanking segments supported by >= 5 markers
A3N05	CM046776.1	1G	2G	17,658,511	17,672,021	13,510	1,079,638	24,479	12	3	No	Flanking segments supported by 3-4 markers

A3N05	CM046776.1	2G	1G	17,696,500	17,701,122	4622	24,479	11,890,125	3	56	No	Flanking segments supported by 3-4 markers
A3N05	CM046776.1	1G	2G	29,591,247	30,355,483	764,236	11,890,125	119,137	56	7	No	Both flanking segments supported by >= 5 markers
A3N05	CM046776.1	2G	1G	30,474,620	30,489,991	15,371	119,137	147,880	7	5	No	Both flanking segments supported by >= 5 markers
A3N05	CM046777.1	1G	0G	3,156,242	14,623,210	11,466,968	0	15,588,963	1	5	Yes	Transition interval spans a marker gap > 2 Mb (no diagnostic marker in between); transition position is not resolved within this interval; Adjacent to a single-marker segment
A3N05	CM046777.1	0G	1G	30,212,173	38,511,280	8,299,107	15,588,963	0	5	1	Yes	Transition interval spans a marker gap > 2 Mb (no diagnostic

												marker in between); transition position is not resolved within this interval; Adjacent to a single-marker segment
A3N05	CM046777.1	1G	2G	38,511,280	49,143,088	10,631,808	0	490,109	1	19	Yes	Transition interval spans a marker gap > 2 Mb (no diagnostic marker in between); transition position is not resolved within this interval; Adjacent to a single-marker segment
A3N05	CM046777.1	2G	1G	49,633,197	49,652,578	19,381	490,109	2,356,871	19	4	No	Flanking segments supported by 3-4 markers
A3N05	CM046777.1	1G	0G	52,009,449	62,070,141	10,060,692	2,356,871	11,557,734	4	4	Yes	Transition interval spans a marker gap > 2 Mb (no diagnostic

												marker in between); transition position is not resolved within this interval; Flanking segments supported by 3-4 markers
A3N05	CM046778.1	0G	1G	5,574,254	5,907,751	333,497	5,401,186	58,848	36	5	No	Both flanking segments supported by >= 5 markers
A3N05	CM046778.1	1G	0G	5,966,599	6,076,091	109,492	58,848	1,524,635	5	15	No	Both flanking segments supported by >= 5 markers
A3N05	CM046778.1	0G	1G	7,600,726	8,418,170	817,444	1,524,635	1,785,588	15	17	No	Both flanking segments supported by >= 5 markers
A3N05	CM046778.1	1G	0G	10,203,758	10,298,377	94,619	1,785,588	34,981	17	5	No	Both flanking segments supported by >= 5 markers
A3N05	CM046778.1	0G	1G	10,333,358	10,480,661	147,303	34,981	23,653,105	5	191	No	Both flanking segments supported by >= 5 markers

A3N05	CM046778.1	1G	2G	34,133,766	34,168,568	34,802	23,653,105	9636	191	5	No	Both flanking segments supported by >= 5 markers
A3N05	CM046778.1	2G	0G	34,178,204	34,191,882	13,678	9636	213	5	6	No	Both flanking segments supported by >= 5 markers
A3N05	CM046778.1	0G	1G	34,192,095	34,195,641	3546	213	789,429	6	11	No	Both flanking segments supported by >= 5 markers
A3N05	CM046778.1	1G	2G	34,985,070	36,307,130	1,322,060	789,429	46	11	2	No	Adjacent to a segment supported by < 3 markers
A3N05	CM046778.1	2G	0G	36,307,176	36,536,647	229,471	46	32	2	3	No	Adjacent to a segment supported by < 3 markers
A3N05	CM046778.1	0G	1G	36,536,679	37,650,357	1,113,678	32	17,777,734	3	165	No	Flanking segments supported by 3-4 markers
A3N05	CM046778.1	1G	2G	55,428,091	55,431,559	3468	17,777,734	1,898,299	165	13	No	Both flanking segments supported by >= 5 markers
A3N05	CM046779.1	2G	1G	8,195,719	8,590,352	394,633	0	5821	1	4	No	Adjacent to a single-

												marker segment
A3N05	CM046779.1	1G	2G	8,596,173	8,599,878	3705	5821	46,926	4	10	No	Flanking segments supported by 3-4 markers
A3N05	CM046779.1	2G	1G	8,646,804	11,055,849	2,409,045	46,926	6,471,661	10	11	Yes	Transition interval spans a marker gap > 2 Mb (no diagnostic marker in between); transition position is not resolved within this interval; Both flanking segments supported by >= 5 markers
A3N05	CM046779.1	1G	2G	17,527,510	17,559,818	32,308	6,471,661	7,407,488	11	54	No	Both flanking segments supported by >= 5 markers
A3N05	CM046779.1	2G	1G	24,967,306	25,661,464	694,158	7,407,488	1,170,236	54	3	No	Flanking segments supported by 3-4 markers
A3N05	CM046779.1	1G	2G	26,831,700	27,150,560	318,860	1,170,236	207,615	3	4	No	Flanking segments supported by 3-4

												markers
A3N05	CM046779.1	2G	1G	27,358,175	27,558,747	200,572	207,615	1,078,361	4	12	No	Flanking segments supported by 3-4 markers
A3N05	CM046779.1	1G	2G	28,637,108	28,660,142	23,034	1,078,361	263,415	12	4	No	Flanking segments supported by 3-4 markers
A3N05	CM046779.1	2G	1G	28,923,557	29,192,185	268,628	263,415	142,600	4	7	No	Flanking segments supported by 3-4 markers
A3N05	CM046779.1	1G	2G	29,334,785	29,477,779	142,994	142,600	124	7	2	No	Adjacent to a segment supported by < 3 markers
A3N05	CM046779.1	2G	1G	29,477,903	30,050,811	572,908	124	2,641,718	2	24	No	Adjacent to a segment supported by < 3 markers
A3N05	CM046779.1	1G	2G	32,692,529	32,769,658	77,129	2,641,718	2198	24	3	No	Flanking segments supported by 3-4 markers
A3N05	CM046779.1	2G	0G	32,771,856	32,806,879	35,023	2198	12,630	3	10	No	Flanking segments supported by 3-4 markers
A3N05	CM046779.1	0G	1G	32,819,509	32,887,847	68,338	12,630	3,380,262	10	71	No	Both flanking segments supported

												by >= 5 markers
A3N05	CM046779.1	1G	0G	36,268,109	37,708,756	1,440,647	3,380,262	1,923,531	71	62	No	Both flanking segments supported by >= 5 markers
A3N05	CM046779.1	0G	2G	39,632,287	39,670,737	38,450	1,923,531	0	62	1	No	Adjacent to a single-marker segment
A3N05	CM046779.1	2G	0G	39,670,737	41,696,128	2,025,391	0	3,698,181	1	8	Yes	Transition interval spans a marker gap > 2 Mb (no diagnostic marker in between); transition position is not resolved within this interval; Adjacent to a single-marker segment
A3N05	CM046779.1	0G	1G	45,394,309	45,403,278	8969	3,698,181	659	8	7	No	Both flanking segments supported by >= 5 markers
A3N05	CM046779.1	1G	0G	45,403,937	45,687,692	283,755	659	3,408,262	7	28	No	Both flanking segments supported by >= 5 markers

A3N05	CM046779.1	0G	2G	49,095,954	49,443,827	347,873	3,408,262	0	28	1	No	Adjacent to a single-marker segment
A3N05	CM046779.1	2G	0G	49,443,827	49,490,662	46,835	0	841,937	1	7	No	Adjacent to a single-marker segment
A3N05	CM046781.1	2G	1G	1,376,697	1,910,072	533,375	122,838	4280	5	3	No	Flanking segments supported by 3-4 markers
A3N05	CM046781.1	1G	0G	1,914,352	4,709,208	2,794,856	4280	3,112,244	3	37	Yes	Transition interval spans a marker gap > 2 Mb (no diagnostic marker in between); transition position is not resolved within this interval; Flanking segments supported by 3-4 markers
A3N05	CM046781.1	0G	1G	7,821,452	7,826,918	5466	3,112,244	9151	37	5	No	Both flanking segments supported by >= 5 markers
A3N05	CM046781.1	1G	0G	7,836,069	7,849,016	12,947	9151	2,599,622	5	94	No	Both flanking segments supported

												by >= 5 markers
A3N05	CM046781.1	0G	1G	10,448,638	10,796,642	348,004	2,599,622	163,711	94	16	No	Both flanking segments supported by >= 5 markers
A3N05	CM046781.1	1G	2G	10,960,353	10,976,015	15,662	163,711	9344	16	5	No	Both flanking segments supported by >= 5 markers
A3N05	CM046781.1	2G	1G	10,985,359	10,998,477	13,118	9344	473,141	5	12	No	Both flanking segments supported by >= 5 markers
A3N05	CM046781.1	1G	2G	11,471,618	11,742,214	270,596	473,141	14,518	12	6	No	Both flanking segments supported by >= 5 markers
A3N05	CM046781.1	2G	1G	11,756,732	11,762,701	5969	14,518	163,901	6	7	No	Both flanking segments supported by >= 5 markers
A3N05	CM046781.1	1G	2G	11,926,602	11,927,085	483	163,901	10,734	7	16	No	Both flanking segments supported by >= 5 markers
A3N05	CM046781.1	2G	1G	11,937,819	11,966,078	28,259	10,734	6,692,703	16	179	No	Both flanking segments supported by >= 5

												markers
A3N05	CM046781.1	1G	0G	18,658,781	41,370,140	22,711,359	6,692,703	1,509,240	179	3	Yes	Transition interval spans a marker gap > 2 Mb (no diagnostic marker in between); transition position is not resolved within this interval; Flanking segments supported by 3-4 markers
A3N05	CM046781.1	0G	2G	42,879,380	44,038,805	1,159,425	1,509,240	7122	3	5	No	Flanking segments supported by 3-4 markers
A3N05	CM046781.1	2G	0G	44,045,927	44,348,511	302,584	7122	2,896,430	5	23	No	Both flanking segments supported by >= 5 markers
A3N05	CM046781.1	0G	1G	47,244,941	49,869,269	2,624,328	2,896,430	3,787,675	23	25	Yes	Transition interval spans a marker gap > 2 Mb (no diagnostic marker in between); transition

												position is not resolved within this interval; Both flanking segments supported by >= 5 markers
A3N05	CM046781.1	1G	0G	53,656,944	54,429,375	772,431	3,787,675	1,880,287	25	42	No	Both flanking segments supported by >= 5 markers
A3N05	CM046781.1	0G	1G	56,309,662	56,626,275	316,613	1,880,287	2,632,964	42	7	No	Both flanking segments supported by >= 5 markers
A3N05	CM046781.1	1G	0G	59,259,239	59,259,253	14	2,632,964	1,928,221	7	13	No	Both flanking segments supported by >= 5 markers
A3N05	CM046782.1	1G	2G	11,224,036	11,538,607	314,571	10,076,641	25,427	111	3	No	Flanking segments supported by 3-4 markers
A3N05	CM046782.1	2G	1G	11,564,034	11,603,297	39,263	25,427	453,673	3	6	No	Flanking segments supported by 3-4 markers
A3N05	CM046782.1	1G	2G	12,056,970	12,188,878	131,908	453,673	87	6	5	No	Both flanking segments supported

												by >= 5 markers
A3N05	CM046782.1	2G	1G	12,188,965	12,189,247	282	87	5,872,997	5	85	No	Both flanking segments supported by >= 5 markers
A3N05	CM046782.1	1G	0G	18,062,244	18,062,307	63	5,872,997	324,996	85	8	No	Both flanking segments supported by >= 5 markers
A3N05	CM046782.1	0G	1G	18,387,303	18,486,070	98,767	324,996	30,434,552	8	29	No	Both flanking segments supported by >= 5 markers
A3N05	CM046782.1	1G	2G	48,920,622	51,087,587	2,166,965	30,434,552	555	29	2	Yes	Transition interval spans a marker gap > 2 Mb (no diagnostic marker in between); transition position is not resolved within this interval; Adjacent to a segment supported by < 3 markers
A3N05	CM046782.1	2G	1G	51,088,142	52,311,200	1,223,058	555	4,619,015	2	33	No	Adjacent to a

												segment supported by < 3 markers
A3N05	CM046782.1	1G	0G	56,930,215	56,984,770	54,555	4,619,015	31	33	6	No	Both flanking segments supported by >= 5 markers
A3N05	CM046782.1	0G	1G	56,984,801	56,992,017	7216	31	290,765	6	9	No	Both flanking segments supported by >= 5 markers
A3N05	CM046782.1	1G	2G	57,282,782	57,294,563	11,781	290,765	46	9	3	No	Flanking segments supported by 3-4 markers
A3N05	CM046782.1	2G	1G	57,294,609	57,296,430	1821	46	2,676,047	3	20	No	Flanking segments supported by 3-4 markers
A3N05	CM046783.1	0G	1G	7,597,441	7,597,482	41	7,567,792	1,639,527	19	4	No	Flanking segments supported by 3-4 markers
A3N05	CM046783.1	1G	0G	9,237,009	9,925,782	688,773	1,639,527	3,511,104	4	24	No	Flanking segments supported by 3-4 markers
A3N05	CM046783.1	0G	2G	13,436,886	17,965,393	4,528,507	3,511,104	3	24	2	Yes	Transition interval spans a marker gap > 2 Mb (no diagnostic

												marker in between); transition position is not resolved within this interval; Adjacent to a segment supported by < 3 markers
A3N05	CM046783.1	2G	1G	17,965,396	18,997,077	1,031,681	3	17,906,026	2	15	No	Adjacent to a segment supported by < 3 markers
A3N05	CM046783.1	1G	2G	36,903,103	37,044,643	141,540	17,906,026	297,664	15	3	No	Flanking segments supported by 3-4 markers
A3N05	CM046783.1	2G	1G	37,342,307	37,958,318	616,011	297,664	12,940,054	3	18	No	Flanking segments supported by 3-4 markers
A3N05	CM046783.1	1G	2G	50,898,372	51,669,523	771,151	12,940,054	761,652	18	8	No	Both flanking segments supported by >= 5 markers
A3N05	CM046784.1	0G	2G	8,433,792	9,552,632	1,118,840	4,565,214	695,972	22	5	No	Both flanking segments supported by >= 5 markers

A3N05	CM046784.1	2G	1G	10,248,604	10,252,338	3734	695,972	4,387,497	5	4	No	Flanking segments supported by 3-4 markers
A3N05	CM046784.1	1G	2G	14,639,835	16,190,229	1,550,394	4,387,497	6,128,972	4	5	No	Flanking segments supported by 3-4 markers
A3N05	CM046784.1	2G	0G	22,319,201	24,203,900	1,884,699	6,128,972	29	5	3	No	Flanking segments supported by 3-4 markers
A3N05	CM046784.1	0G	2G	24,203,929	25,539,661	1,335,732	29	0	3	1	No	Adjacent to a single-marker segment
A3N05	CM046784.1	2G	1G	25,539,661	31,280,007	5,740,346	0	4,631,651	1	21	Yes	Transition interval spans a marker gap > 2 Mb (no diagnostic marker in between); transition position is not resolved within this interval; Adjacent to a single-marker segment
A3N05	CM046784.1	1G	0G	35,911,658	35,929,640	17,982	4,631,651	24,761	21	18	No	Both flanking segments supported by >= 5

												markers
A3N05	CM046784.1	0G	1G	35,954,401	36,034,318	79,917	24,761	422,387	18	34	No	Both flanking segments supported by >= 5 markers
A3N05	CM046785.1	0G	1G	3,370,952	4,785,560	1,414,608	1,147,947	5,575,928	3	12	No	Flanking segments supported by 3-4 markers
A3N05	CM046785.1	1G	0G	10,361,488	10,933,001	571,513	5,575,928	2,119,448	12	22	No	Both flanking segments supported by >= 5 markers
A3N05	CM046785.1	0G	1G	13,052,449	13,377,110	324,661	2,119,448	3,014,883	22	76	No	Both flanking segments supported by >= 5 markers
A3N05	CM046785.1	1G	0G	16,391,993	17,230,665	838,672	3,014,883	1,570,525	76	25	No	Both flanking segments supported by >= 5 markers
A3N05	CM046785.1	0G	1G	18,801,190	19,836,574	1,035,384	1,570,525	9,970,390	25	59	No	Both flanking segments supported by >= 5 markers
A3N05	CM046785.1	1G	2G	29,806,964	29,986,604	179,640	9,970,390	72,468	59	4	No	Flanking segments supported by 3-4 markers
A3N05	CM046785.1	2G	0G	30,059,072	30,094,675	35,603	72,468	3,076,992	4	9	No	Flanking

												segments supported by 3-4 markers
A3N05	CM046785.1	0G	1G	33,171,667	33,328,018	156,351	3,076,992	863,454	9	7	No	Both flanking segments supported by >= 5 markers
A3N05	CM046785.1	1G	0G	34,191,472	34,507,833	316,361	863,454	814,526	7	8	No	Both flanking segments supported by >= 5 markers
A3N05	CM046785.1	0G	1G	35,322,359	38,681,683	3,359,324	814,526	1,427,987	8	16	Yes	Transition interval spans a marker gap > 2 Mb (no diagnostic marker in between); transition position is not resolved within this interval; Both flanking segments supported by >= 5 markers
A3N05	CM046785.1	1G	2G	40,109,670	40,143,450	33,780	1,427,987	3,941,613	16	14	No	Both flanking segments supported by >= 5 markers

A3N05	CM046785.1	2G	1G	44,085,063	44,118,860	33,797	3,941,613	8,706,469	14	29	No	Both flanking segments supported by >= 5 markers

Ancestry states (0G, 1G, 2G: zero, one or two C. gigas-derived copies) are the Viterbi states of the three-state HMM (Section 2.5.3) underlying Figure 4. A segment is a maximal run of consecutive markers assigned the same state; each row is one pair of adjacent segments with different states on the same chromosome of the same individual (556 transitions: A3N01 106, A3N02 130, A3N03 108, A3N04 109, A3N05 103). The transition lies within the interval between the last supporting marker of the left segment and the first supporting marker of the right segment; inter-marker distance = right position − left position. No breakpoint position within this interval is estimated, and the interval is not an exact recombination breakpoint. Segment span = position of the last marker − position of the first marker of the segment (0 bp for single-marker segments); these spans are the coloured segments in Figure 4 and the denominator of the HMM fractions in Table S8. Supporting markers are moderate-tier diagnostic sites with read depth ≥ 6 in that individual; sites with lower depth in that individual were not used by the HMM. Because decoding is performed over consecutive informative markers, every listed transition is flanked by directly adjacent markers; no transition spans another assigned segment. Grey regions in Figure 4 between adjacent segments correspond to the listed inter-marker intervals; 69 transitions fall within inter-marker distances > 2 Mb, for which the transition position is not resolved. Notes classify the support of the flanking segments (single-marker segment; < 3 markers; 3–4 markers; ≥ 5 markers on both sides). Transitions adjacent to single-marker segments are weakly supported and are not equivalent to multi-marker tract boundaries.

Table S10. COI reference coordinates and diagnostic nucleotide positions underlying primer specificity.

Primer name	Target mitochondrial lineage	Primer sequence (5′–3′)	Reference accession and version	Primer-binding start (bp)	Primer-binding end (bp)	Strand (+/−)	Diagnostic nucleotide position in the reference (bp)	Diagnostic position within the primer (5′–3′)	Distance from the primer 3′ terminus (nt; 0 = terminal base)
C.gigas_3N_F3	*C. gigas*	AACTGGTCATTTTCACTACGTC	EU672831.1	1188	1209	+	1194	7	15
C.gigas_3N_F3	*C. gigas*	AACTGGTCATTTTCACTACGTC	EU672831.1	1188	1209	+	1197	10	12
C.gigas_3N_F3	*C. gigas*	AACTGGTCATTTTCACTACGTC	EU672831.1	1188	1209	+	1203	16	6
C.gigas_3N_F3	*C. gigas*	AACTGGTCATTTTCACTACGTC	EU672831.1	1188	1209	+	1206	19	3
C.gigas_3N_F3	*C. gigas*	AACTGGTCATTTTCACTACGTC	EU672831.1	1188	1209	+	1209	22	0
C.gigas_3N_R3	*C. gigas*	GATAATCAACTACTCGACGTGGT	EU672831.1	1377	1399	−	1377	23	0
C.gigas_3N_R3	*C. gigas*	GATAATCAACTACTCGACGTGGT	EU672831.1	1377	1399	−	1380	20	3
C.gigas_3N_R3	*C. gigas*	GATAATCAACTACTCGACGTGGT	EU672831.1	1377	1399	−	1395	5	18
C.angulata_3N_F3	*C. angulata*	GCATACGGAAATATGGGAATGTTC	NC_012648.1	868	891	+	873	6	18
C.angulata_3N_F3	*C. angulata*	GCATACGGAAATATGGGAATGTTC	NC_012648.1	868	891	+	876	9	15
C.angulata_3N_F3	*C. angulata*	GCATACGGAAATATGGGAATGTTC	NC_012648.1	868	891	+	882	15	9
C.angulata_3N_F3	*C. angulata*	GCATACGGAAATATGGGAATGTTC	NC_012648.1	868	891	+	888	21	3
C.angulata_3N_F3	*C. angulata*	GCATACGGAAATATGGGAATGTTC	NC_012648.1	868	891	+	891	24	0
C.angulata_3N_R3	*C. angulata*	ACCGCACCCATTGATAAAACGTAA	NC_012648.1	1203	1226	−	1203	24	0
C.angulata_3N_R3	*C. angulata*	ACCGCACCCATTGATAAAACGTAA	NC_012648.1	1203	1226	−	1206	21	3
C.angulata_3N_R3	*C. angulata*	ACCGCACCCATTGATAAAACGTAA	NC_012648.1	1203	1226	−	1209	18	6

C.angulata_3N_R3	*C. angulata*	ACCGCACCCATTGATAAAACGTAA	NC_012648.1	1203	1226	−	1210	17	7
C.angulata_3N_R3	*C. angulata*	ACCGCACCCATTGATAAAACGTAA	NC_012648.1	1203	1226	−	1221	6	18
C.angulata_3N_R3	*C. angulata*	ACCGCACCCATTGATAAAACGTAA	NC_012648.1	1203	1226	−	1224	3	21
C.hongkongensis_3N_F3	*C. hongkongensis*	GGATTTGGTAACTGGCTTATTCCA	NC_011518.2	307	330	+	309	3	21
C.hongkongensis_3N_F3	*C. hongkongensis*	GGATTTGGTAACTGGCTTATTCCA	NC_011518.2	307	330	+	327	21	3
C.hongkongensis_3N_F3	*C. hongkongensis*	GGATTTGGTAACTGGCTTATTCCA	NC_011518.2	307	330	+	330	24	0
C.hongkongensis_3N_R2	*C. hongkongensis*	ATAAGGGTGGATAGATTGTCCAT	NC_011518.2	453	475	−	453	23	0
C.hongkongensis_3N_R2	*C. hongkongensis*	ATAAGGGTGGATAGATTGTCCAT	NC_011518.2	453	475	−	456	20	3
C.hongkongensis_3N_R2	*C. hongkongensis*	ATAAGGGTGGATAGATTGTCCAT	NC_011518.2	453	475	−	462	14	9
C.hongkongensis_3N_R2	*C. hongkongensis*	ATAAGGGTGGATAGATTGTCCAT	NC_011518.2	453	475	−	465	11	12
C.hongkongensis_3N_R2	*C. hongkongensis*	ATAAGGGTGGATAGATTGTCCAT	NC_011518.2	453	475	−	468	8	15
C.hongkongensis_3N_R2	*C. hongkongensis*	ATAAGGGTGGATAGATTGTCCAT	NC_011518.2	453	475	−	471	5	18

Bases were read from the COI multiple sequence alignment used for primer design (33 sequences, 1,620 columns): *C. gigas*, n = 11 (G3N01–G3N05 and EU672831.1, KJ855241.1–KJ855245.1); *C. angulata*, n = 10 (A3N01–A3N05 and NC_012648.1, FJ841965.1, KJ855246.1, KJ855248.1, KJ855249.1); *C. hongkongensis*, n = 12 (H3N01–H3N05 and NC_011518.2, EU266073.1, EU672834.1, FJ593172.1, FJ593173.1, FJ841963.1, ON720160.1). Counts are given as observed sequences/examined sequences; no sequence had a gap or ambiguous base at any listed position. Coordinates are 1-based positions on the indicated complete mitochondrial genome record of the target lineage. In all 18 GenBank records COX1 is annotated from position 1 on the + strand, so the coordinates are also COX1 nucleotide positions. Alignment column numbers are not used as coordinates. Strand −: reverse primers anneal to the + strand of the reference; their 5′–3′ sequence is the reverse complement of reference positions start–end, and positions within these primers are counted from the primer 5′ end, which corresponds to the binding end coordinate. Bases are listed on the reference (+) strand; the corresponding primer base is given in Notes. Listed positions are all primer-binding positions at which at least one examined non-target sequence differs from the target-lineage reference base. Every primer matched all examined sequences of its target lineage without mismatch.

Table S11. D-statistics, block-jackknife standard errors, Z-scores and 95% confidence intervals for the estimates in Figure 6 and Section 3.6.

Outgroup (O)	Test population (P2)	Figure 6 element	Original test label	Jackknife blocks (n)	ABBA	BABA	D	SE (5-Mb block jackknife)	Z-score	95% CI lower	95% CI upper
C. hongkongensis	A3N (pooled, A3N01–A3N05)	Figure 6, observed pooled A3N (dashed line and shaded 95% CI)	A3N_pooled	119	633205	595036	0.0311	0.0025	12.38	0.0262	0.0360
C. hongkongensis	A3N01	Not plotted; individual value reported in Section 3.6	A3N01	119	633204	589418	0.0358	0.0033	10.90	0.0294	0.0423
C. hongkongensis	A3N02	Not plotted; individual value reported in Section 3.6	A3N02	119	638256	594300	0.0357	0.0033	10.92	0.0293	0.0421
C. hongkongensis	A3N03	Not plotted; individual value reported in Section 3.6	A3N03	119	625242	596725	0.0233	0.0035	6.61	0.0164	0.0303
C. hongkongensis	A3N04	Not plotted; individual value reported in Section 3.6	A3N04	119	629571	594691	0.0285	0.0031	9.28	0.0225	0.0345
C. hongkongensis	A3N05	Not plotted; individual value reported in Section 3.6	A3N05	119	639753	600046	0.0320	0.0028	11.64	0.0266	0.0374
C. hongkongensis	Synthetic triploid, 0.00 *C. gigas* + 3.00 *C. angulata* copies	Figure 6, synthetic calibration point	CAL_0.00	119	536359	538440	-0.0019	0.0014	-1.43	-0.0046	0.0007
C. hongkongensis	Synthetic triploid, 0.15 *C. gigas* + 2.85 *C. angulata* copies	Figure 6, synthetic calibration point	CAL_0.15	119	556093	541419	0.0134	0.0013	10.23	0.0108	0.0159
C. hongkongensis	Synthetic triploid, 0.30 *C. gigas* + 2.70 *C. angulata* copies	Figure 6, synthetic calibration point	CAL_0.30	119	575827	544398	0.0281	0.0013	21.16	0.0255	0.0307
C. hongkongensis	Synthetic triploid, 0.45 *C. gigas* + 2.55 *C. angulata* copies	Figure 6, synthetic calibration point	CAL_0.45	119	595560	547377	0.0422	0.0014	30.29	0.0394	0.0449
C. hongkongensis	Synthetic triploid, 0.60 *C. gigas* + 2.40 *C. angulata* copies	Figure 6, synthetic calibration point	CAL_0.60	119	615294	550356	0.0557	0.0015	37.48	0.0528	0.0586
C. hongkongensis	Synthetic triploid, 0.75 *C. gigas* + 2.25 *C. angulata* copies	Figure 6, synthetic calibration point	CAL_0.75	119	635028	553335	0.0687	0.0016	43.08	0.0656	0.0719
C. hongkongensis	Synthetic triploid, 0.90 *C. gigas* + 2.10 *C. angulata* copies	Figure 6, synthetic calibration point	CAL_0.90	119	654762	556314	0.0813	0.0017	47.49	0.0779	0.0846

C. hongkongensis	Synthetic triploid, 1.00 *C. gigas* + 2.00 *C. angulata* copies	Figure 6, synthetic calibration point	CAL_1.00	119	667918	558300	0.0894	0.0018	49.95	0.0859	0.0929
C. virginica	A3N (pooled, A3N01–A3N05)	Figure 6, observed pooled A3N (dashed line and shaded 95% CI)	A3N_pooled	119	128215	121517	0.0268	0.0021	12.79	0.0227	0.0309
C. virginica	A3N01	Not plotted; individual value reported in Section 3.6	A3N01	119	130431	117766	0.0510	0.0027	18.88	0.0457	0.0563
C. virginica	A3N02	Not plotted; individual value reported in Section 3.6	A3N02	119	129099	121332	0.0310	0.0029	10.76	0.0254	0.0367
C. virginica	A3N03	Not plotted; individual value reported in Section 3.6	A3N03	119	126012	122680	0.0134	0.0024	5.48	0.0086	0.0182
C. virginica	A3N04	Not plotted; individual value reported in Section 3.6	A3N04	119	127668	121917	0.0230	0.0026	9.01	0.0180	0.0280
C. virginica	A3N05	Not plotted; individual value reported in Section 3.6	A3N05	119	127868	123892	0.0158	0.0026	6.08	0.0107	0.0209
C. virginica	Synthetic triploid, 0.00 *C. gigas* + 3.00 *C. angulata* copies	Figure 6, synthetic calibration point	CAL_0.00	119	109104	110517	-0.0064	0.0017	-3.73	-0.0098	-0.0031
C. virginica	Synthetic triploid, 0.15 *C. gigas* + 2.85 *C. angulata* copies	Figure 6, synthetic calibration point	CAL_0.15	119	112543	111692	0.0038	0.0017	2.30	0.0006	0.0070
C. virginica	Synthetic triploid, 0.30 *C. gigas* + 2.70 *C. angulata* copies	Figure 6, synthetic calibration point	CAL_0.30	119	115982	112866	0.0136	0.0016	8.42	0.0104	0.0168
C. virginica	Synthetic triploid, 0.45 *C. gigas* + 2.55 *C. angulata* copies	Figure 6, synthetic calibration point	CAL_0.45	119	119421	114041	0.0230	0.0016	14.26	0.0199	0.0262
C. virginica	Synthetic triploid, 0.60 *C. gigas* + 2.40 *C. angulata* copies	Figure 6, synthetic calibration point	CAL_0.60	119	122860	115216	0.0321	0.0016	19.55	0.0289	0.0353
C. virginica	Synthetic triploid, 0.75 *C. gigas* + 2.25 *C. angulata* copies	Figure 6, synthetic calibration point	CAL_0.75	119	126300	116391	0.0408	0.0017	24.11	0.0375	0.0441
C. virginica	Synthetic triploid, 0.90 *C. gigas* + 2.10 *C. angulata* copies	Figure 6, synthetic calibration point	CAL_0.90	119	129739	117566	0.0492	0.0018	27.91	0.0458	0.0527
C. virginica	Synthetic triploid, 1.00 *C. gigas* + 2.00 *C. angulata* copies	Figure 6, synthetic calibration point	CAL_1.00	119	132032	118349	0.0546	0.0018	30.04	0.0511	0.0582

Topology D(P1, P2; P3, O) with P1 = *C. angulata* (ANG01–ANG05), P3 = *C. gigas* (ten diploid individuals) and O = *C. hongkongensis* (ten individuals) or *C. virginica* (ten individuals). D was computed with the frequency-based estimator of Durand et al. (2011) including both allelic orientations (Section 2.5.6) from biallelic SNPs of the reciprocal-mapping joint callset (*C. gigas* reference GCF_963853765.1).

Table S12. *Crassostrea hongkongensis* ancestry assigned to diploid reference panels and triploid groups by the two *C. hongkongensis* pairwise diagnostic-marker panels.

Group	Ploidy	Role in this panel	Individuals (n)	Markers with valid genotypes per individual, range (n)	Bias-corrected *C. hongkongensis* allele fraction (q), median (range)	*C. hongkongensis* copies on the group's own ploidy scale (q × ploidy), median (range)	Three-copy equivalent (3q), median (range)	Strict tier: three-copy equivalent (3q), median (range)
***C. gigas* vs. *C. hongkongensis* (13,282 moderate-tier markers)**								
C. gigas 2N	2	Marker-ascertainment panel	10	11,431–12,740	0.0069 (0.0039–0.0089)	0.014 (0.008–0.018)	0.021 (0.012–0.027)	0.004 (0.002–0.009)
C. angulata 2N	2	Diploid control not used for marker ascertainment	10	6,533–8,736	0.0557 (0.0413–0.0647)	0.111 (0.083–0.129)	0.167 (0.124–0.194)	0.149 (0.092–0.205)
C. hongkongensis 2N	2	Marker-ascertainment panel (target species)	10	8,946–12,010	0.9979 (0.9971–0.9994)	1.996 (1.994–1.999)	2.994 (2.991–2.998)	2.995 (2.987–2.996)
G3N	3	Triploid group	5	10,912–11,141	0.0310 (0.0218–0.0318)	0.093 (0.065–0.096)	0.093 (0.065–0.096)	0.042 (0.026–0.082)
A3N	3	Triploid group	5	10,792–11,439	0.0356 (0.0279–0.0425)	0.107 (0.084–0.127)	0.107 (0.084–0.127)	0.073 (0.021–0.101)
H3N	3	Triploid group (target species)	5	12,381–12,572	0.9970 (0.9901–0.9988)	2.991 (2.970–2.996)	2.991 (2.970–2.996)	2.996 (2.977–2.997)
***C. angulata* vs. *C. hongkongensis* (6,026 moderate-tier markers)**								
C. gigas 2N	2	Diploid control not used for marker ascertainment	10	4,900–5,443	0.0508 (0.0391–0.0601)	0.102 (0.078–0.120)	0.152 (0.117–0.180)	0.064 (0.010–0.124)
C. angulata 2N	2	Marker-ascertainment panel	10	4,965–5,376	0.0060 (0.0014–0.0128)	0.012 (0.003–0.026)	0.018 (0.004–0.038)	0.007 (0.005–0.015)
C. hongkongensis 2N	2	Marker-ascertainment panel (target species)	10	4,237–5,503	0.9960 (0.9907–0.9981)	1.992 (1.981–1.996)	2.988 (2.972–2.994)	2.991 (2.976–2.995)
G3N	3	Triploid group	5	5,304–5,464	0.0403 (0.0396–0.0477)	0.121 (0.119–0.143)	0.121 (0.119–0.143)	0.058 (0.008–0.068)
A3N	3	Triploid group	5	5,465–5,646	0.0198 (0.0128–0.0259)	0.060 (0.038–0.078)	0.060 (0.038–0.078)	0.009 (0.007–0.035)
H3N	3	Triploid group (target species)	5	5,582–5,736	0.9921 (0.9900–0.9964)	2.976 (2.970–2.989)	2.976 (2.970–2.989)	2.976 (2.966–2.996)

Species-diagnostic SNPs were ascertained exclusively from the two diploid reference panels specified for each pairwise comparison (ten individuals per species; Section 2.4). Reads from all individuals were aligned to the *C. gigas* reference assembly GCF_963853765.1, and valid genotype calls required read depth ≥ 8 and genotype quality ≥ 20. Values are reported for the moderate tier unless otherwise indicated. q denotes the reciprocal-orientation bias-corrected *C. hongkongensis* allele fraction defined in Section 2.4. Estimated dosage on the original ploidy scale is 2q for diploids and 3q for triploids. The three-copy-equivalent value (3q) standardizes both ploidy groups to the scale used in Figure 2 and Table S8 and is used for comparisons between diploid controls and triploid groups. Diploid *C. angulata* individuals in the *C. gigas*–*C. hongkongensis* comparison and diploid *C. gigas* individuals in the *C. angulata*–*C. hongkongensis* comparison were not used for marker ascertainment in the respective panels and served as non-target controls for evaluating background cross-assignment. Estimates within the marker-ascertainment panels are expected to approach zero or the full ploidy by construction and therefore do not constitute independent validation. The *C. hongkongensis* assignments reported for A3N and G3N in Figure 2 correspond to moderate-tier medians of 0.107 and 0.093 copies, respectively, obtained with the *C. gigas*–*C. hongkongensis* panel. On the common three-copy scale, these values were lower than the median assignment in the non-ascertainment diploid *C. angulata* controls (0.167). Together with their dependence on marker-panel selection and filtering stringency, these results support interpreting the minor assignments as background cross-assignment rather than evidence of *C. hongkongensis* ancestry. This comparison does not establish a formal analytical detection limit.
